# Direct synthesis of imino-*C*-nucleoside analogues and other biologically active iminosugars

**DOI:** 10.1038/ncomms7903

**Published:** 2015-04-23

**Authors:** Milan Bergeron-Brlek, Michael Meanwell, Robert Britton

**Affiliations:** 1 Department of Chemistry, Simon Fraser University, Burnaby, British Columbia, Canada V7G 1S2

## Abstract

Iminosugars have attracted increasing attention as chemical probes, chaperones and leads for drug discovery. Despite several clinical successes, their *de novo* synthesis remains a significant challenge that also limits their integration with modern high-throughput screening technologies. Herein, we describe a unique synthetic strategy that converts a wide range of acetaldehyde derivatives into iminosugars and imino-*C*-nucleoside analogues in two or three straightforward transformations. We also show that this strategy can be readily applied to the rapid production of indolizidine and pyrrolizidine iminosugars. The high levels of enantio- and diastereoselectivity, excellent overall yields, convenience and broad substrate scope make this an appealing process for diversity-oriented synthesis, and should enable drug discovery efforts.

## Abstract (web summary)

Iminosugars are biologically and medicinally important compounds but methods for their synthesis are often laborious. Here, the authors report a simple, rapid route for the enantioselective synthesis of multiple biologically active iminosugars and *C*-nucleoside analogues.

## Graphical abstract

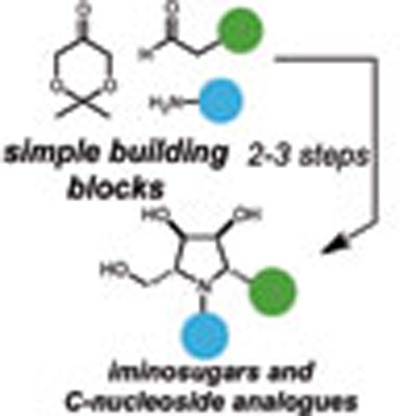

Iminosugars are naturally occurring carbohydrate mimics that inhibit many enzymes of medicinal interest^1^. Their biological activity is often attributed to a structural resemblance to the oxacarbenium ion-like transition states that occur during the enzymatic hydrolysis of carbohydrates^2^. As such, many iminosugars are potent inhibitors of glycosidases and glycosyltransferases^1^, and have been highlighted as lead candidates for the treatment of a variety of diseases, including cancer, diabetes, viral infections and lysosomal storage disorders (for example, Gaucher and Fabry disease)^1^,^3^. The most common naturally occurring iminosugars possess a polyhydroxylated pyrrolidine core and may be additionally annulated as in the pyrrolizidines (for example, **2**, Fig. 1), indolizidines (for example, **3**), or nortropanes^4^. A growing number of unnatural analogues of these compounds have also been reported as leads for drug discovery, including the imino-*C*-nucleosides developed by Schramm (for example, **1**)^5^,^6^ and β-hexosaminidase inhibitors developed by Wong^7^,^8^. Unfortunately, the incorporation of pyrrolidine iminosugars into chemical screening libraries or diversity-oriented synthesis (DOS) campaigns is problematic, as their syntheses are often lengthy, low-yielding, cost-intensive and limited by reliance on carbohydrate building blocks^9^. Thus, while several such pyrrolidine iminosugars have emerged as clinical candidates or drugs^1^, fundamental tools for their high-throughput synthesis are lacking. In fact, much of the success in imino-*C*-nucleoside synthesis^10^,^11^,^12^,^13^,^14^ (for example, **1** (ref. 6) and **4** (ref. 5)) has relied on the common building block **5** (refs 14, 15, 16). As evidenced by step counts provided in Fig. 1, the synthesis of pyrrolizidine- and indolizidine-based iminosugars (for example, **2**; (ref. 17) and **3**; (ref 18)) also remains a significant synthetic challenge.

We have reported preliminarily that when mixtures of the dioxanone **8**, an aliphatic aldehyde **6** and *N*-chlorosuccinimide (NCS) are treated with (*S*)-proline, a series of well-orchestrated reactions occur^19^. First, the aldehyde undergoes α-chlorination^20^, producing a racemic mixture of α-chloroaldehydes **7**. Second, an enantioselective proline-catalysed aldol reaction occurs between the dioxanone **8** and the α-chloroaldehyde (*R*)-**7**. Importantly, proline also catalyses racemization of the α-chloroaldehydes **7** and, consequently, this second step effects a dynamic kinetic resolution (DKR)^19^. Thus, this one-pot reaction transforms commodity chemicals **6** and **8** into carbohydrate building blocks **9** in excellent yield, diastereoselectivity and enanantioselectivity. Considering the spatial relationship between the chloromethine and carbonyl functions in **9**, these aldol adducts may also serve as building blocks^21^,^22^,^23^,^24^ for the synthesis of polyhydroxypyrrolidines via a reductive amination–annulation sequence (see grey box, Fig. 2). Such a strategy would allow for the conversion of virtually any acetaldehyde derivative **6** into an iminosugar **10** in two straightforward transformations from commodity chemicals, thus enabling their integration with modern high-throughput screening technologies.

Here we demonstrate that the reductive amination of a wide range of ketochlorohydrins **9** provides a rapid route to pyrrolidine iminosugars^8^,^11^,^25^,^26^,^27^,^28^,^29^,^30^,^31^,^32^, such as those depicted in Fig. 1. Importantly, this unique two- or three-step process requires no cryogenic, anhydrous or otherwise complicated experimental conditions. The demonstration of this strategy in several short syntheses of biologically active imino-*C*-nucleoside analogues, and indolizidine and pyrrolizidine iminosugars highlights its adaptability for DOS and the rapid preparation of iminosugar-based screening libraries^26^.

## Results

### Reductive amination of α-chlorination-DKR aldol products

The utility of the synthetic strategy outlined in Fig. 2 relies intimately on a diastereoselective reductive amination of aldol adducts **9**. Enders has reported^27^ that the reductive amination of related aldol adducts that lack a chloromethine function were non selective (dr<2:1) using NaB(OAc)_3_H. Likewise, Madsen found similar selectivities in the reductive amination of the corresponding *syn*-aldol adduct^33^. Bearing this in mind, we began by screening solvents and reducing agents, as well as the addition of acetic acid to the reductive amination of ketochlorohydrin **11** (Table 1) (ref. 19). In all cases, an excess of amine was required for complete imine formation and avoidance of competing ketone reduction (entry 1). As indicated in entry 2, the conditions reported by Enders^27^ delivered the amino alcohols **12a** and **12b** in good yield (82%), albeit low diastereoselectivity. The relative stereochemistry of **12a** was assigned based on analysis of ^3^*J*_H,H_ coupling constants and NOESY spectra recorded on the cyclic carbamate derived from the reaction of **12a** with carbonyldiimidazole. Use of NaB(CN)H_3_ resulted in an improved diastereomeric ratio of these products (dr ∼6:1) in both CH_2_Cl_2_ and MeCN (entries 3 and 4) and in tetrahydrofuran (THF) the 1,3-*syn* amino alcohol **12a** was produced as the only detectable diastereomer in near quantitative yield (entry 5). As summarized in entries 6 and 7, this optimized protocol proved general and also provided access to the corresponding *N*-allyl and *N*-propargyl amines **13a** and **14a**, in excellent yield and diastereoselectivity.

### Synthesis of pyrrolidine iminosugars

While the aminochlorohydrin **12a** did not cyclize directly, its high-yielding conversion into the pyrrolidine iminosugar **15** simply required heating in methanol, which also promoted acetonide removal (Fig. 3). Alternatively, this cyclization could be effected by heating **12a** in toluene with excess NaHCO_3_, which provided the orthogonally protected iminosugar **16**. Anticipating that the increased reactivity of a benzylchlorohydrin would favour a one-pot reductive amination–annulation process, the readily available aldol adduct **17** (ref. 19) was also treated with NaB(CN)H_3_ in a mixture of THF/HOAc. Following this optimized procedure, the orthogonally protected iminosugar **18** was produced directly and in excellent yield. Removal of both the acetonide and benzyl-protecting groups by hydrogenolysis in acidic methanol gave the imino-*C*-nucleoside analogue **4**. Considering the aldol adduct **17** is available in one step from phenyl acetaldehyde^19^, this three-step synthesis of **4**, a potent (*K*_i_=170 nM) transition-state analogue inhibitor of nucleoside hydrolase^5^, represents a significant advance.

### Scope of direct iminosugar synthesis

To further evaluate the scope of this direct iminosugar synthesis, we repeated the reactions described in Fig. 3 with several additional alkyl- and aryl-substituted ketochlorohydrins prepared in one step using our (*S*)-proline-catalysed α-chlorination-DKR aldol reaction^19^. It is noteworthy that the enantiomeric ketochlorohydrins are also readily prepared using the corresponding (*R*)-proline-catalysed reaction^19^. As indicated in Fig. 4, the reductive amination–annulation process is general and delivers a wide range of polyhydroxypyrrolidines **22**–**43** in good to excellent overall yield. A number of the orthogonally protected iminosugars depicted in Fig. 4 are crystalline and their structures were confirmed by X-ray crystallographic analysis (Supplementary Information). As expected (*vide supra*), the synthesis of alkyl-substituted iminosugars required an additional cyclization step, whereby the product of reductive amination was heated in MeOH or toluene with NaHCO_3_. Thus, this convenient process can be tailored for the production of orthogonally protected (**22**, **24**, **26**, **28** and **30**) or native iminosugars (**23**, **25**, **27**, **29** and **31**). Conversely, reductive amination of aryl-substituted chlorohydrins provided the corresponding iminosugars **18** and **36**–**39** directly and in excellent yield. It is notable that both the one- and two-step iminosugar syntheses proved tolerant of various functional groups. For example, electron deficient aryl (**36** and **37**), electron rich aryl (**38**) and heteroaryl substituents (**39**) were readily incorporated. Likewise, alkyl (**22**–**35**), branched alkyl (**24**), silyloxy alkyl (**32**), allyl (**26**), propargyl (**28**), primary alkyl chloride (**33**) and benzyl (**30**) groups were all compatible with the reaction sequence. The use of benzyl amine (**22**–**39**), allyl amine (**40**–**41**) or propargyl amine (**42**–**43**) also highlights the utility of this process for DOS and the potential for further elaboration of these iminosugars through metathesis, click, or cross coupling reactions. Finally, the mild reaction conditions and the protecting-group compatibility deserves note, as the 1,3-dioxane function in **34** and **35**, silyl protecting group in **32** and **43**, and acetonide function (conditions a)) in all substrates remained intact throughout the reaction sequence. Importantly, these readily available iminosugars share many features considered optimal for lead identification^34^,^35^,^36^,^37^, including MW <350 DA, CLogP <2, multiple chiral centres, heterocyclic rings and H-bond donors/acceptors. In addition, the ease with which the amine function and ring substituent (R^1^ and R^2^ in **20**) can be differentiated provides unique opportunities for further diversification.

### Short syntheses of polyhydroxy pyrrolizidines and indolizidines

Figure 5 highlights the further application of this convenient strategy to the rapid preparation of several structurally complex polyhydroxy indolizidine and pyrrolizidine alkaloids, including analogues of the glycosidase inhibitors hyacinthacine and steviamine. While several strategies could be exploited for the second annulation event, ready access to the *N*-allyl pyrrolidine **40**, alkyl chloride **33** and protected ketones **34** and **35** suggested annulation events involving ring closing metathesis^38^,^39^, alkylation^24^,^40^,^41^ or reductive amination^17^,^42^. For example, heating the dienylpyrrolidine **40** with the Hoveyda–Grubbs 2nd generation catalyst^43^ in toluene provided the unsaturated indolizidine **45** in excellent overall yield from 4-pentenal (**44**). Alternatively, starting with 6-chloropentanal (**46**) or 5-chloropentanal (**48**), α-chlorination-DKR aldol reactions^19^ followed by reductive amination and cyclization provided the chloroalkylpyrrolidine **33** and pyrrolizidine **49**, respectively. Conversion of **33** into the corresponding indolizidine **47** (ref. 44) required hydrogenolytic removal of the benzyl-protecting group and brief treatment with base. Completion of the total synthesis of 7a*-epi*-hyacinthacine A_1_ (**50**)^45^,^46^ simply involved hydrogenolysis of **49** in acidic media.

**Figure 1:**
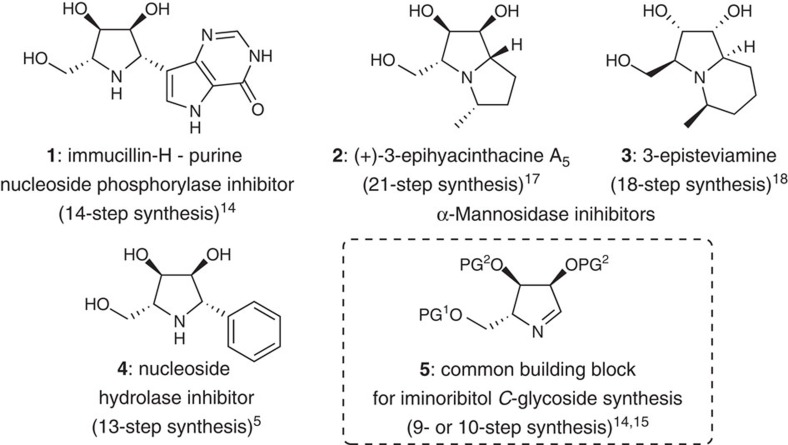
Biologically active iminosugars and the common building block 5 for imino-*C*-nucleoside synthesis. Immucillin-H (**1**) is a potent transition-state analogue inhibitor of purine nucleoside phosphorylase and a lead for the treatment of human T-cell leukaemia and lymphoma. The structurally related imino-*C*-nucleoside analogue **4** inhibits nucleoside hydrolase. In addition, the pyrrolizidine and indolizidine iminosugars **2** and **3** inhibit α-mannosidase, an enzyme target for anticancer therapies. The number of steps required to synthesize each of **1**–**4** and the synthetic building block **5** highlight the challenges faced when incorporating pyrrolidine iminosugars into chemical screening libraries and medicinal chemistry campaigns.

**Figure 2:**
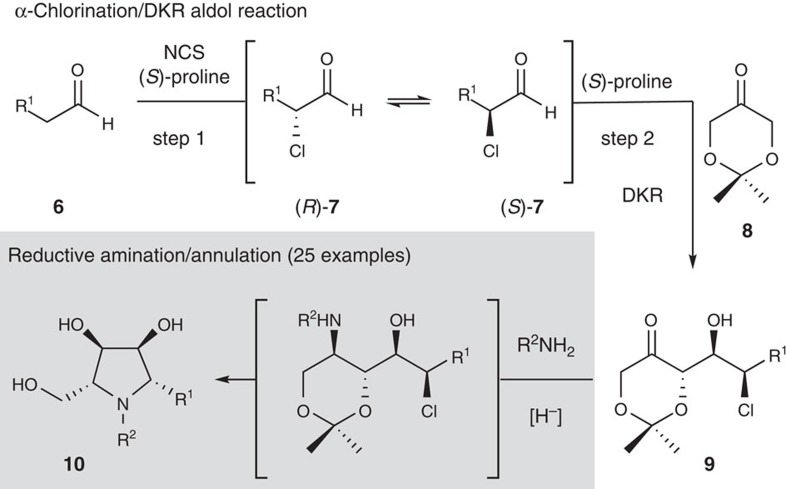
A convenient synthesis of polyhydroxypyrrolidine iminosugars. Organocatalytic tandem α-chlorination-DKR aldol reaction coupled with a reductive amination/annulation sequence to access iminosugars **10**.

**Figure 3:**
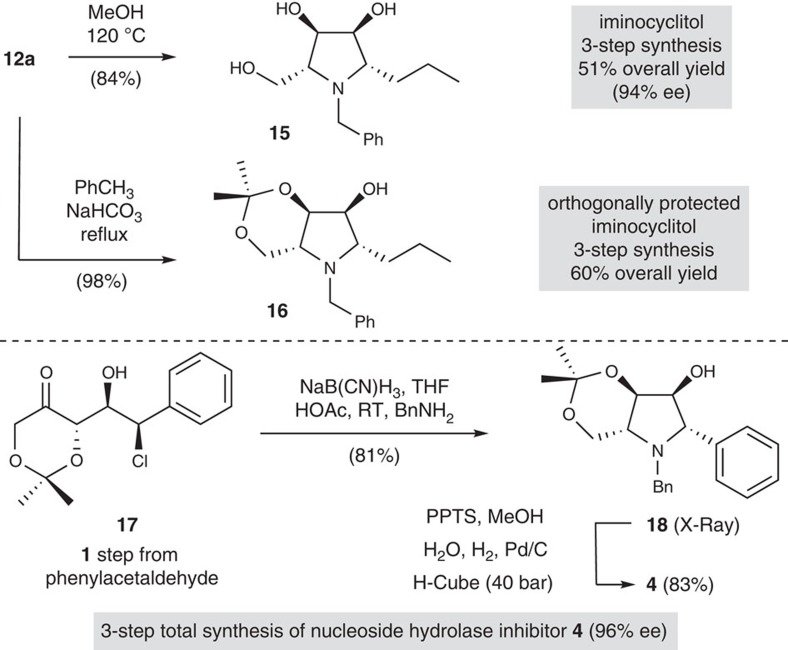
Synthesis of pyrrolidine iminosugars and imino-*C*-nucleoside analogues. A highly diastereoselective reductive amination of chlorohydrin aldol adducts followed by brief heating in methanol or toluene with NaHCO_3_ provides rapid access to native or differentially protected iminosugars. Reductive amination of the benzyl chloride-containing aldol adduct **17** leads directly to the protected iminosugar **18**, a precursor to the potent nucleoside hydrolase inhibitor **4**.

**Figure 4:**
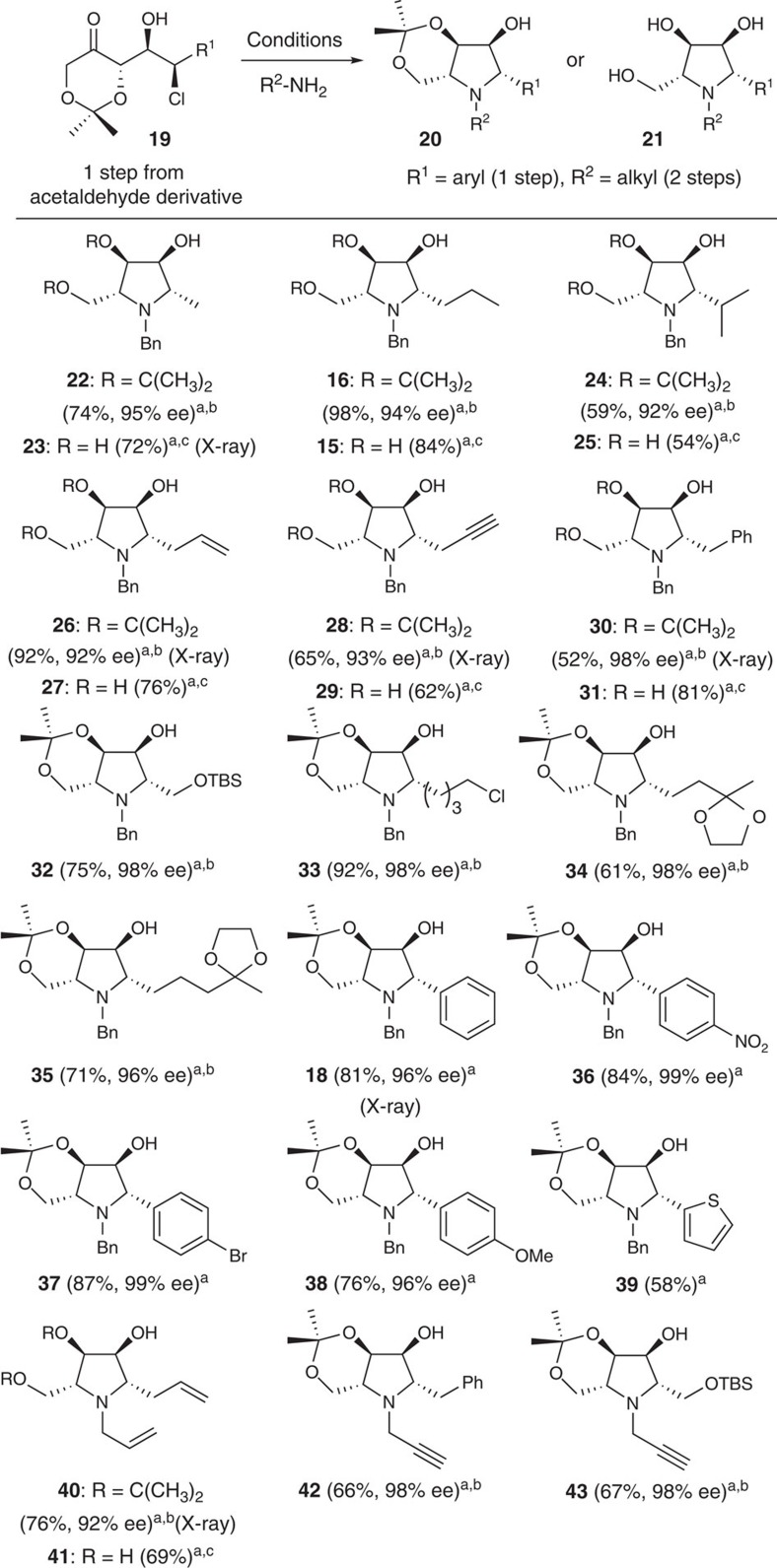
Scope of iminocyclitol synthesis. (**a**) amine (2.5 equivalents), AcOH, 4-Å mol sieves, THF; then NaB(CN)H_3_, room temperature; (**b**) NaHCO_3_, PhMe, 105 °C; (**c**) MeOH, 120 °C, (microwave reactor).

**Figure 5:**
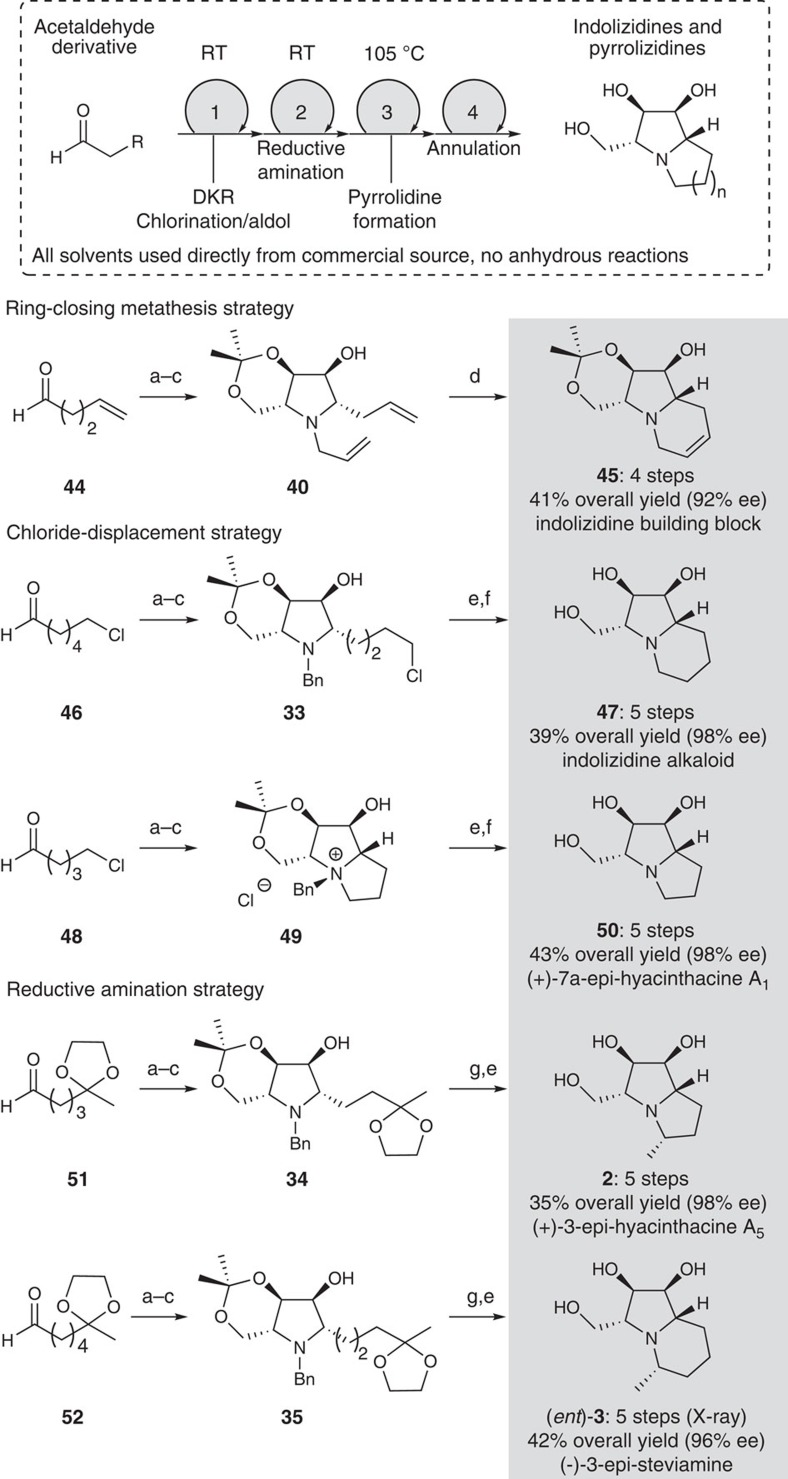
Total syntheses of indolizidine and pyrrolizidine iminocyclitols. (**a**) NCS, dioxanone **8**, (*S*)-proline (80 mol%), CH_2_Cl_2_, room temperature (RT); (**b**) benzyl amine or allyl amine (2.5 equivalents), AcOH, 4-Å mol sieves, THF; then NaB(CN)H_3_, RT; (**c**) NaHCO_3_, PhMe, 105 °C; (**d**) Hoveyda–Grubbs cat (2nd generation, 5 mol%), PhMe, 60 °C, 2 h; (**e**) H_2_ (90 bar), MeOH, 60 °C (H-Cube); (**f**) NaHCO_3_, MeOH, 80 °C, 16 h; then PPTS; (**g**) PPTS (cat), H_2_O, MeOH, 100 °C, 0.5 h (microwave reactor). NCS, *N*-chlorosuccinimide, PPTS, pyridinium *p*-toluenesulfonate.

The reductive amination strategy was explored in short syntheses of the hyacinthacine and steviamine analogues **2** (ref. 17) and *ent*-**3** (ref. 18). In both cases, the ketone function in the readily available pyrrolidines **34** and **35** was unveiled in concert with hydrogenolytic cleavage of the *N*-benzyl group, and the resulting iminium species (not shown) was reduced *in situ* to afford the products depicted as single diastereomers. Importantly, each of the total syntheses depicted in Fig. 5 requires 5 steps or less, originates with inexpensive and readily available chemicals, and is completed in a matter of days, which compares well with the reported syntheses of these and related compounds (see for example, Fig. 1).

## Discussion

In summary, a highly convergent synthesis of iminosugars has been developed that converts a wide range of acetaldehyde derivatives into polyhydroxypyrrolidines in two or three straightforward reactions and does not rely on carbohydrate building blocks. The application of this cost-effective process to the rapid synthesis of indolizidine and pyrrolizidine iminosugars also highlights its utility for the preparation of more structurally complex natural products and their analogues. Importantly, the excellent overall yields, diastereoselectivity and enantioselectivity, coupled with tunability of pharmacophoric features make this process well suited for chemical screening library and DOS campaigns.

## Methods

### Representative example of reductive amination/annulation sequence

Synthesis of aminochlorohydrin **18** and iminocyclitol **4**. To a stirred solution of **17** (ref. 19; 130 mg, 0.457 mmol) in THF (4.55 ml) was added BnNH_2_ (125 μl, 1.15 mmol) and glacial acetic acid (27.0 μl, 0.457 mmol), and the resulting mixture was stirred at 20 °C for 1 h. NaB(CN)H_3_ (72 mg, 1.15 mmol) was then added and the mixture was stirred for one additional hour. The reaction mixture was then diluted with CH_2_Cl_2_ to a concentration of 0.05 M and treated with water. The layers were separated and the organic layer was washed with brine, dried (MgSO_4_) and concentrated under reduced pressure. Purification of the crude product by flash chromatography (pentane-EtOAc 8:2) afforded pyrrolidine **18** (126 mg, 81% yield) as a crystalline solid. mp=108–111 °C (EtOH); *R*_*f*_ (pentane-EtOAc 6:4) 0.81; [*α*]_D_^20^=+11 (*c* 0.70 in CHCl_3_); infrared (neat): *ɛ*=3444, 2988, 2874, 1454, 1381, 1210, 1048, 853, 753 and 700 cm^−1^; ^1^H-nuclear magnetic resonance (^1^H-NMR; 600 MHz, CDCl_3_): *δ*=7.53 (*d*, *J*=7.4 Hz, 2H), 7.38 (*t*, *J*=7.5 Hz, 2H), 7.31–7.20 (*m*, 6H), 4.03 (*d*, *J*=4.4 Hz, 1H), 3.90 (*d*, *J*=12.9 Hz, 1H), 3.74 (*s*, 1H), 3.74 (*dd*, *J*=4.5 Hz, *J*=9.6 Hz, 1H), 3.47 (*d*, *J*=12.8 Hz, 1H), 3.46 (*dd*, *J*=10.5 Hz, 1H), 3.25 (*dd*, *J*=4.1 Hz, *J*=10.5 Hz, 1H), 2.89 (*ddd*, *J*=4.1 Hz, *J*=10.5 Hz, 1H), 2.31 (*s*, 1H), 1.42 (*s*, 3H) and 1.40 p.p.m. (*s*, 3H); ^13^C-NMR (151 MHz, CDCl_3_): *δ*=141.3, 139.2, 128.9, 128.5, 128.2, 127.4, 127.4, 127.3, 100.3, 76.9, 76.8, 74.1, 67.2, 59.6, 58.7, 29.2 and 19.8 p.p.m.; HRMS ESI (high-resolution mass spectrometry electrospray ionization) *m/z* calcd (calculated) for C_21_H_26_NO_3_ [M+H]^+^ 340.1907, found 340.1886.

### Preparation of the imino-*C*-nucleoside analogue 4

A solution of **18** (20 mg, 0.059 mmol) and pyridinium *p*-toluenesulfonate (15 mg, 0.059 mmol) in 1:1 H_2_O/MeOH (4.0 ml) was added to a microwave vial. The vial was sealed in a CEM Discover LabMate microwave reactor and the resulting mixture was heated at 100 °C (as monitored by a vertically focused infrared temperature sensor) for 30 min. The resulting solution was concentrated under reduced pressure and the crude product was used in the next reaction without further purification. A solution of the crude iminocyclitol *p*-toluenesulfonate salt in MeOH (20 ml) was passed twice through an H-Cube continuous-flow reactor using a 30 mm 10% Pd/C cartridge. Conditions: temperature=35 °C; flow rate=0.8 ml min^−1^; H_2_ pressure=40 bar. The resulting mixture was stirred with DOWEX 1X8-100 (HO^−^ form) for a further 30 min and the resin was removed by filtration. Concentration and purification of the crude product by flash chromatography on C_18_ silica gel (H_2_O) afforded iminoribitol **4** (10 mg, 83% yield over 2 steps) as a colourless oil. [*α*]_D_^20^=−31 (*c* 0.48 in MeOH); infrared (neat): *ɛ*=3306, 2918, 1560, 1494, 1454, 1406, 1347, 1081, 951, 757 and 699 cm^−1^; ^1^H-NMR (400 MHz, CD_3_OD): *δ*=7.45-7.42 (*m*, 2H), 7.37-7.32 (*m*, 2H), 7.27 (*ddt*, *J*=1.4 Hz, *J*=6.4 Hz, *J*=8.5 Hz, 1H), 4.01 (*d*, *J*=7.2 Hz, 1H), 3.97 (*dd*, *J*=4.7 Hz, *J*=6.0 Hz, 1H), 3.86 (*dd*, *J*=6.1 Hz, *J*=7.2 Hz, 1H), 3.74 (*d*, *J*=4.5 Hz, 2H) and 3.15 p.p.m. (*q*, *J*=4.5 Hz, 1H); ^13^C-NMR (151 MHz, CD_3_OD): *δ*=142.6, 129.5, 128.5, 128.1, 79.2, 73.6, 68.1, 66.8 and 63.2 p.p.m.; HRMS (ESI) *m/z* calcd for C_11_H_15_NO_3_ [M+H]^+^ 210.1125, found 210.1111.

## Additional information

**Accession codes**: The X-ray crystallographic coordinates for structures reported in this study have been deposited at the Cambridge Crystallographic Data Centre (CCDC), under deposition numbers 1038918-1038924. These data can be obtained free of charge from CCDC via www.ccdc.cam.ac.uk/data_request/cif.

**How to cite this article:** Bergeron-Brlek, M. *et al*. Direct synthesis of imino-*C*-nucleoside analogues and other biologically active iminosugars. *Nat. Commun.* 6:6903 doi: 10.1038/ncomms7903 (2015).

## Supplementary Material

Supplementary Figures, Supplementary Tables, Supplementary Methods and Supplementary References Supplementary Figures 1-59, Supplementary Tables 1-8, Supplementary Methods and Supplementary References

Supplementary Data 1 X Ray Structure (compound 18)

Supplementary Data 2 X Ray Structure (compound 23-OAc)

Supplementary Data 3 X Ray Structure (compound 26)

Supplementary Data 4 X Ray Structure (compound 28)

Supplementary Data 5 X Ray Structure (compound 30)

Supplementary Data 6 X Ray Structure (compound 40)

Supplementary Data 7 X Ray Structure (compound ent-3)

## Figures and Tables

**Table 1:**
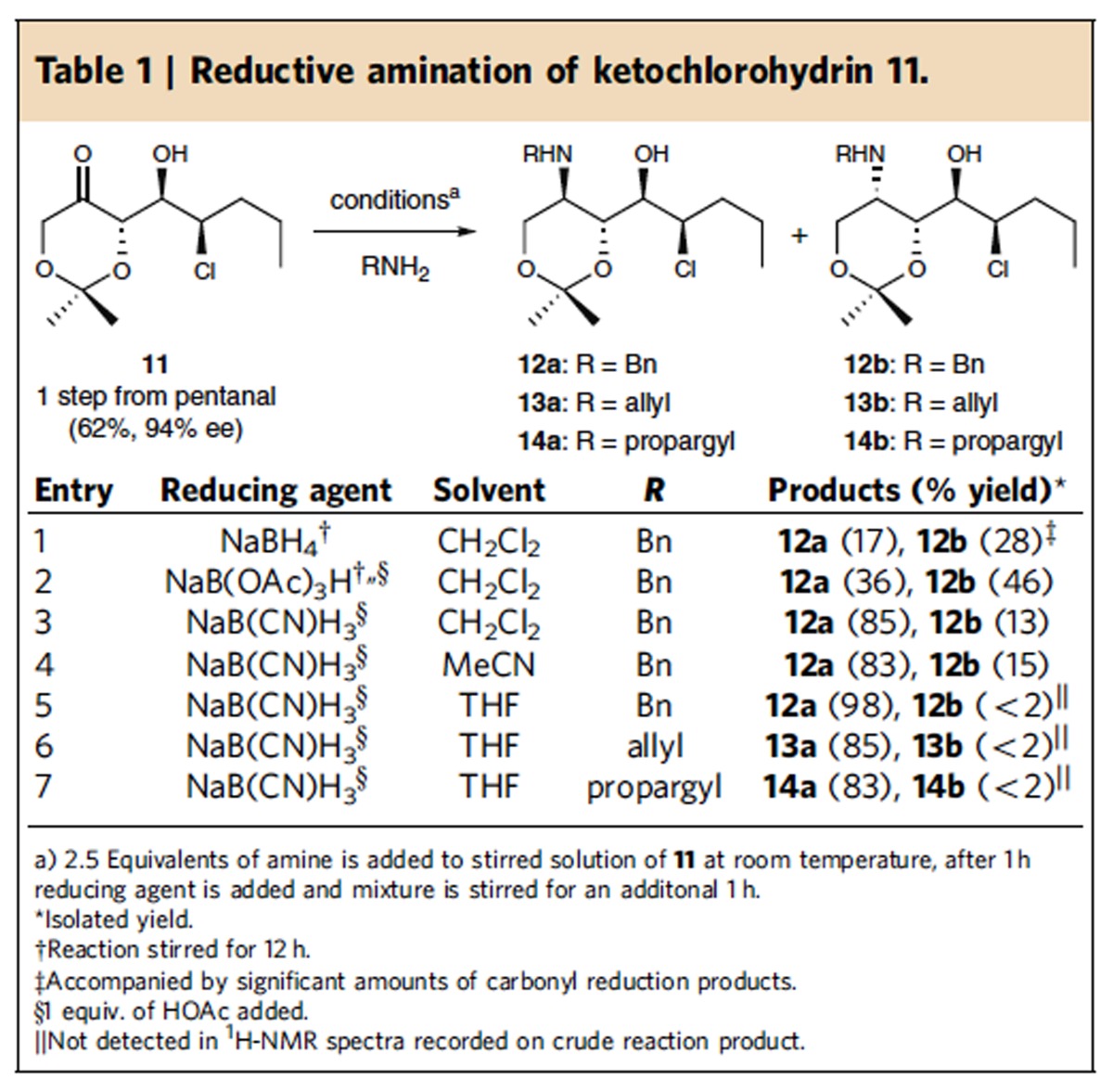
Reductive amination of ketochlorohydrin 11.
