# Supplementary material for: Direct synthesis of imino-C-nucleoside analogues and other biologically active iminosugars
Source: Nat Commun. 2015 Apr 23;6:6903. doi: 10.1038/ncomms7903 (PMC4558570; doi:10.1038/ncomms7903)
Supplement: Supplementary Figures, Supplementary Tables, Supplementary Methods and Supplementary References — Supplementary Figures 1-59, Supplementary Tables 1-8, Supplementary Methods and Supplementary References [file ncomms7903-s1.pdf]

Supplementary Figures

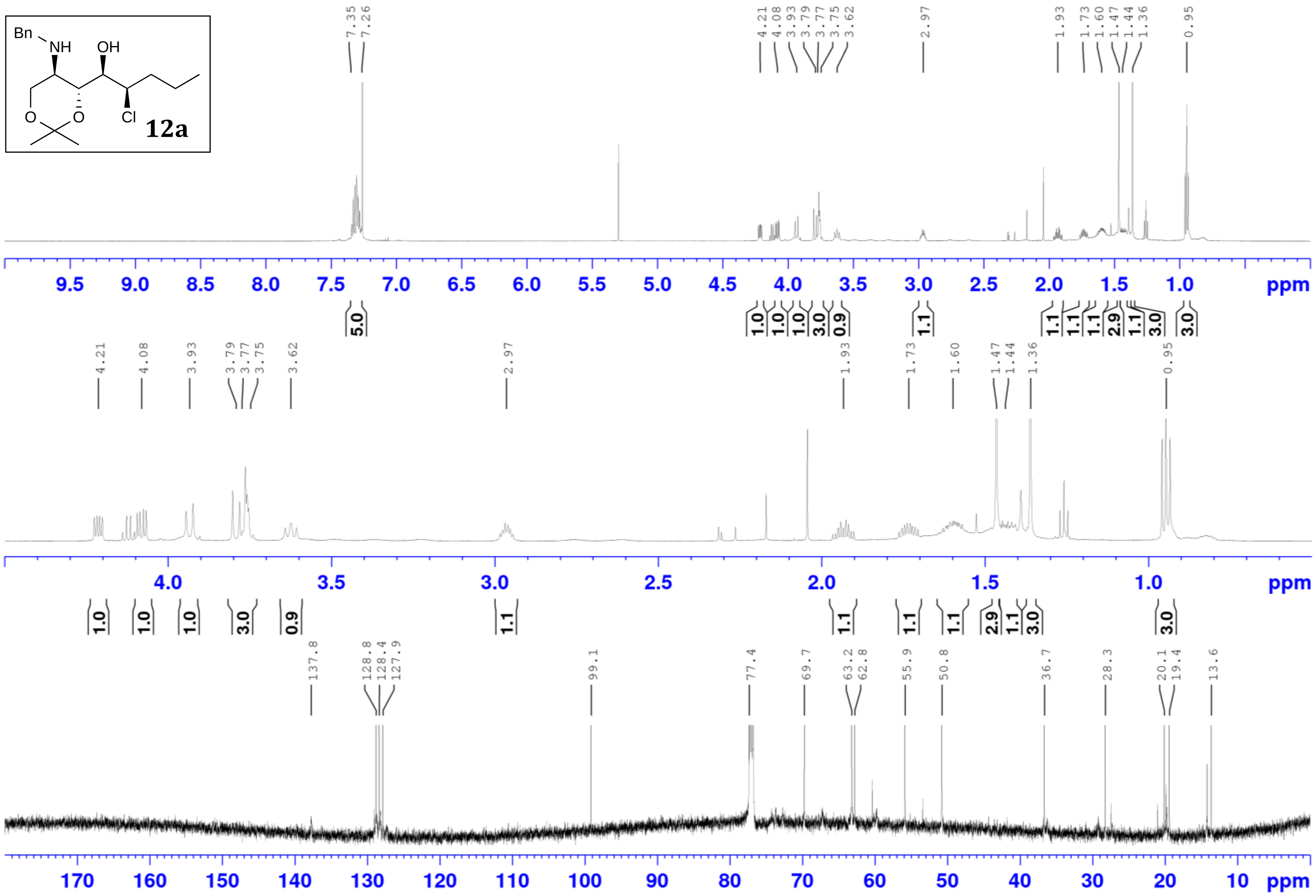

Supplementary Figure 1. ^{1}H **and** ^{13}C **NMR of compound 12a**

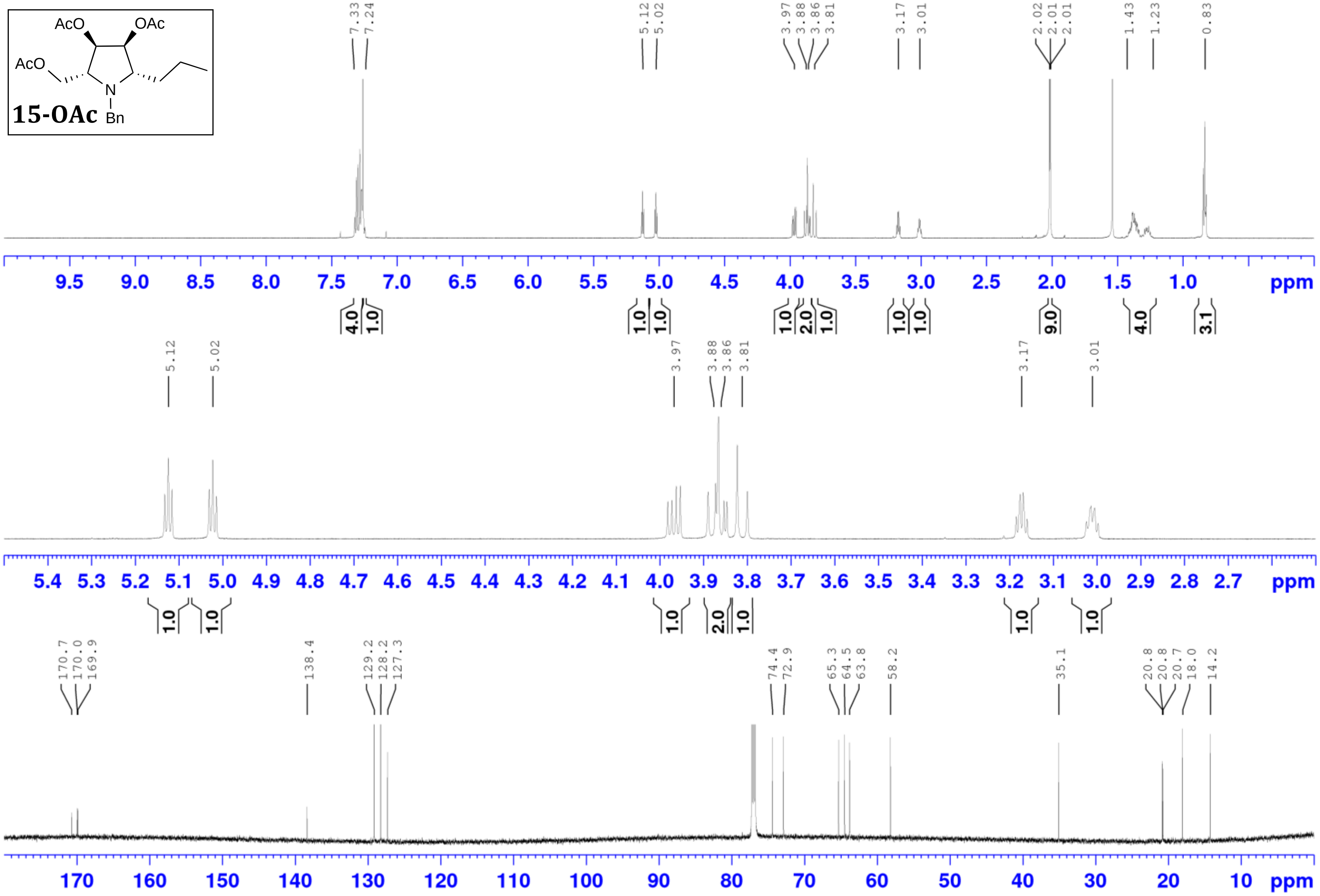

Supplementary Figure 2. ^{1}H and ^{13}C NMR of compound 15-OAc

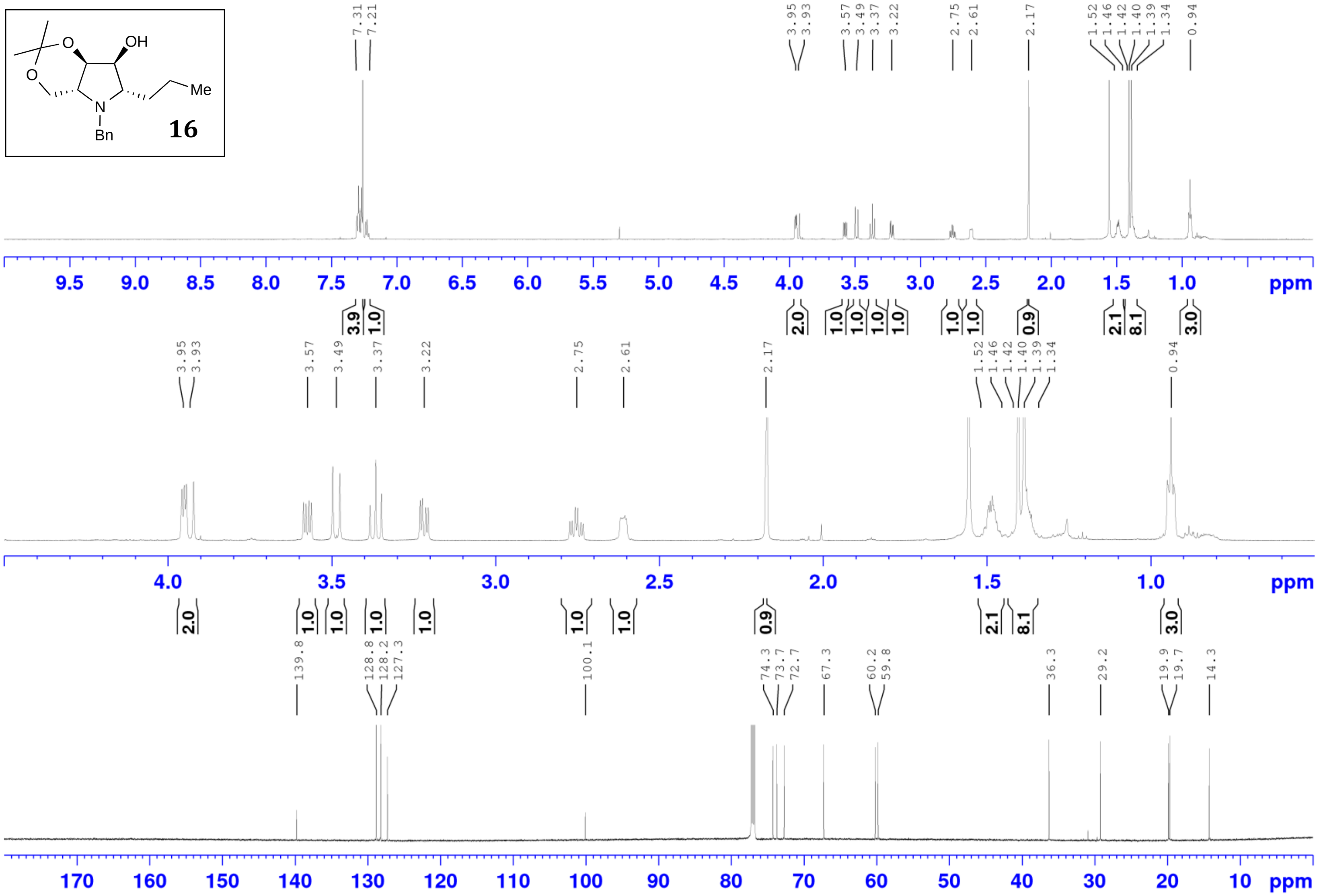

Supplementary Figure 3. ^{1}H **and** ^{13}C **NMR of compound 16**

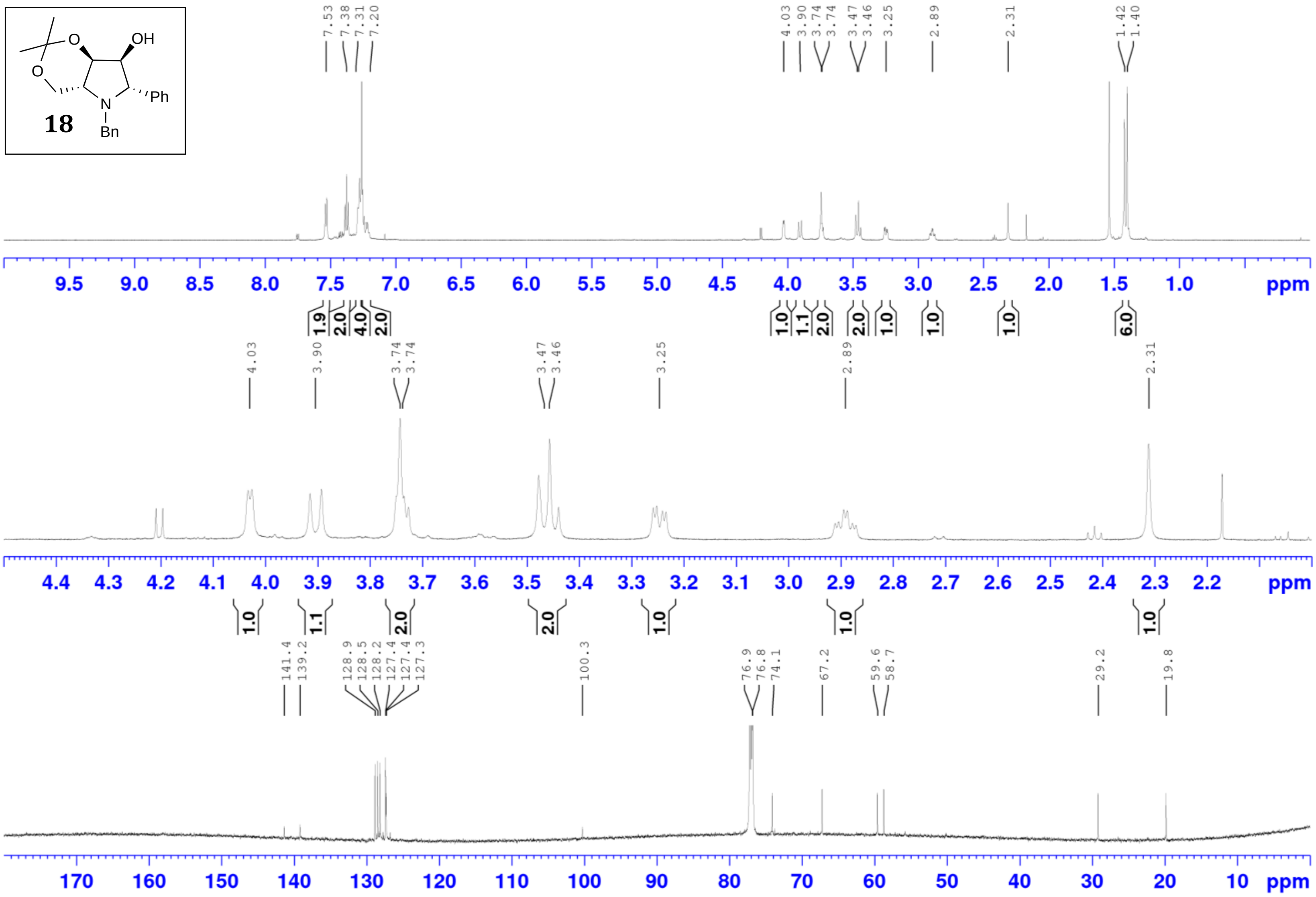

Supplementary Figure 4. ^{1}H and ^{13}C NMR of compound 18

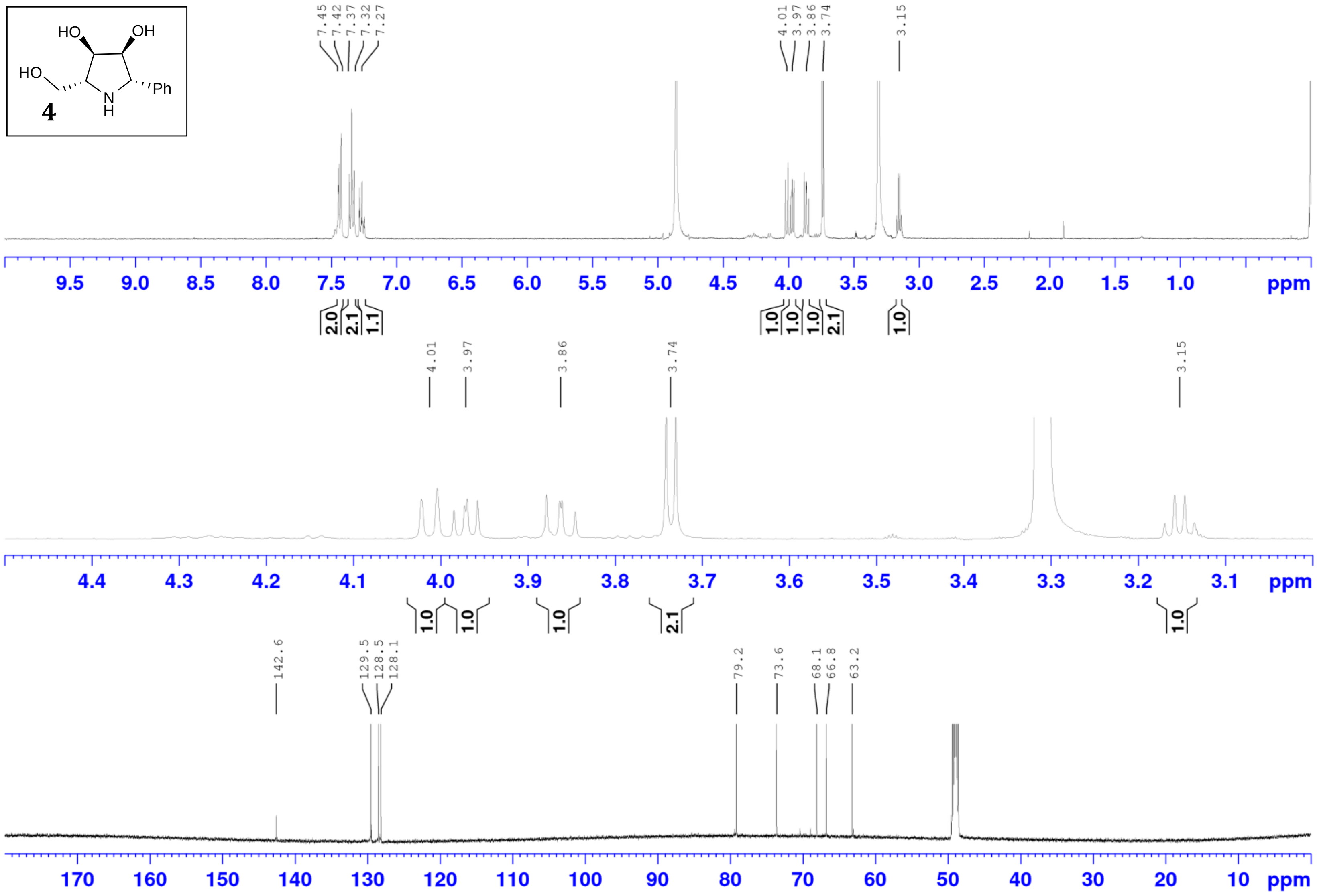

Supplementary Figure 5. ^{1}H **and** ^{13}C **NMR of compound 4**

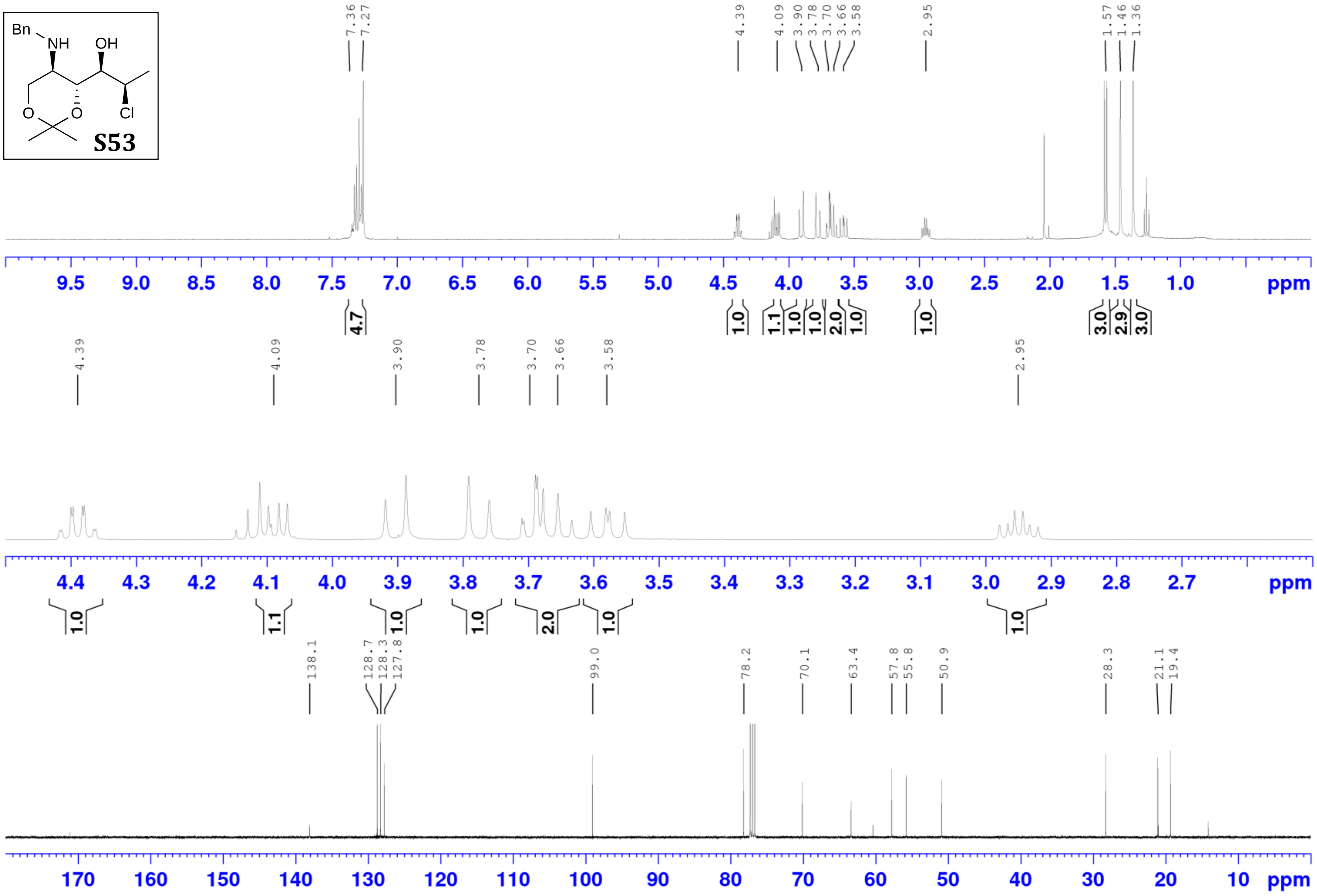

Supplementary Figure 6. ¹H and ¹³C NMR of compound S53

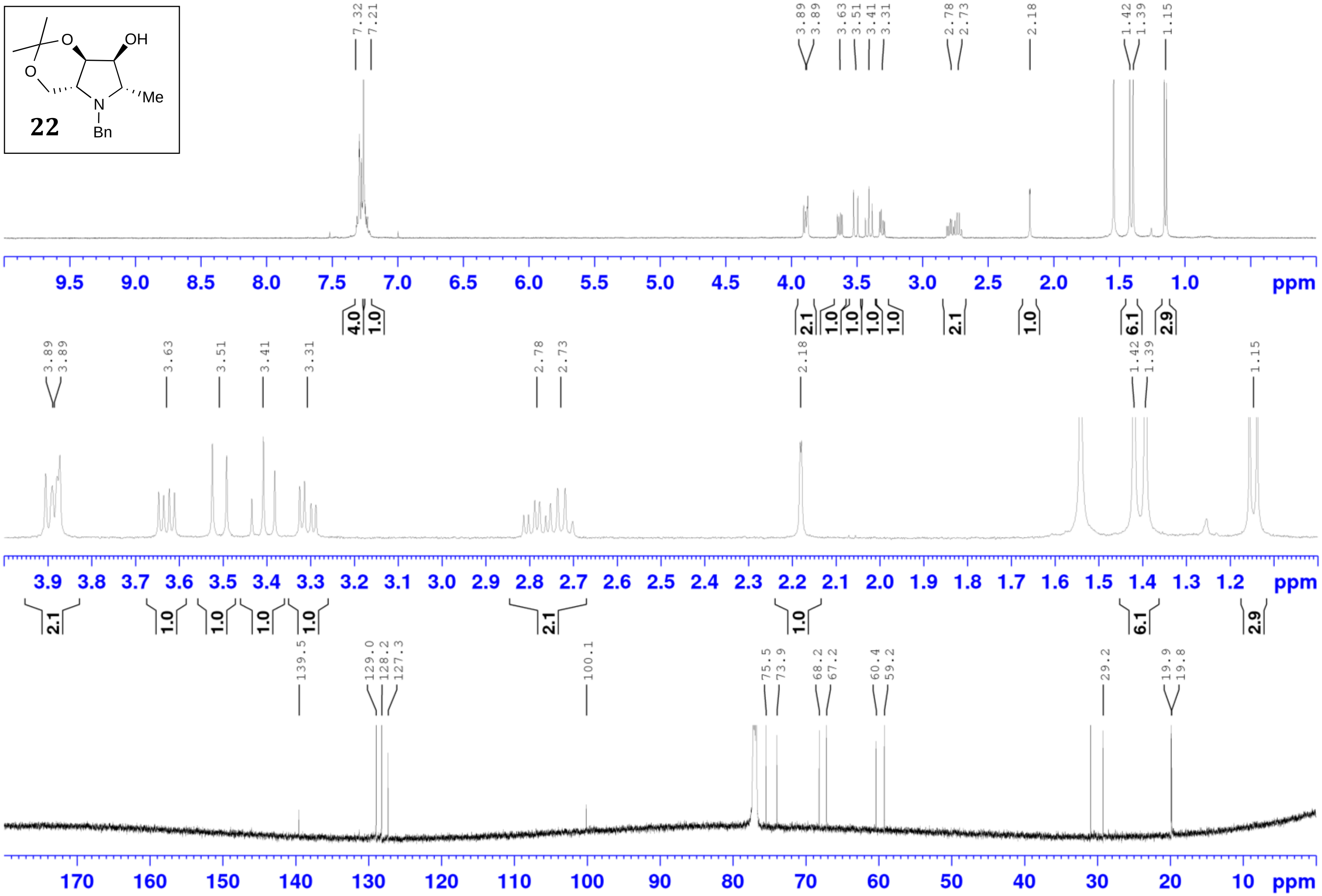

Supplementary Figure 7. ^{1}H and ^{13}C NMR of compound 22

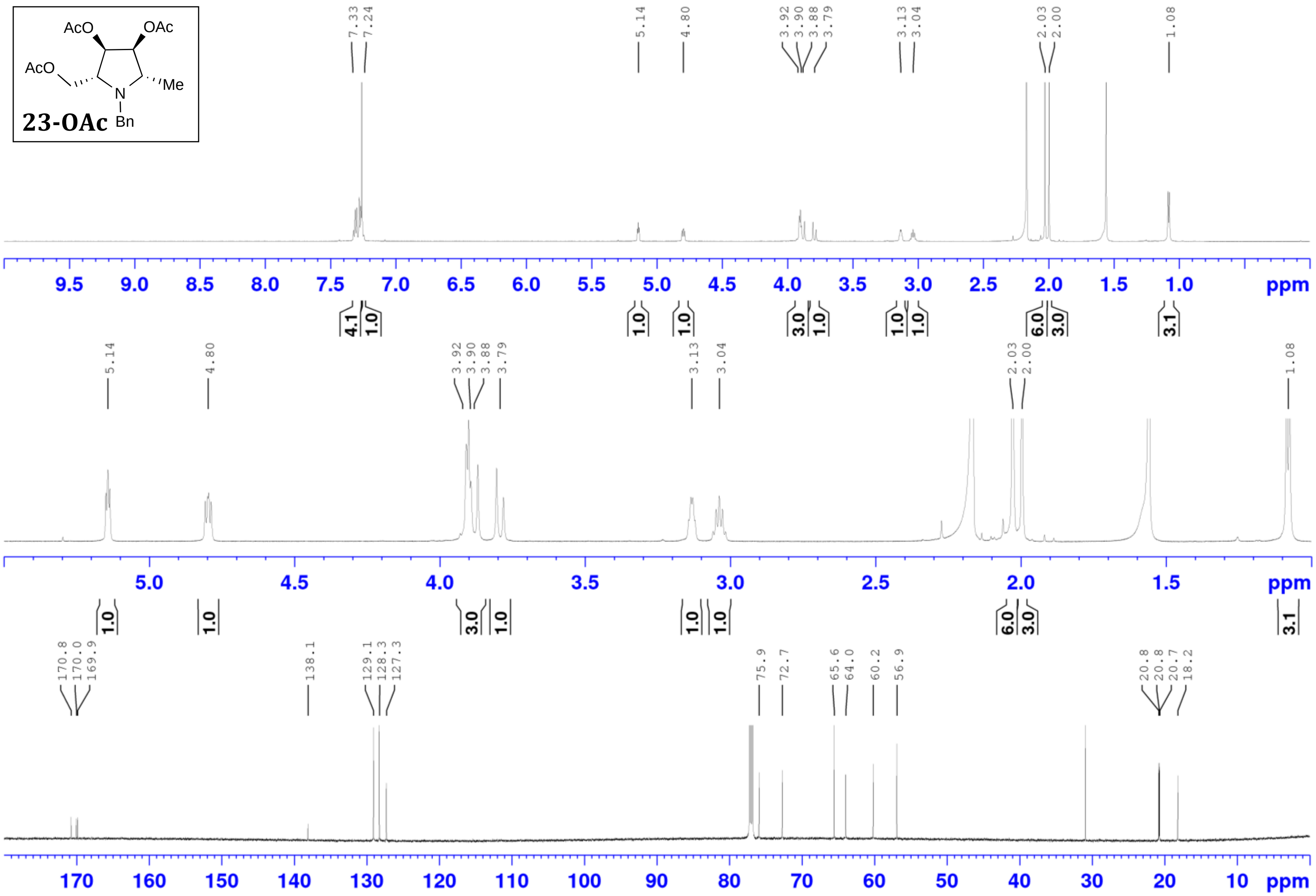

Supplementary Figure 8. ^{1}H **and** ^{13}C **NMR of compound 23-OAc**

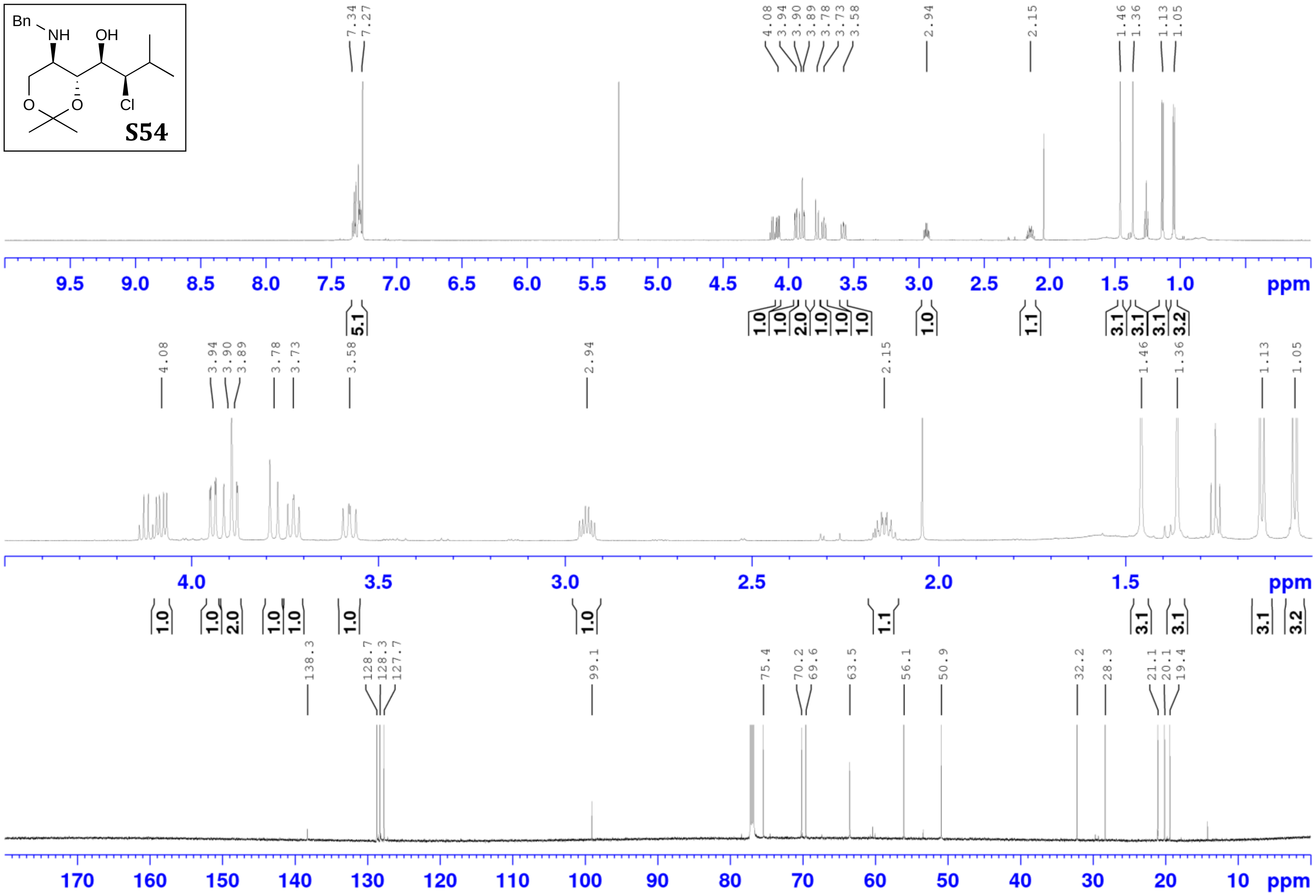

Supplementary Figure 9. ^{1}H and ^{13}C NMR of compound S54

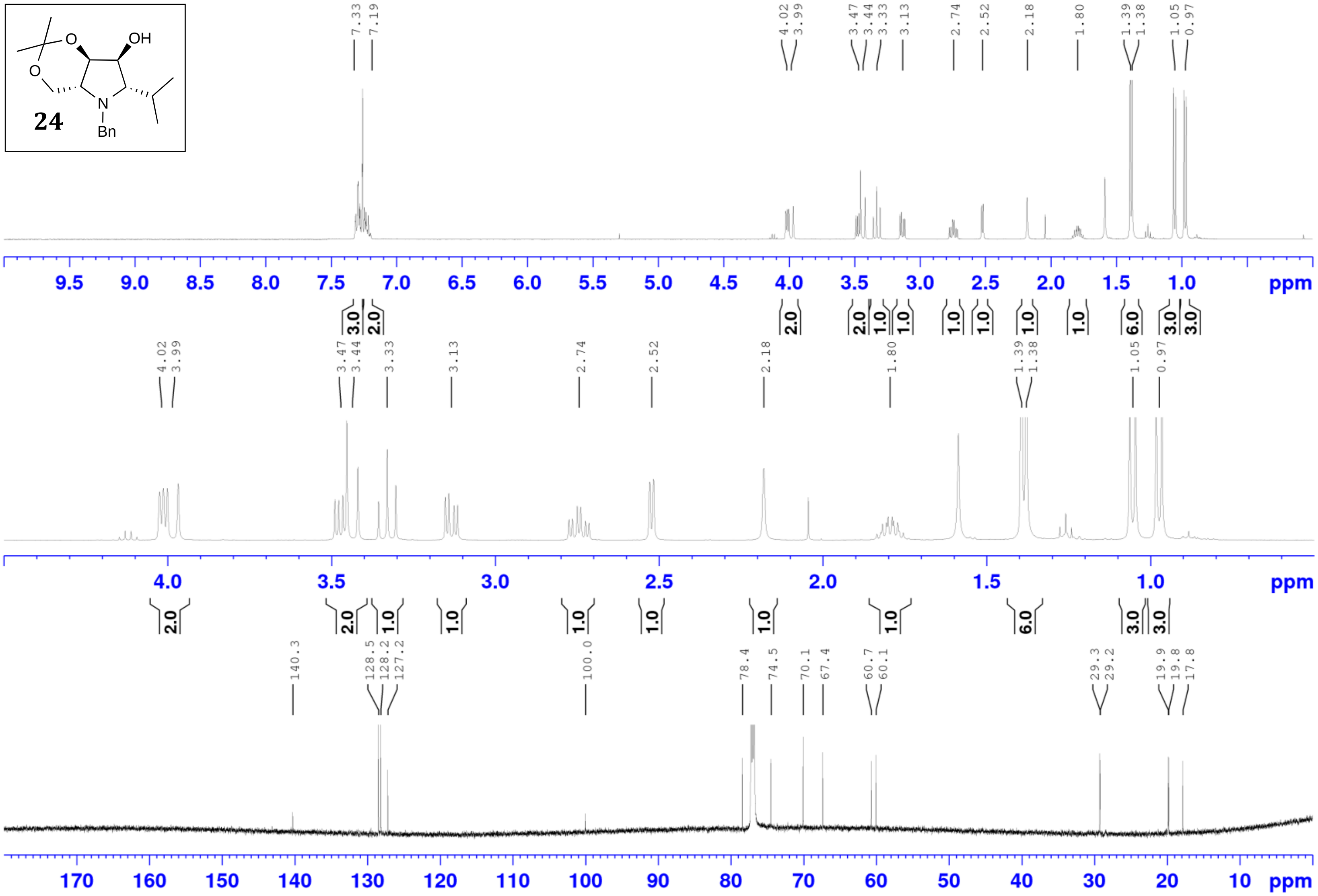

Supplementary Figure 10. ^{1}H and ^{13}C NMR of compound 24

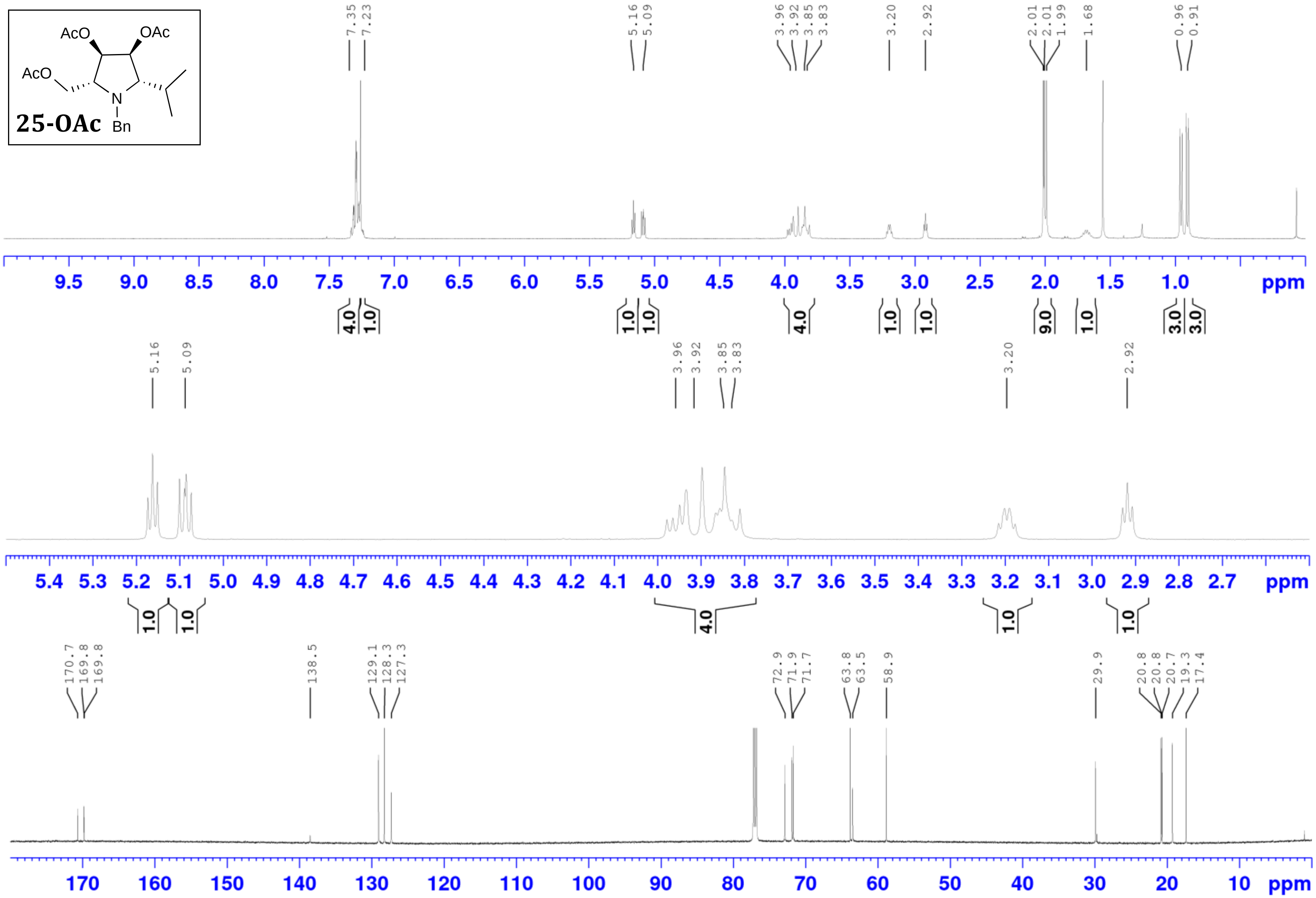

Supplementary Figure 11. ^{1}H and ^{13}C NMR of compound 25-OAc

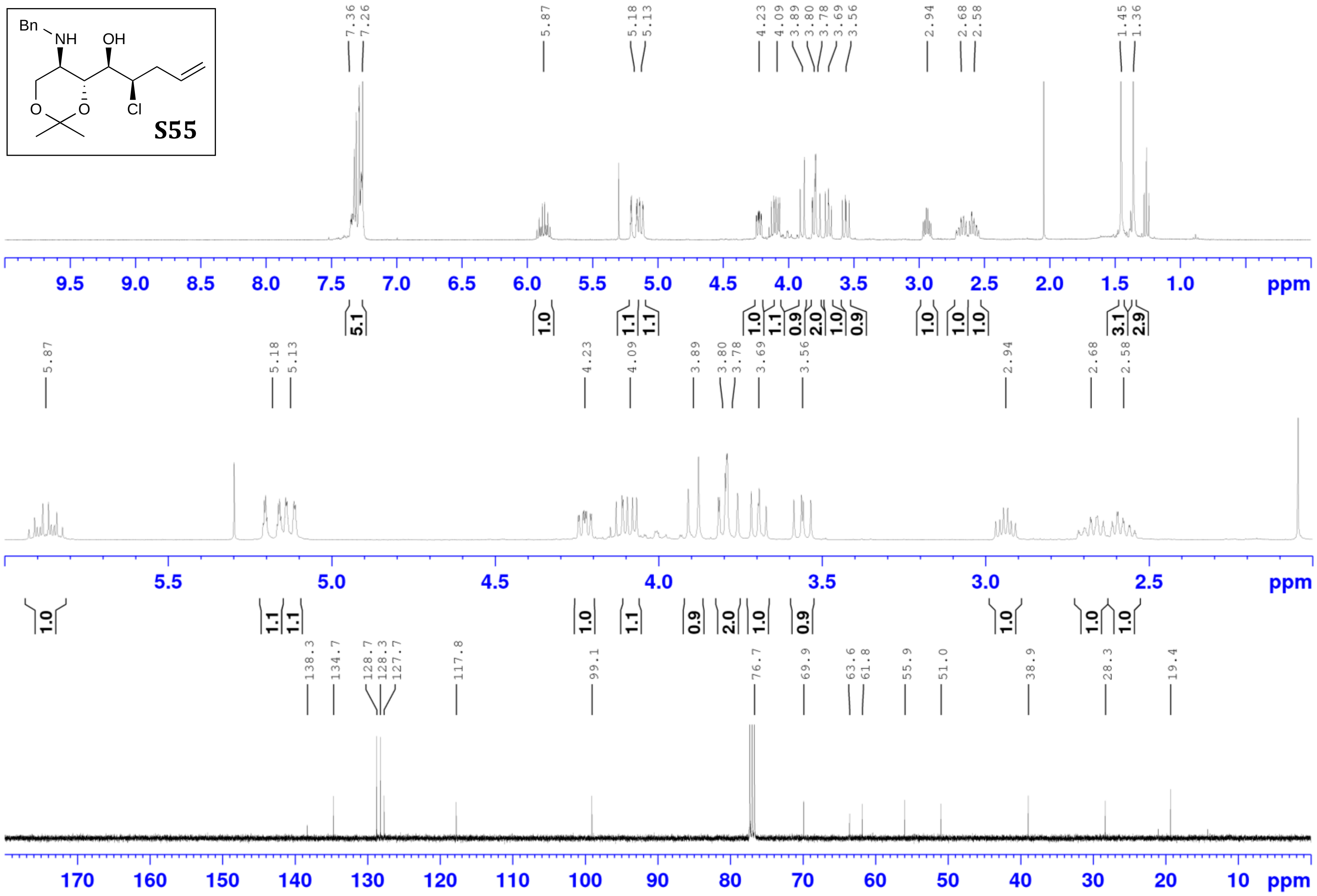

Supplementary Figure 12. ^{1}H and ^{13}C NMR of compound S55

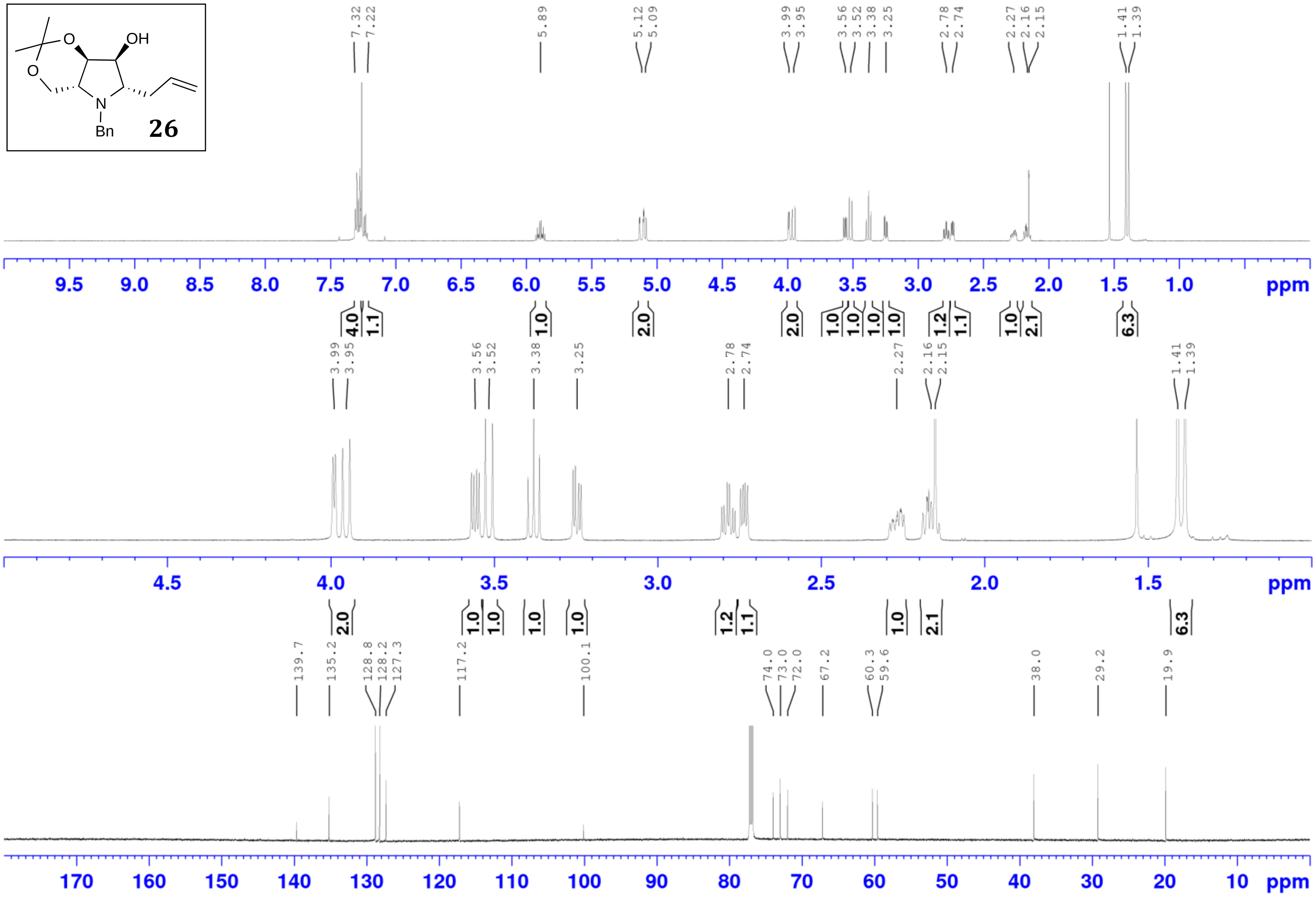

Supplementary Figure 13. ^{1}H and ^{13}C NMR of compound 26

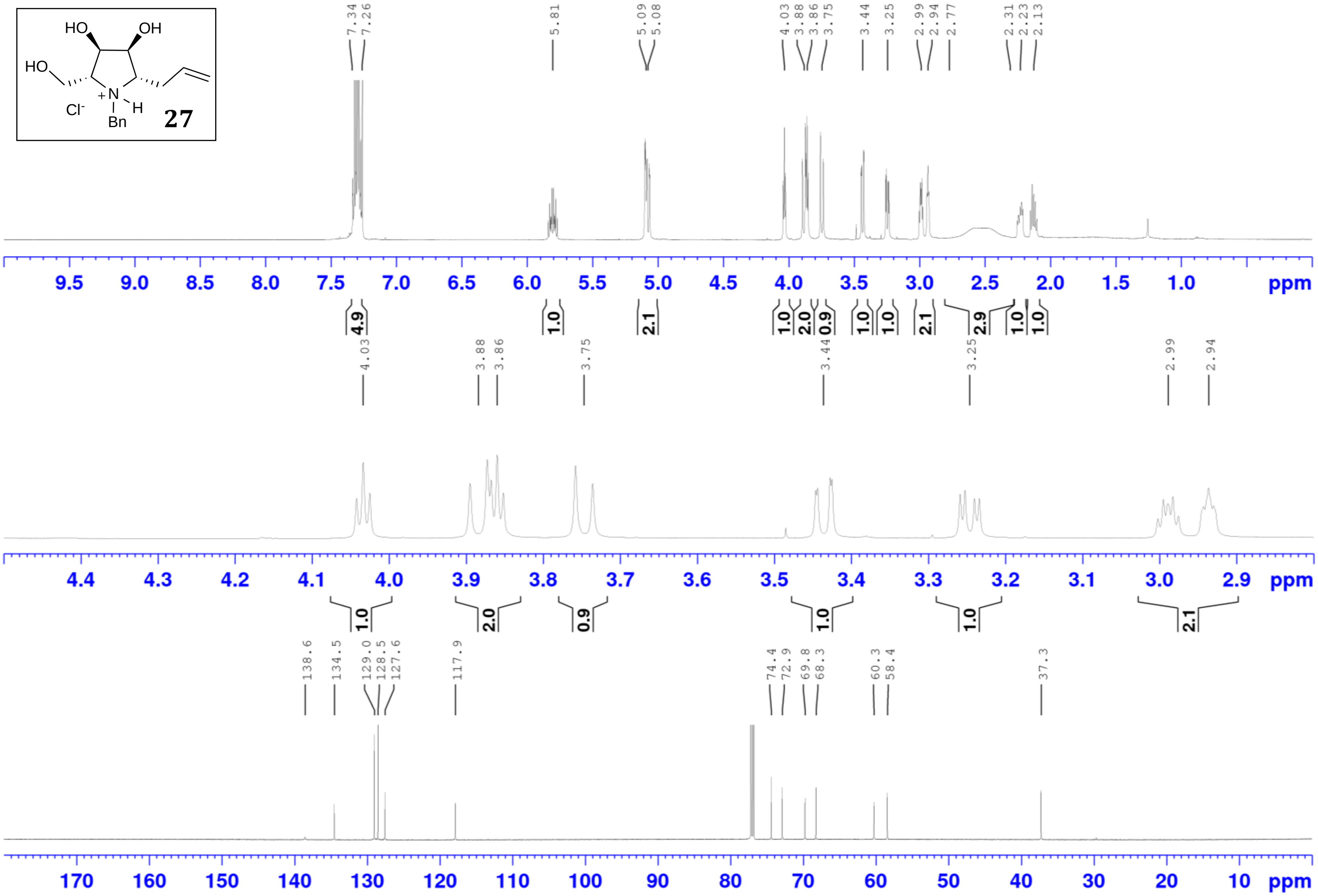

Supplementary Figure 14. ¹H and ¹³C NMR of compound 27

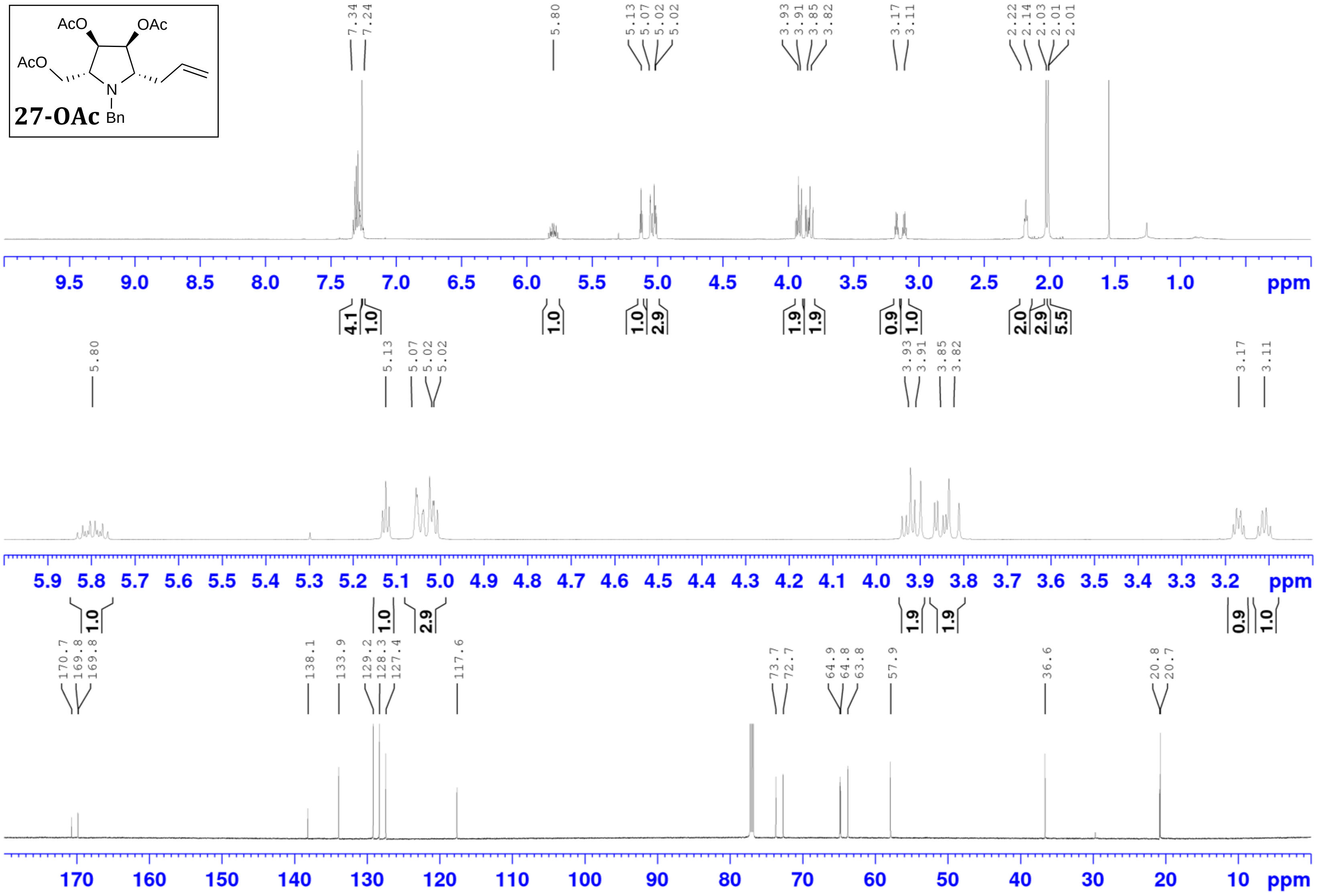

Supplementary Figure 15. ^{1}H **and** ^{13}C **NMR of compound 27-OAc**

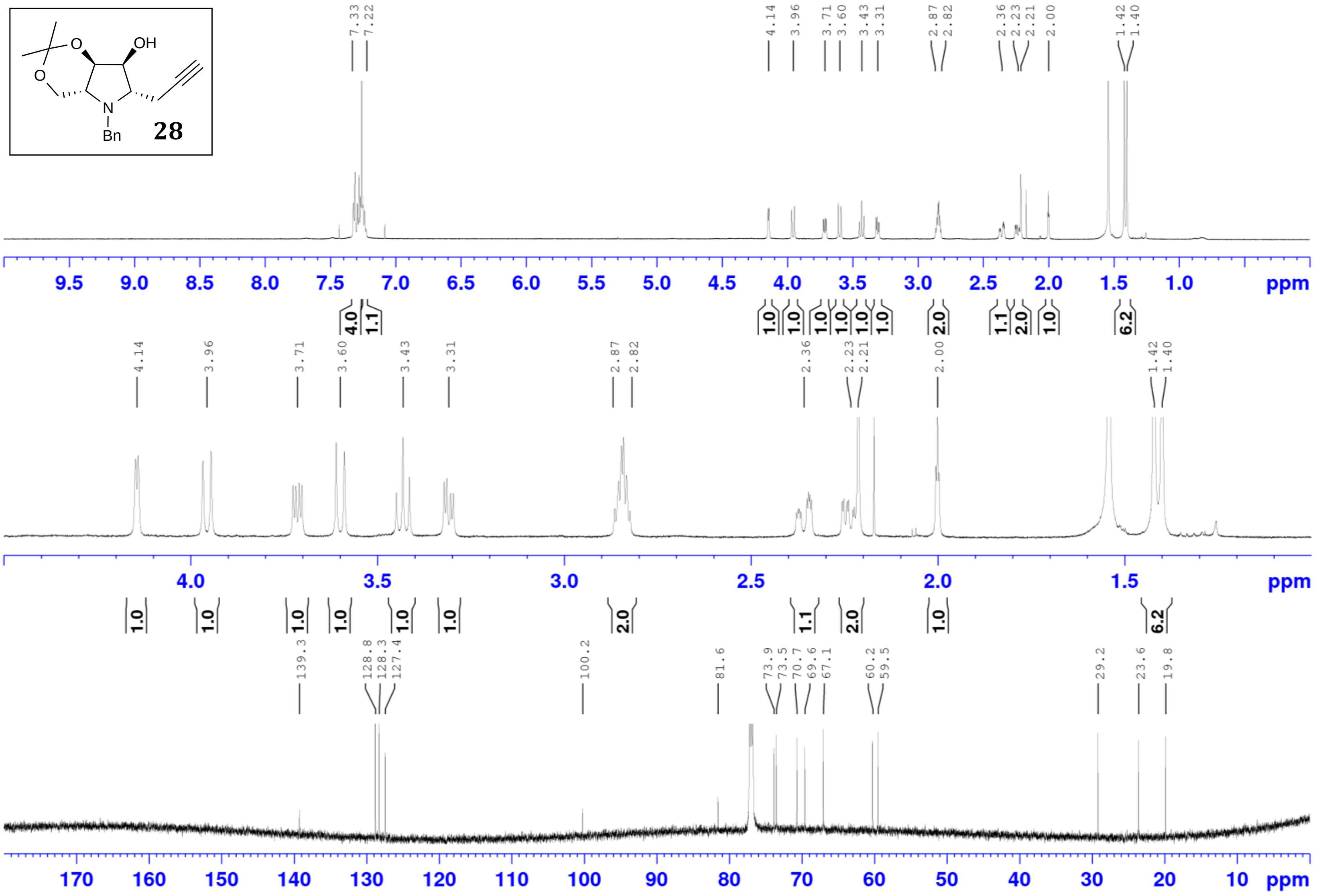

Supplementary Figure 16. ^{1}H and ^{13}C NMR of compound 28

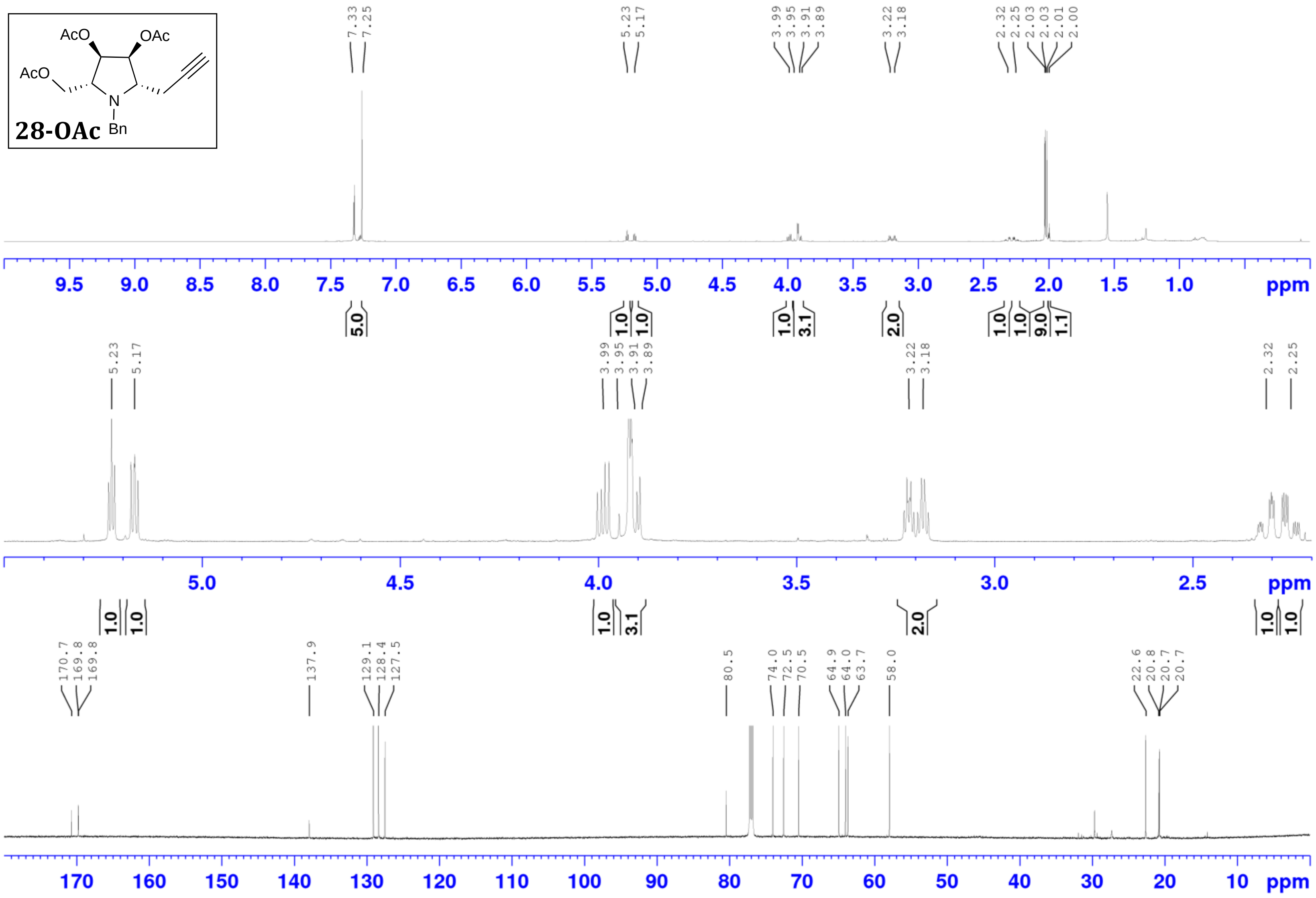

Supplementary Figure 17. ^{1}H and ^{13}C NMR of compound 28-OAc

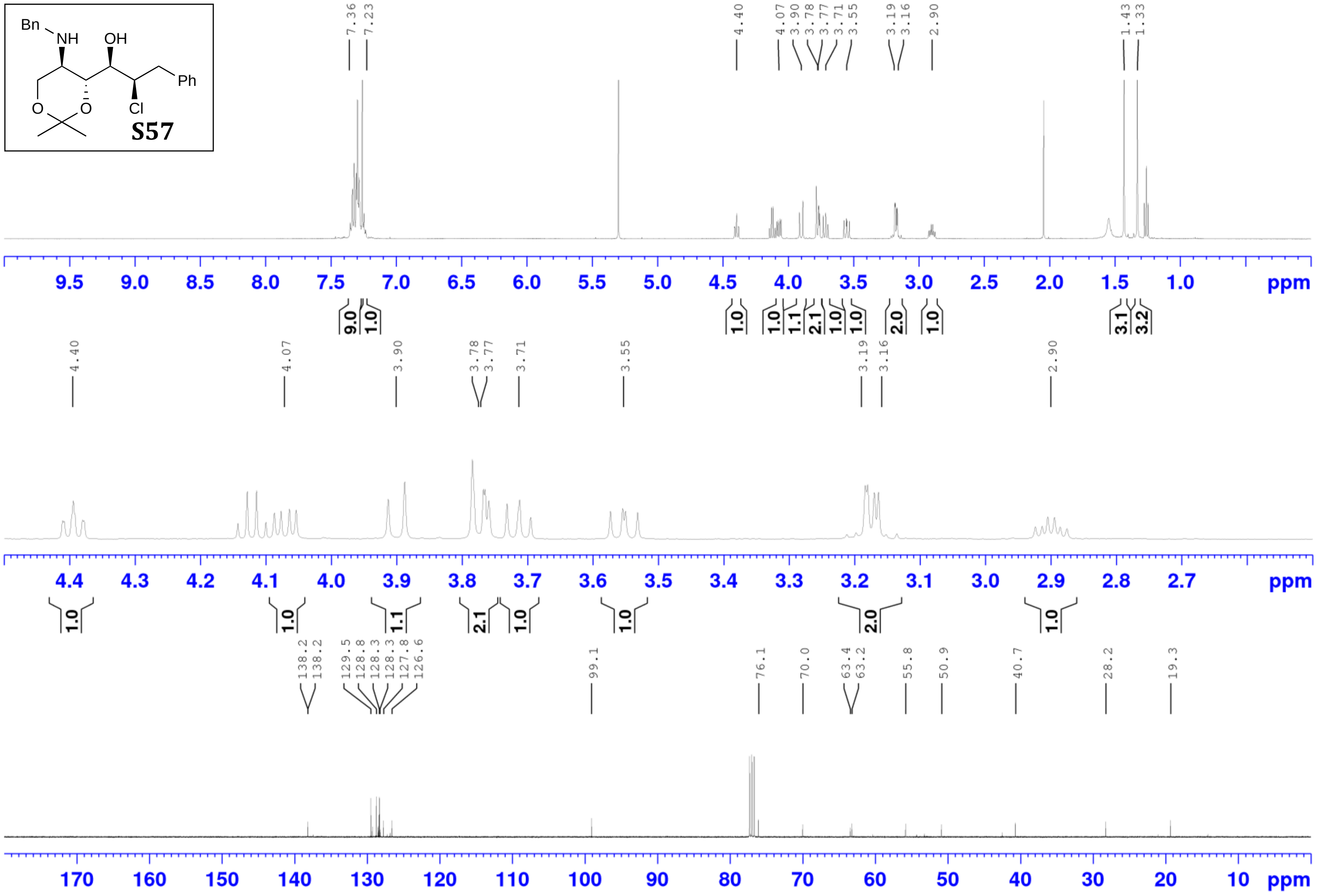

Supplementary Figure 18. ¹H and ¹³C NMR of compound S57

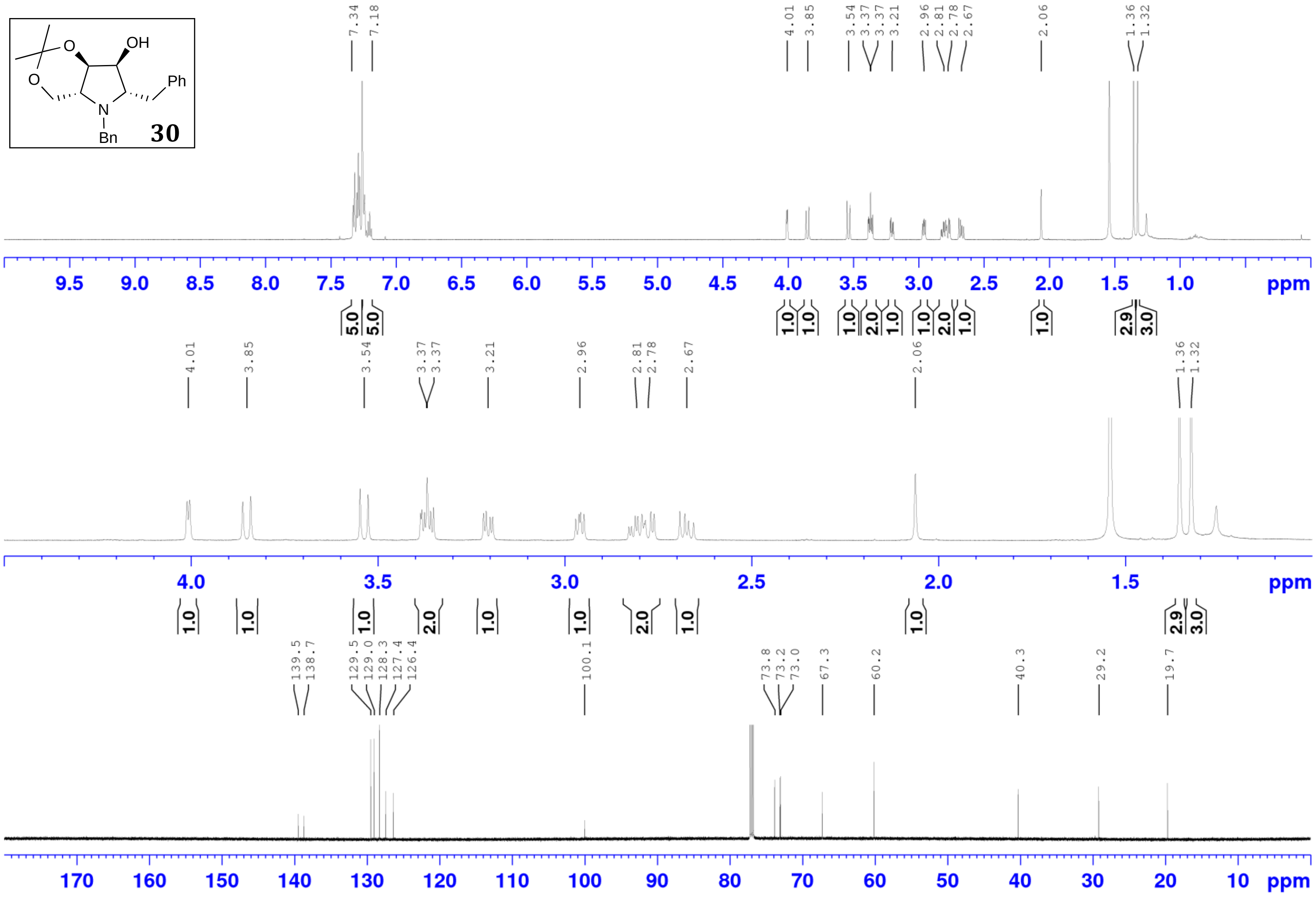

Supplementary Figure 19. ^{1}H and ^{13}C NMR of compound 30

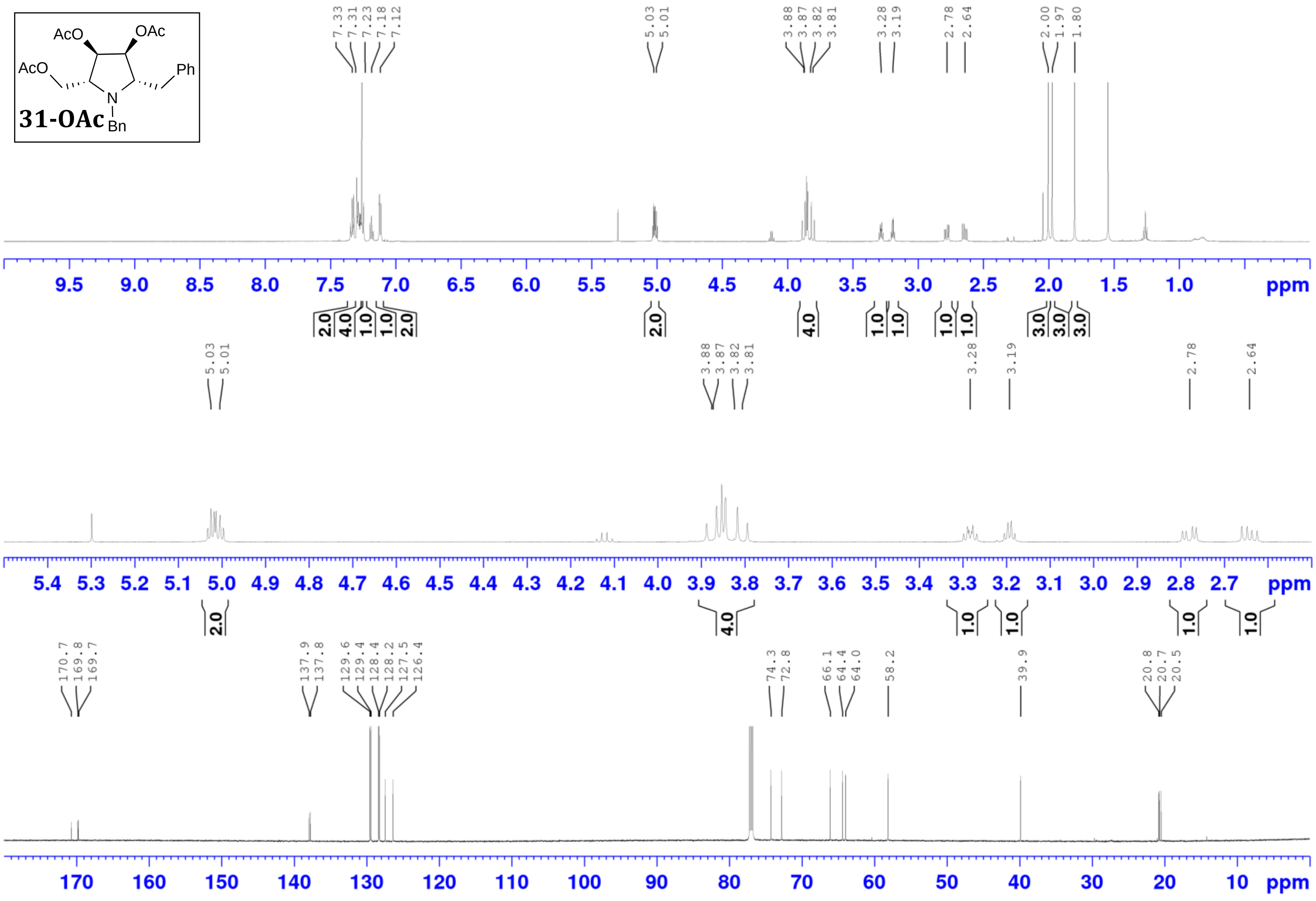

Supplementary Figure 20. 1H and 13C NMR of compound 31-OAc

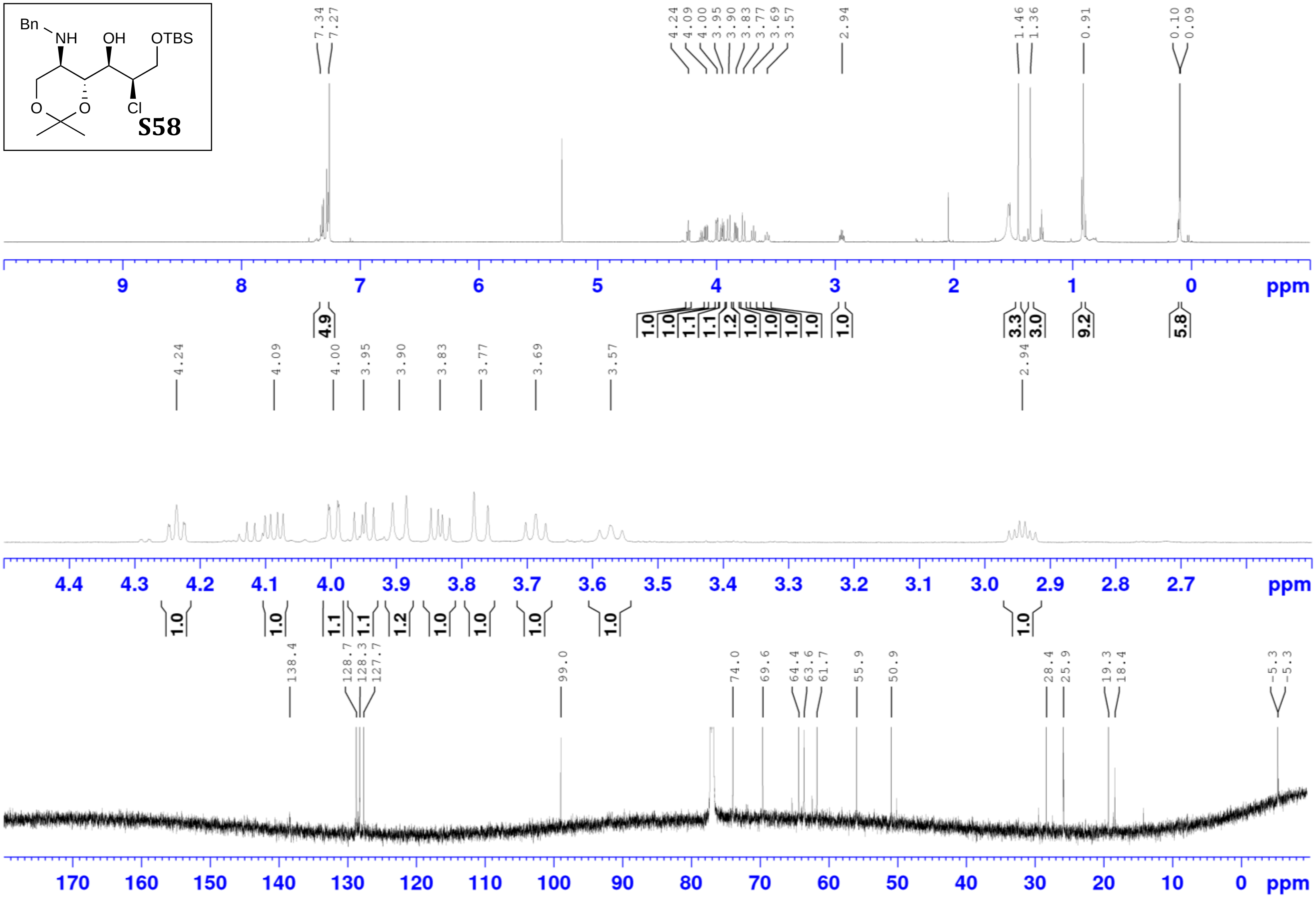

Supplementary Figure 21. ^{1}H and ^{13}C NMR of compound S58

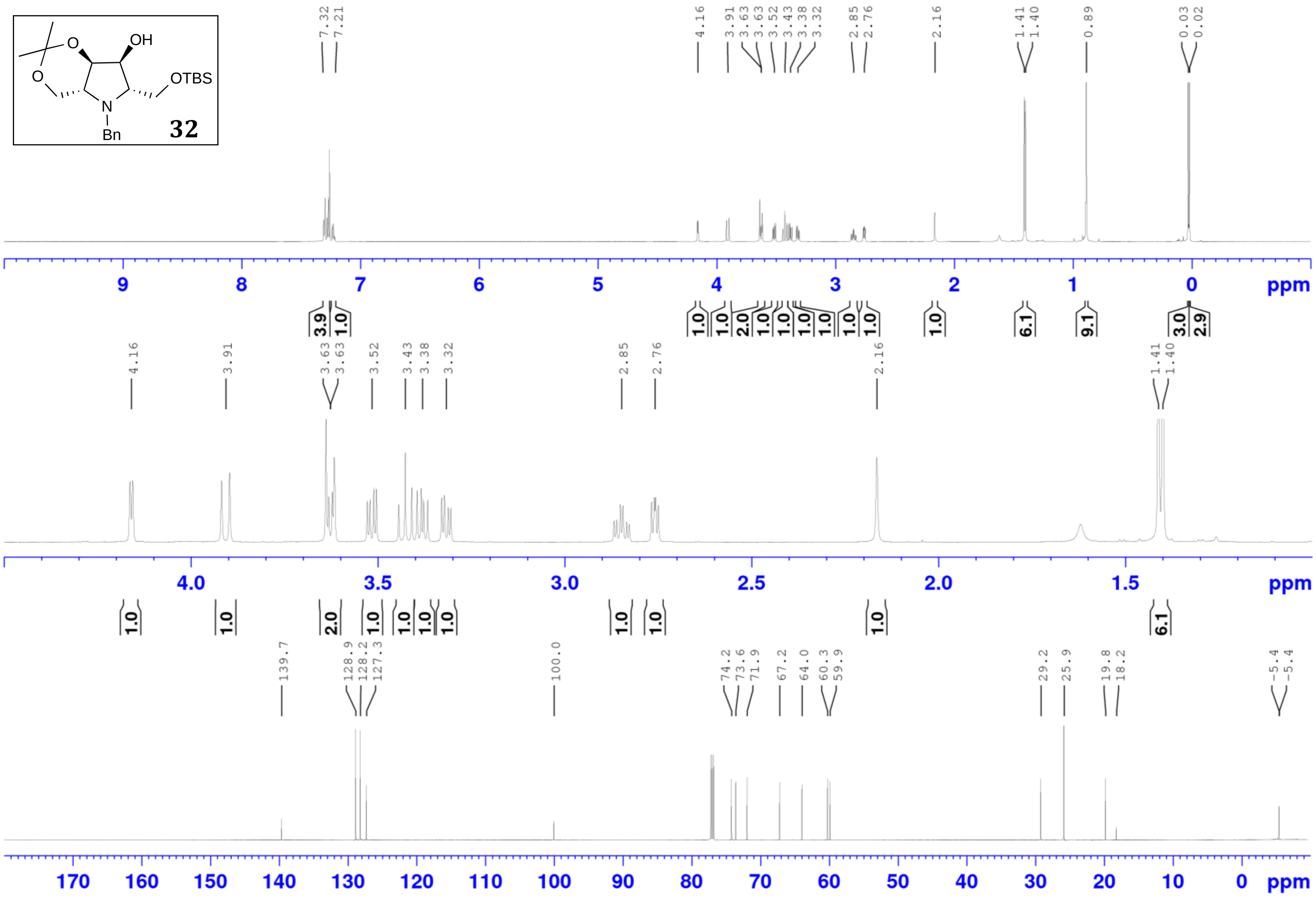

Supplementary Figure 22. ^{1}H **and** ^{13}C **NMR of compound 32**

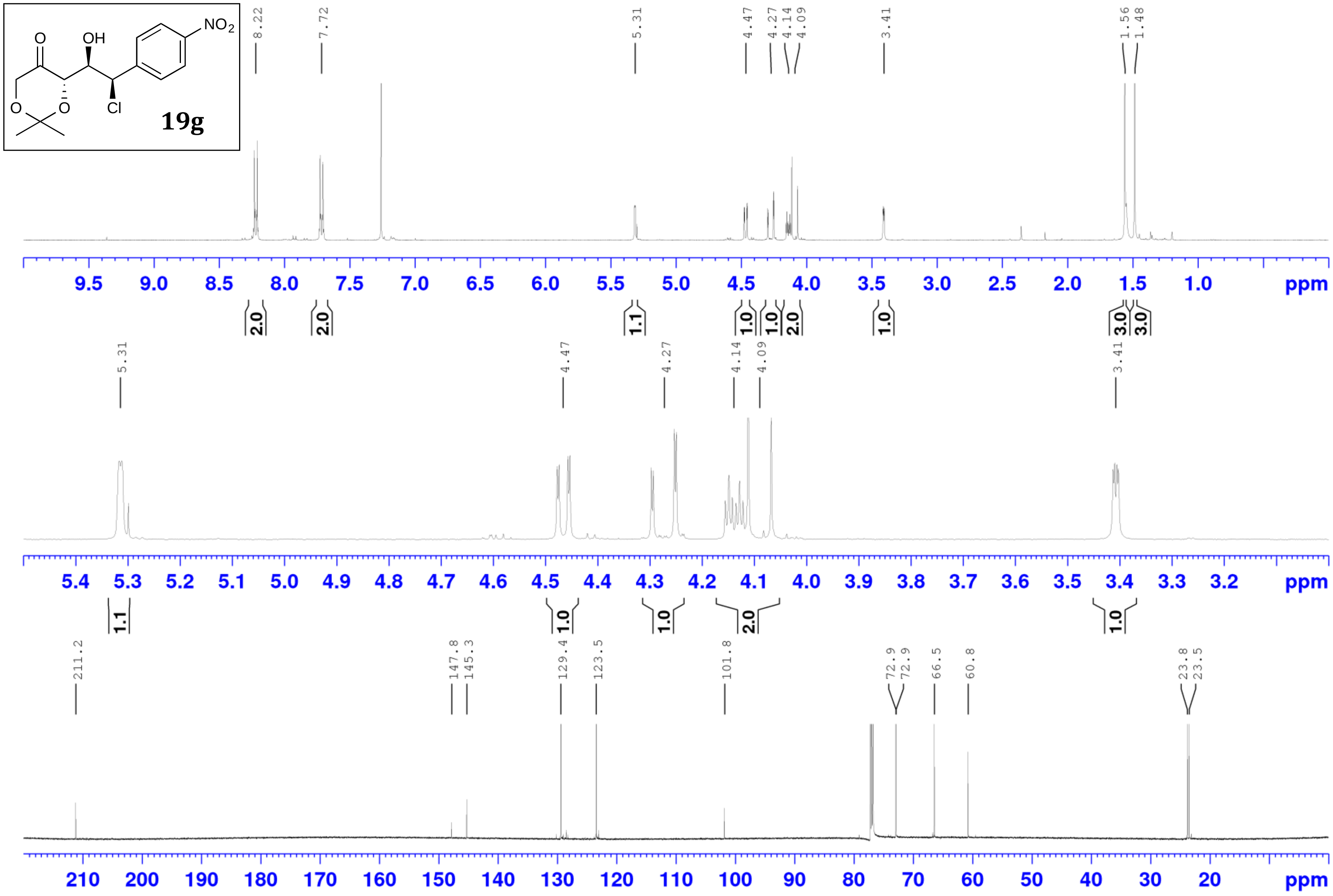

Supplementary Figure 23. ^{1}**H and** ^{13}**C NMR of compound 19g**

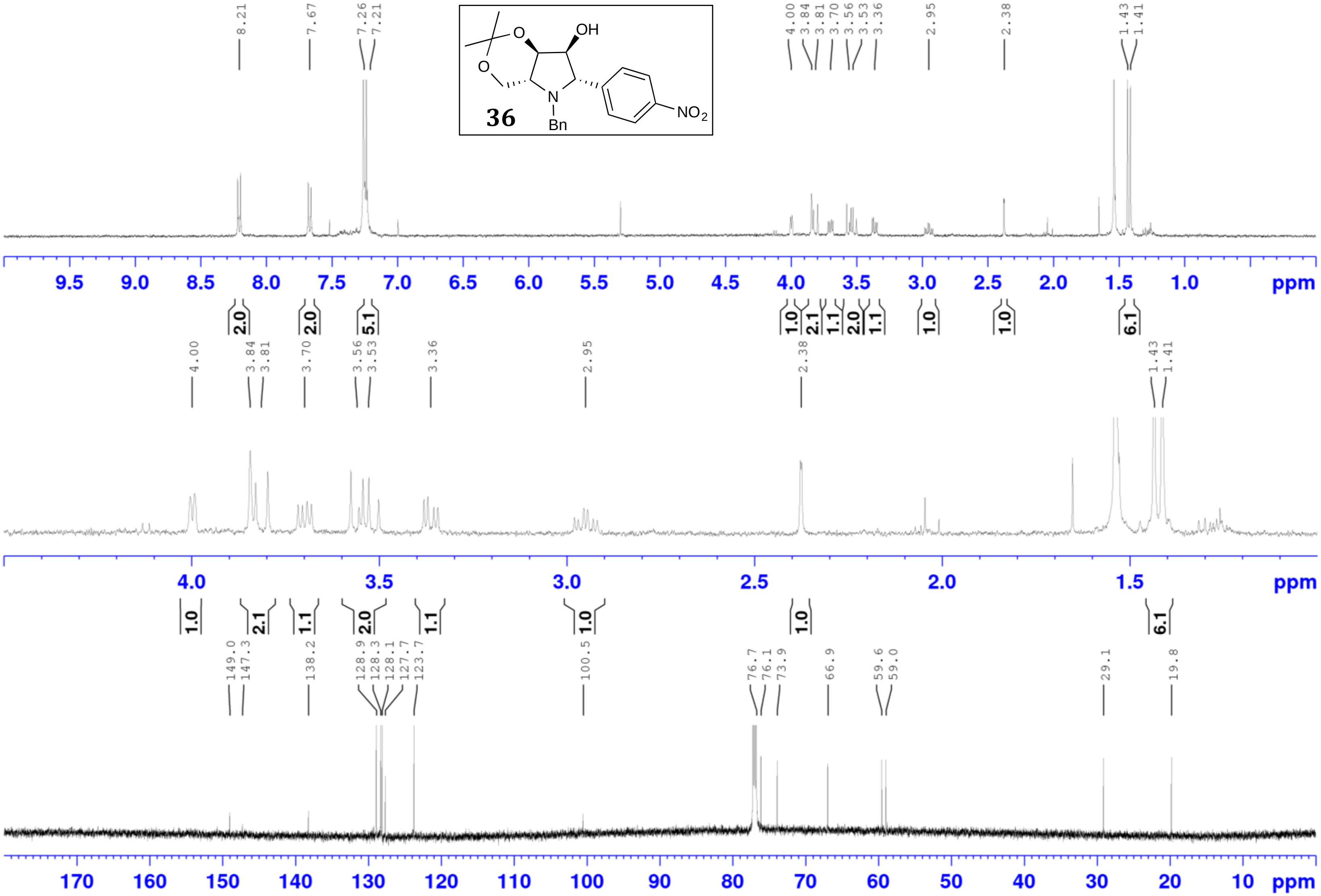

Supplementary Figure 24. ^{1}**H and** ^{13}**C NMR of compound 36**

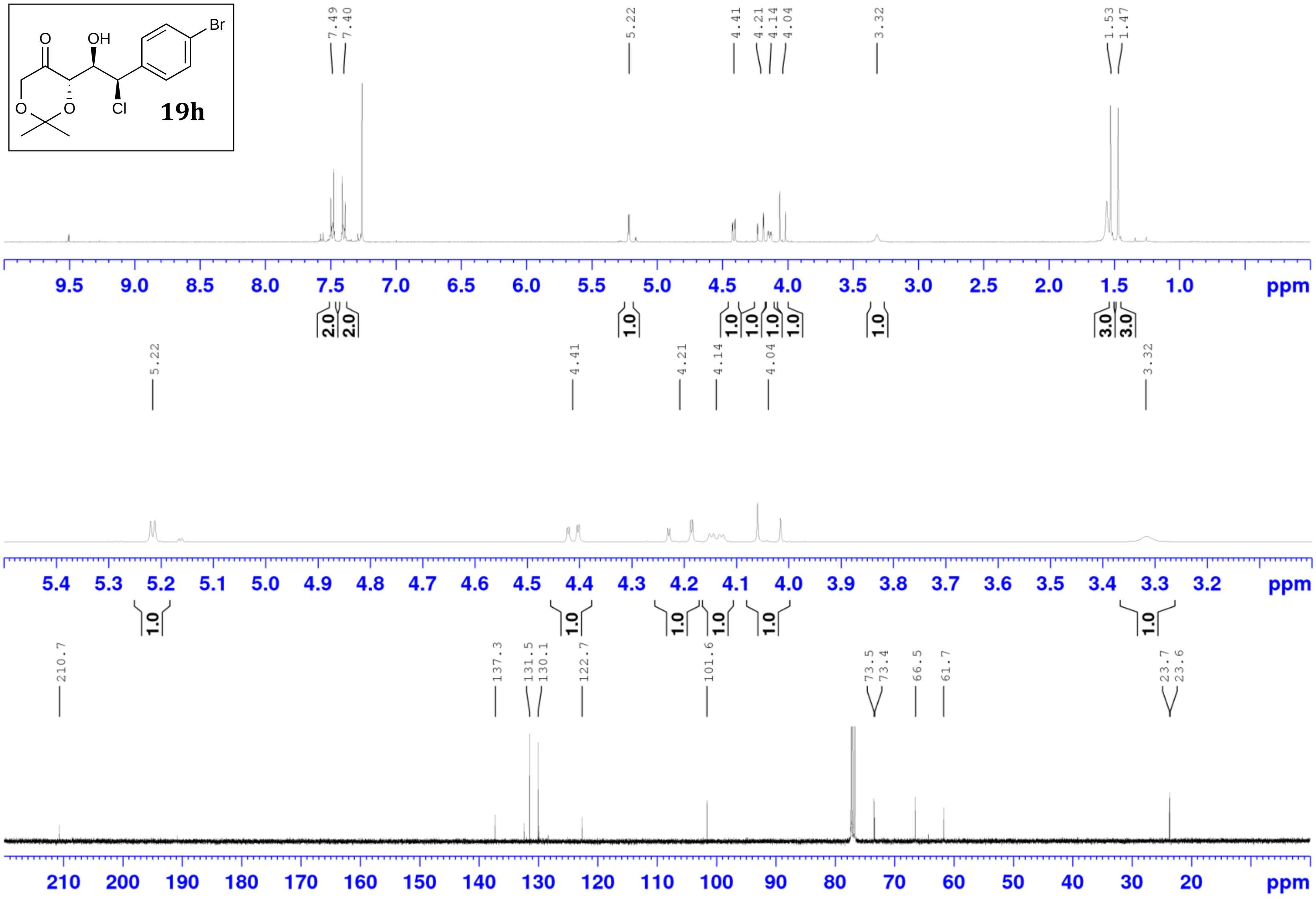

Supplementary Figure 25. ^{1}H and ^{13}C NMR of compound 19h

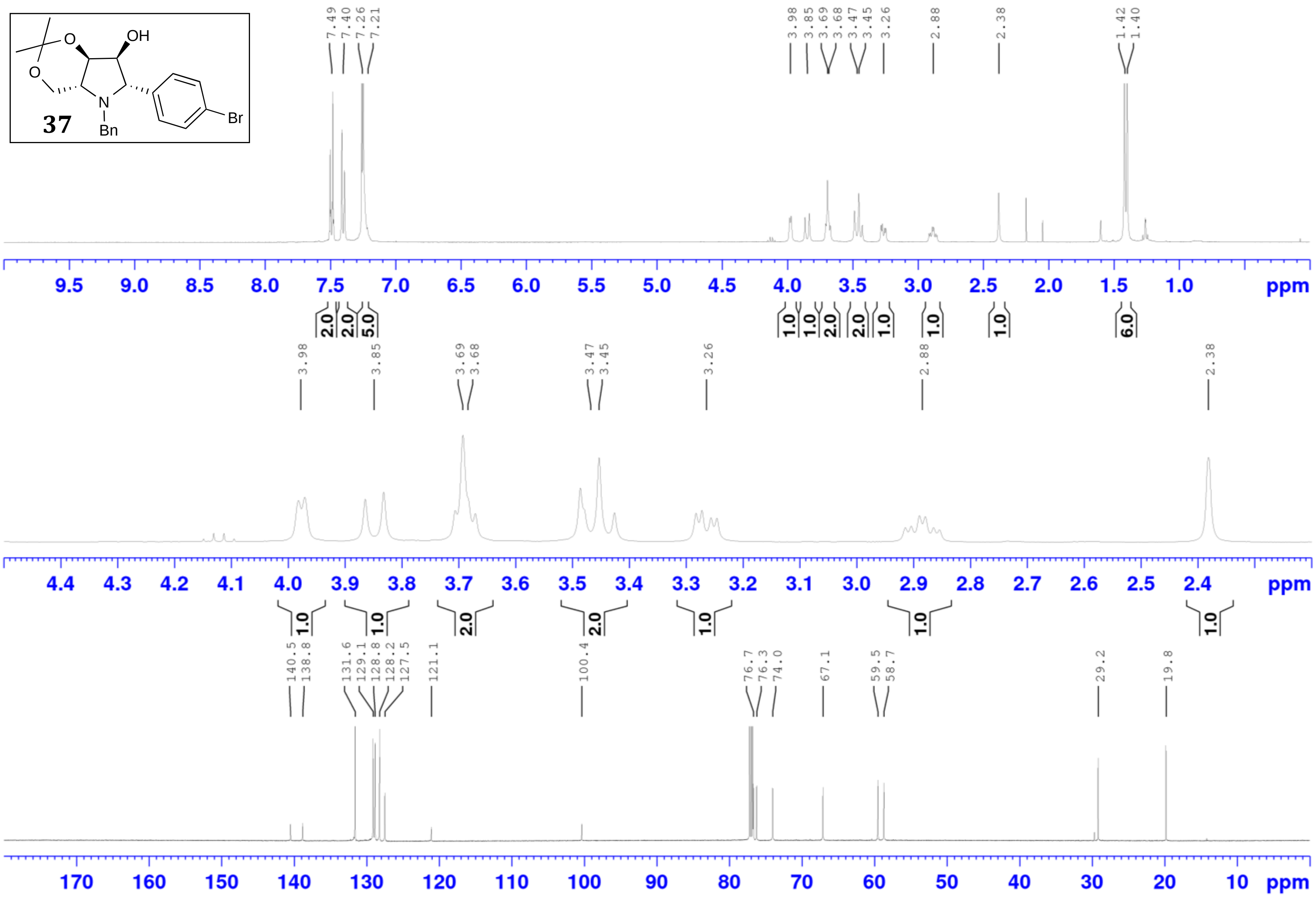

Supplementary Figure 26. ^{1}H and ^{13}C NMR of compound 37

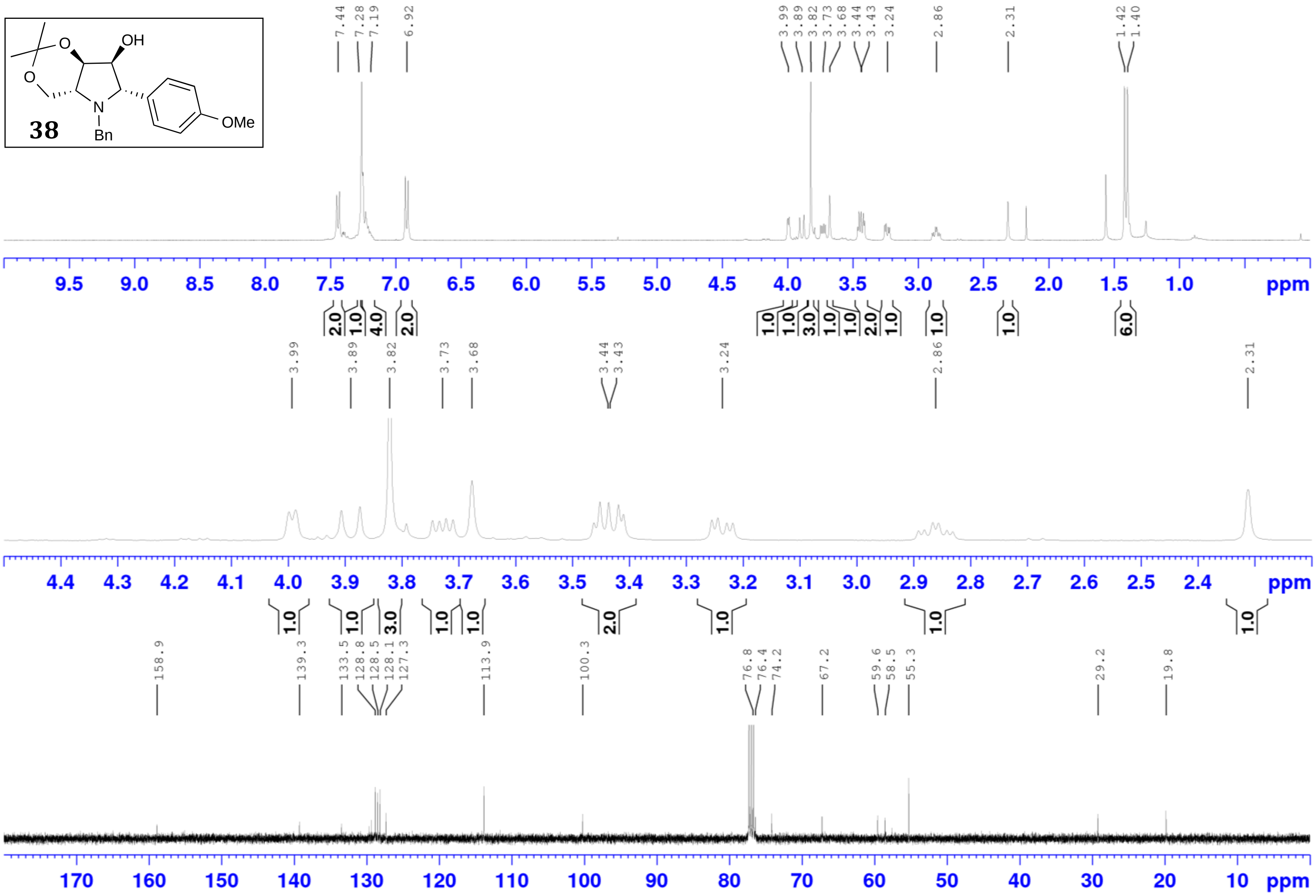

Supplementary Figure 27. ^{1}H and ^{13}C NMR of compound 38

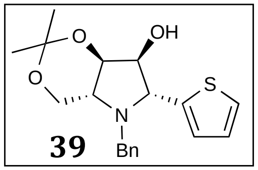

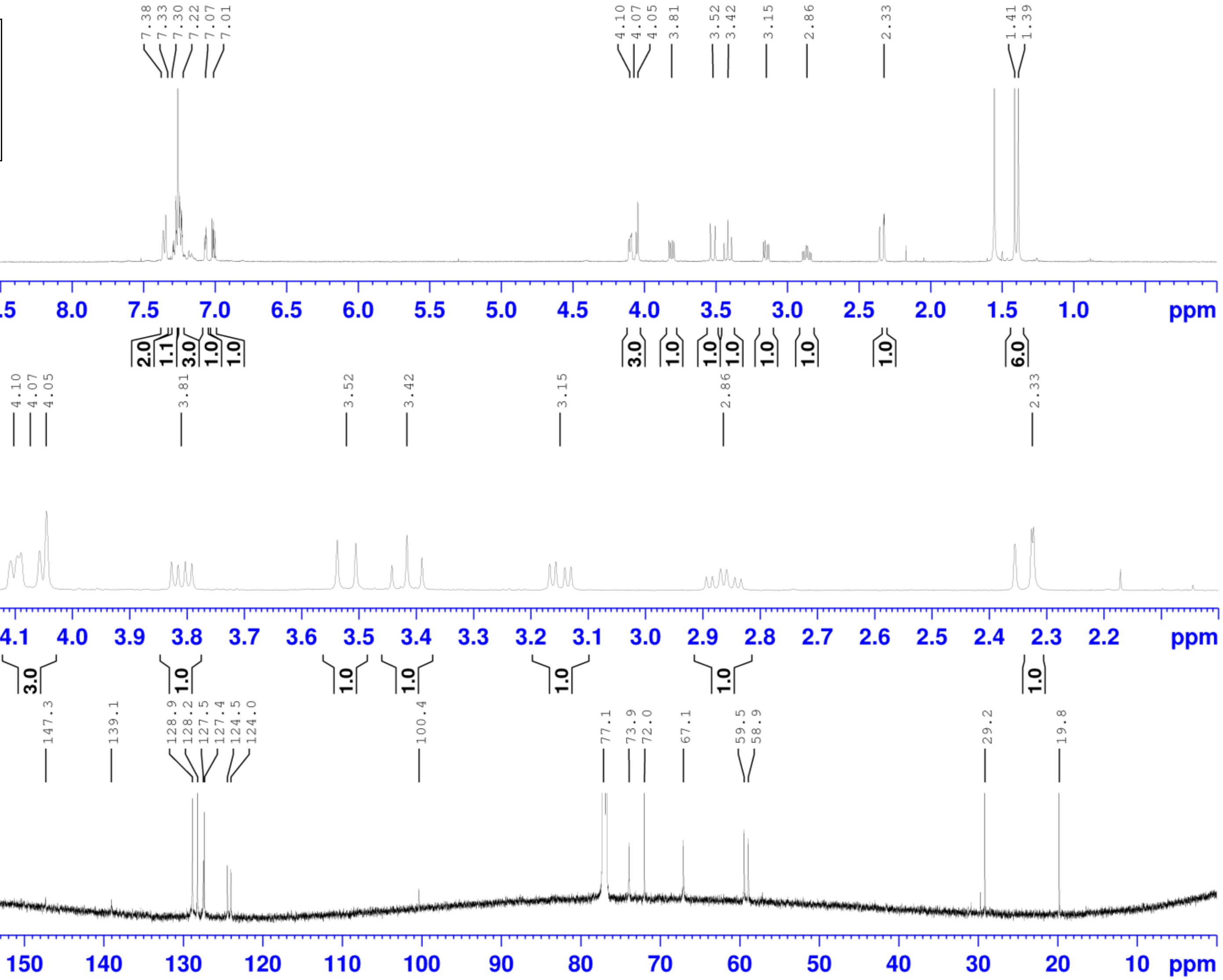

Supplementary Figure 28. ^{1}H and ^{13}C NMR of compound 39

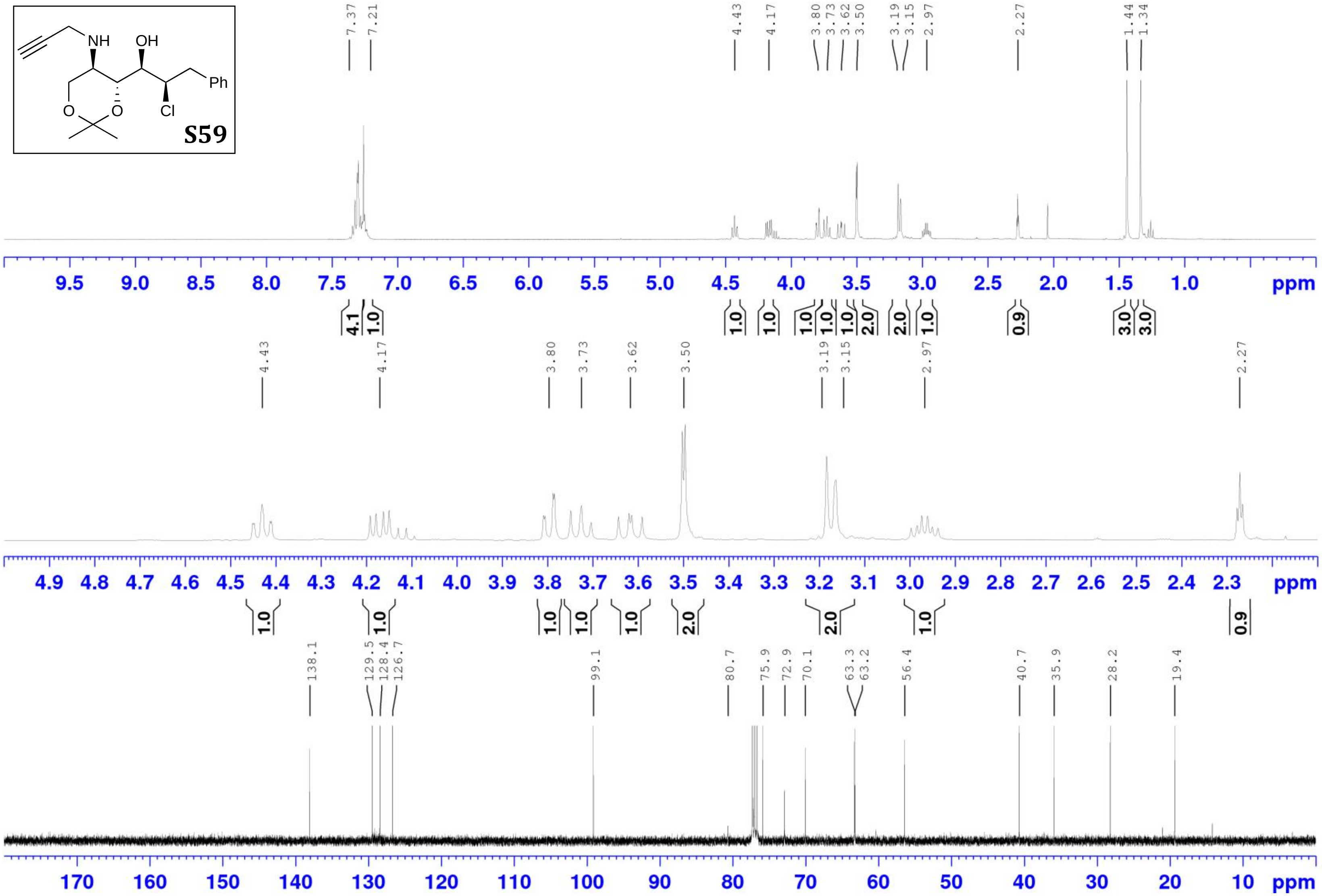

Supplementary Figure 29. ^{1}H and ^{13}C NMR of compound S59

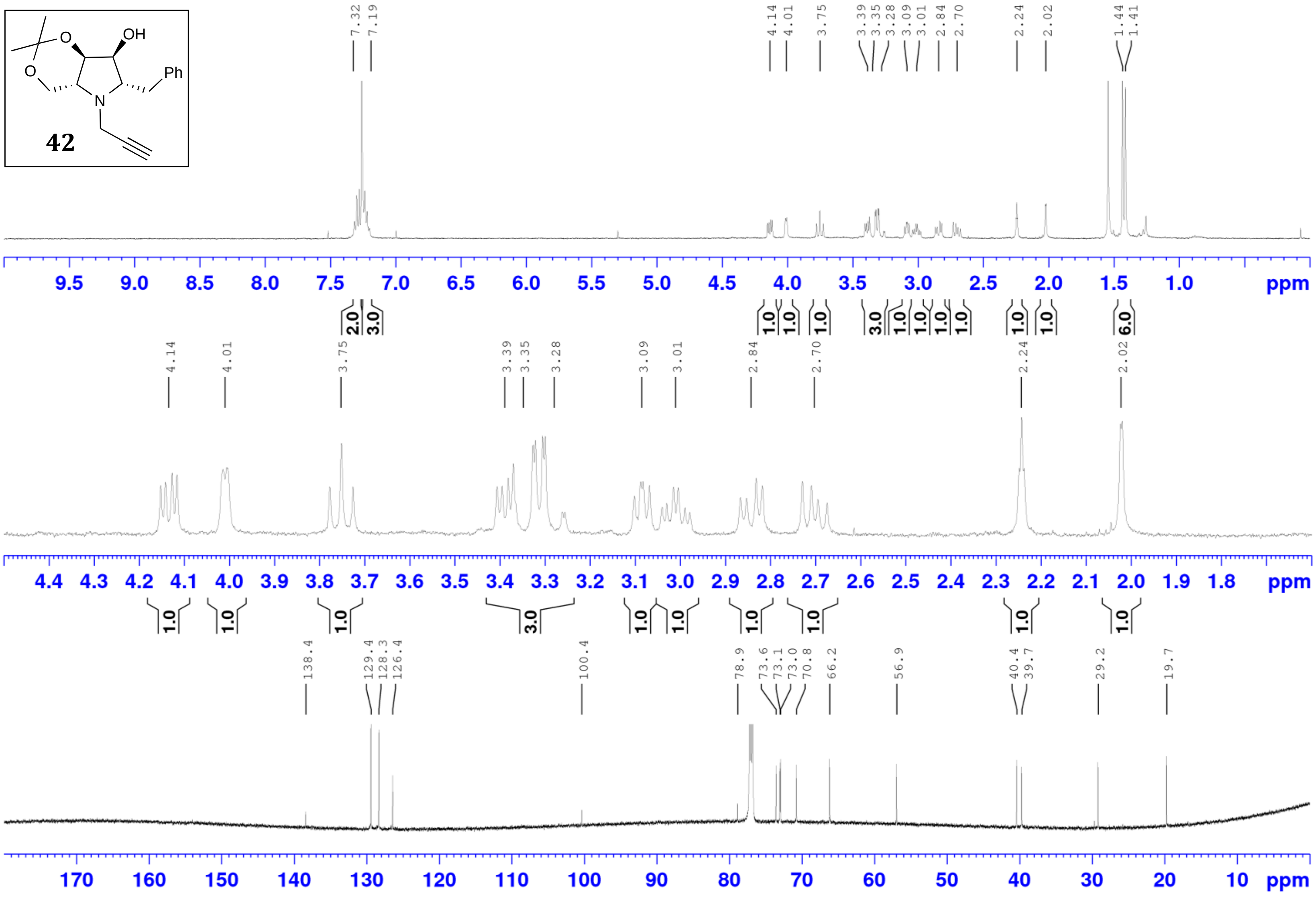

Supplementary Figure 30. ^{1}H and ^{13}C NMR of compound 42

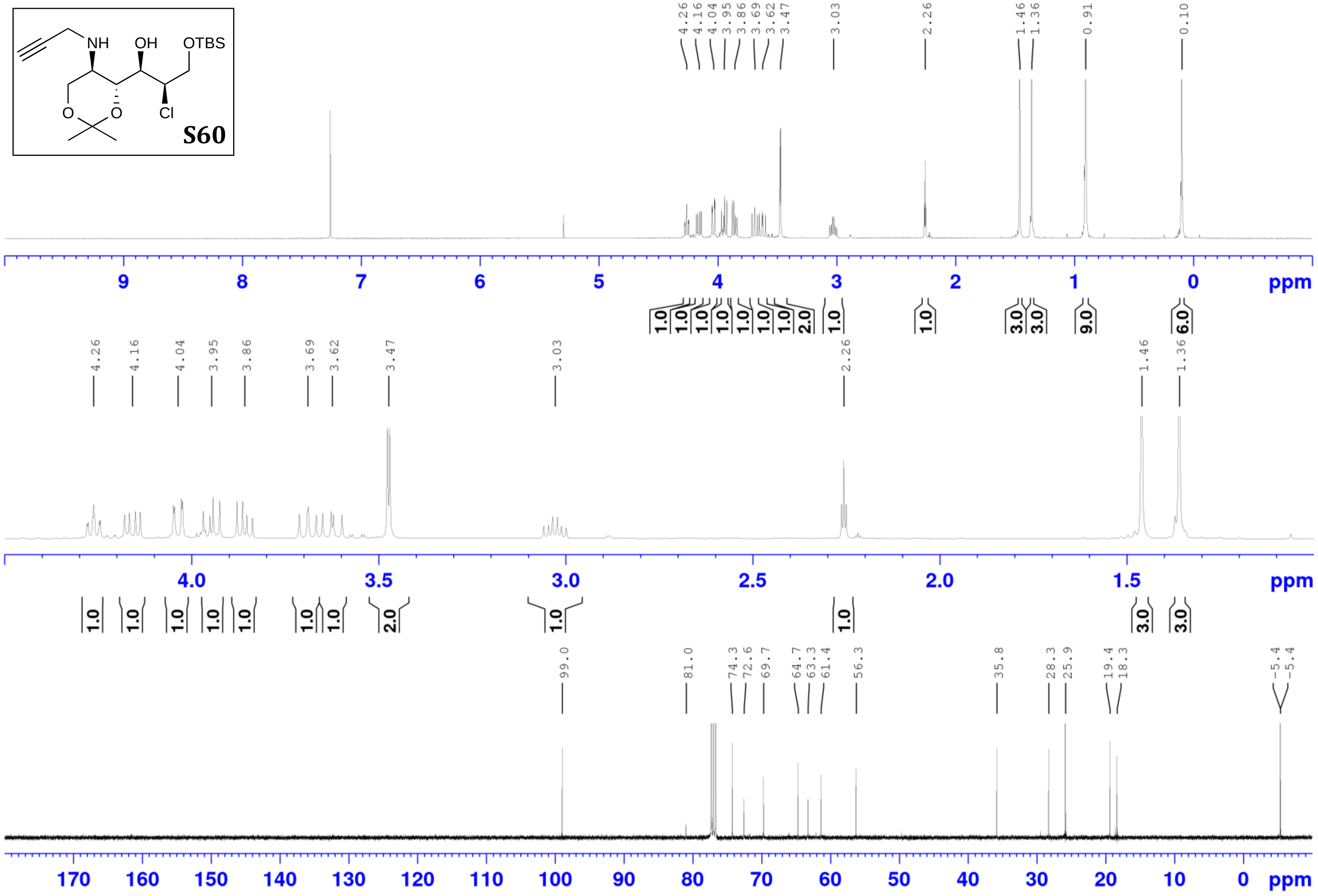

Supplementary Figure 31. ^{1}H and ^{13}C NMR of compound S60

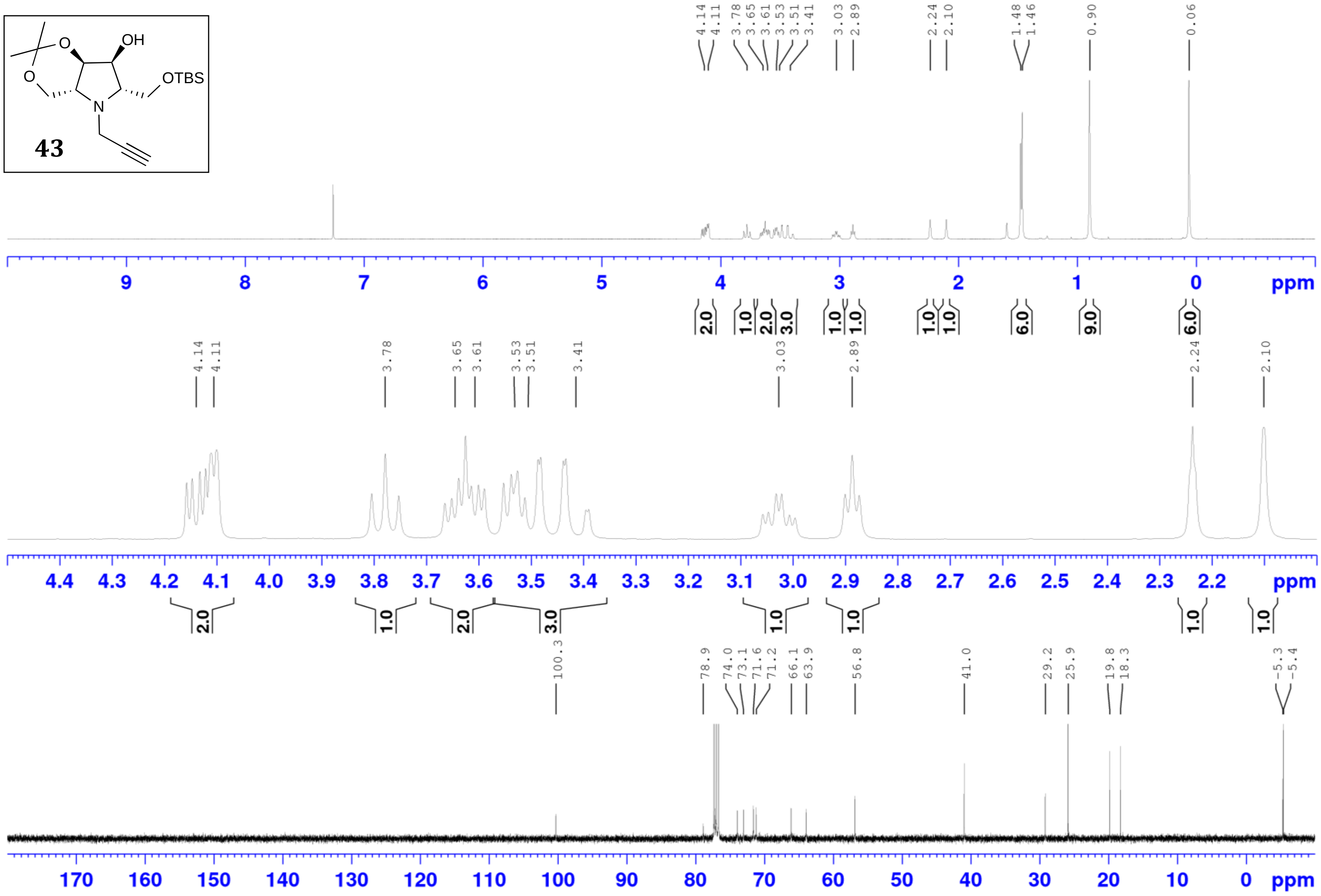

Supplementary Figure 32. ^{1}H and ^{13}C NMR of compound 43

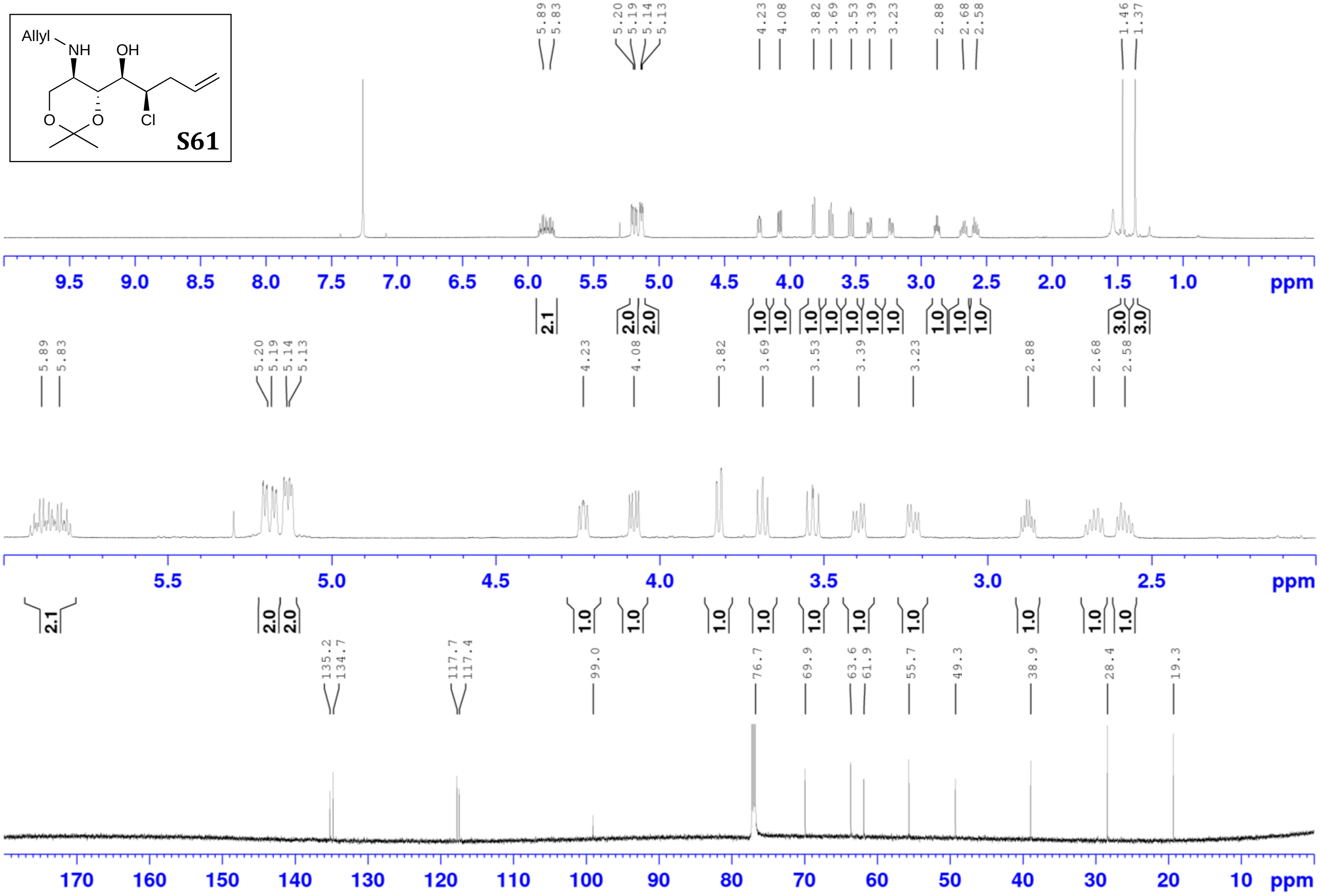

Supplementary Figure 33. ^{1}H and ^{13}C NMR of compound S61

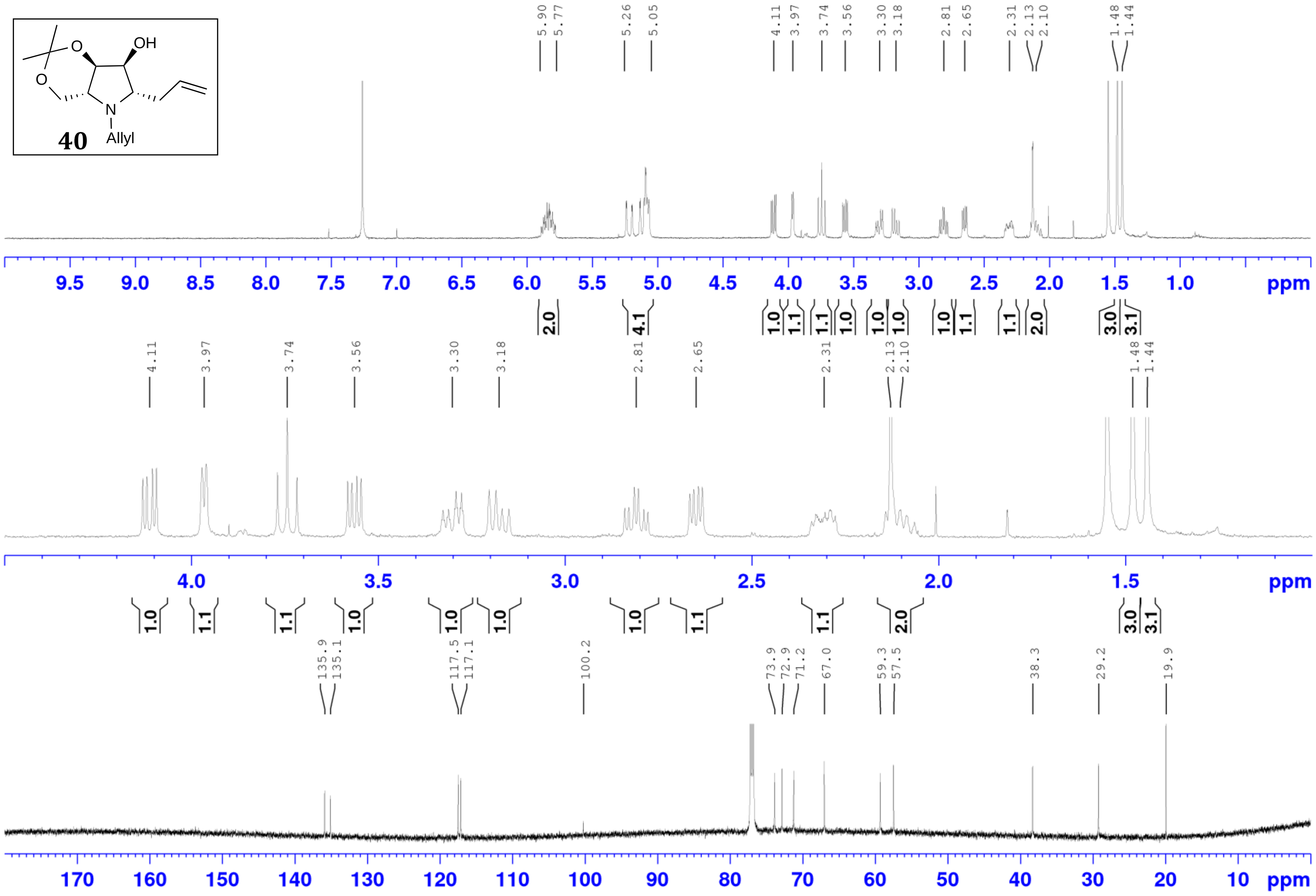

Supplementary Figure 34. ^{1}H **and** ^{13}C **NMR of compound 40**

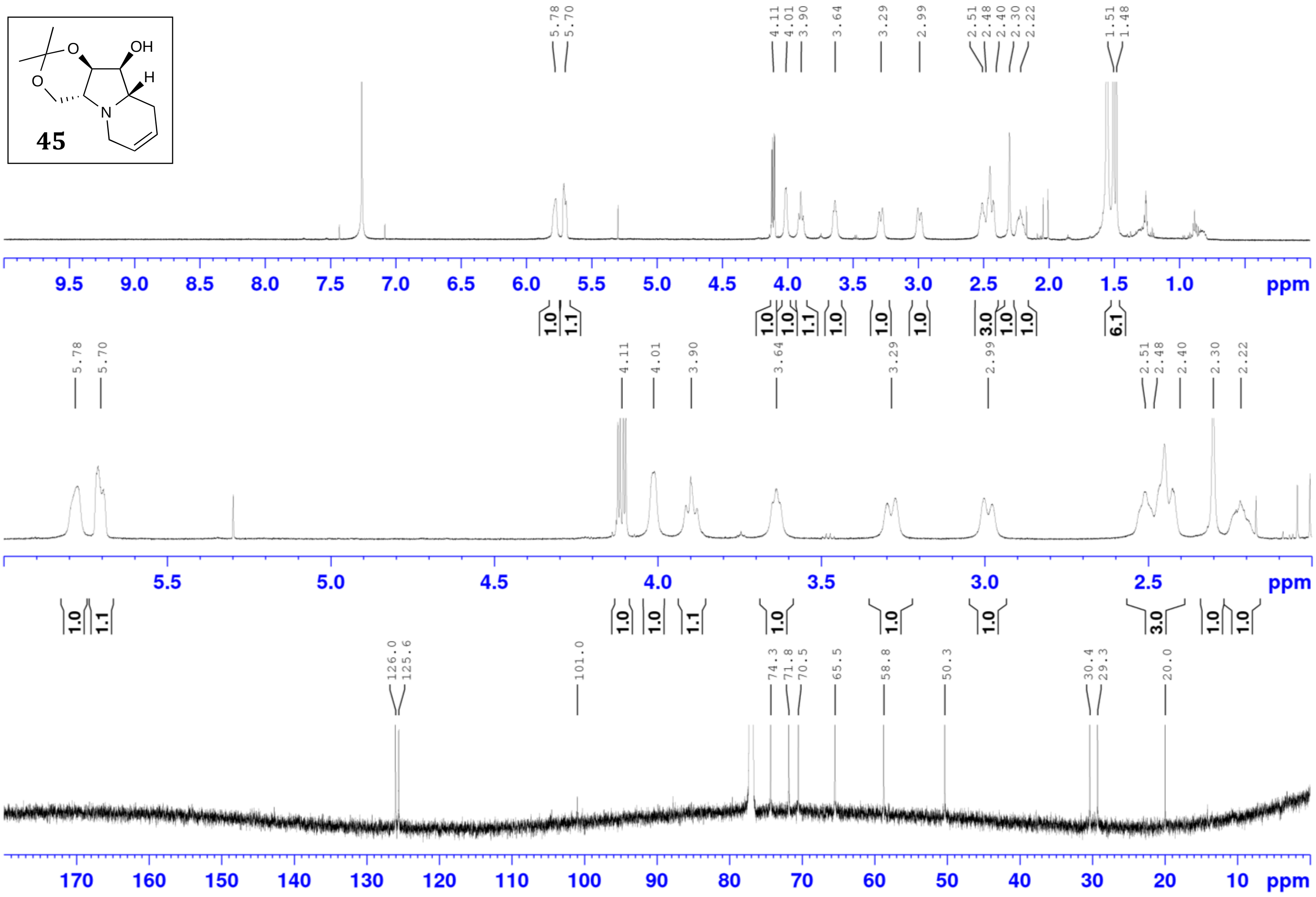

Supplementary Figure 35. ^{1}H and ^{13}C NMR of compound 45

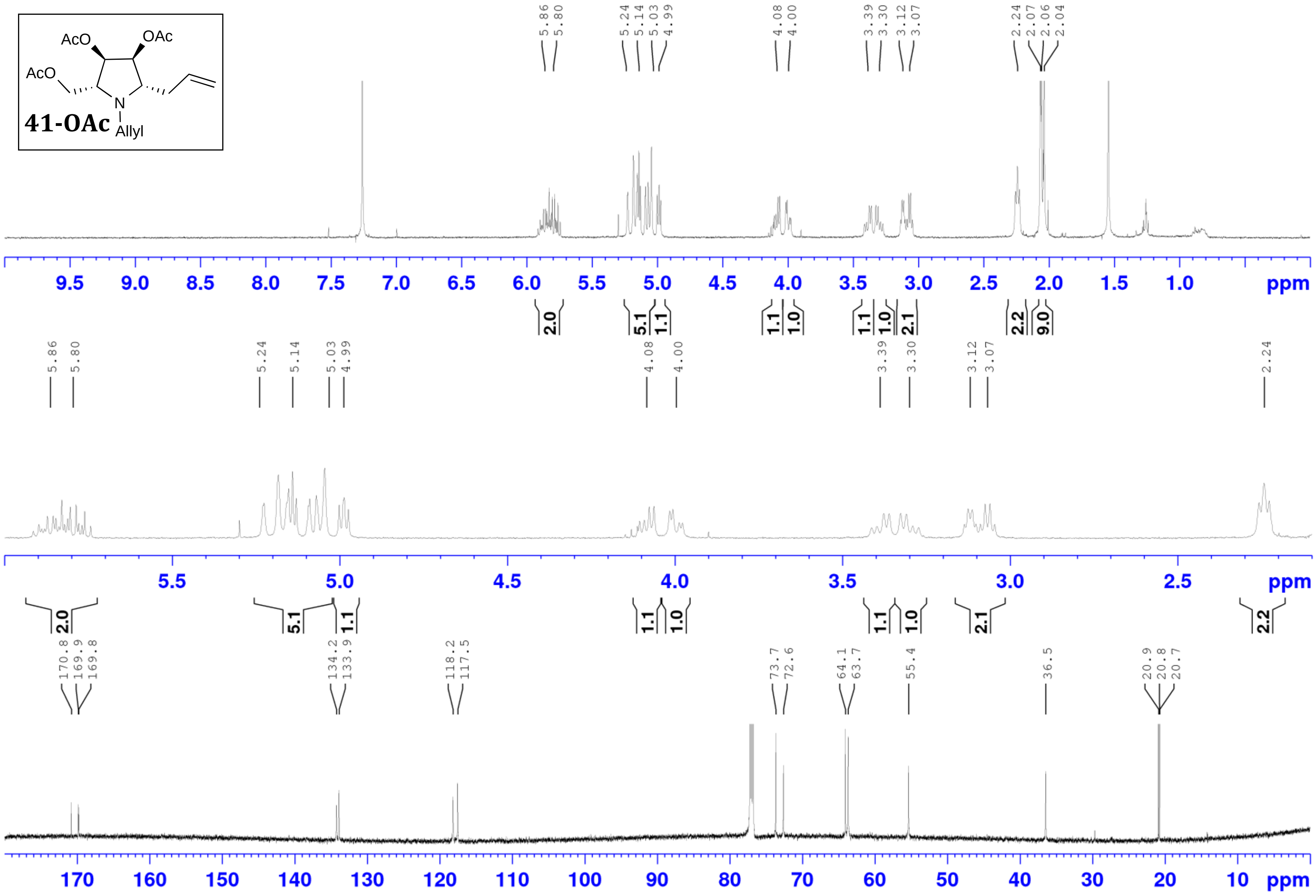

Supplementary Figure 36. ^{1}H and ^{13}C NMR of compound 41-OAc

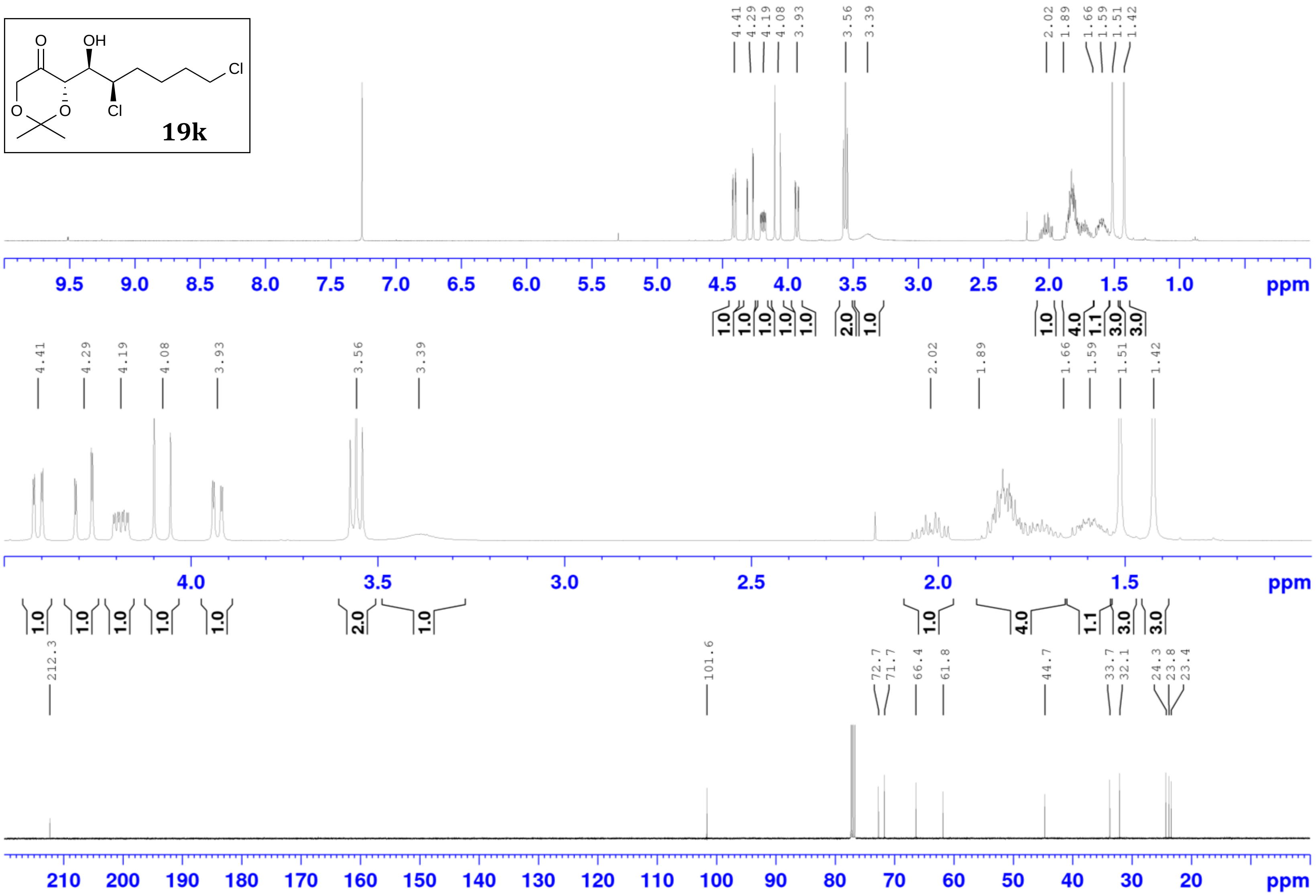

Supplementary Figure 37. ^{1}H and ^{13}C NMR of compound 19k

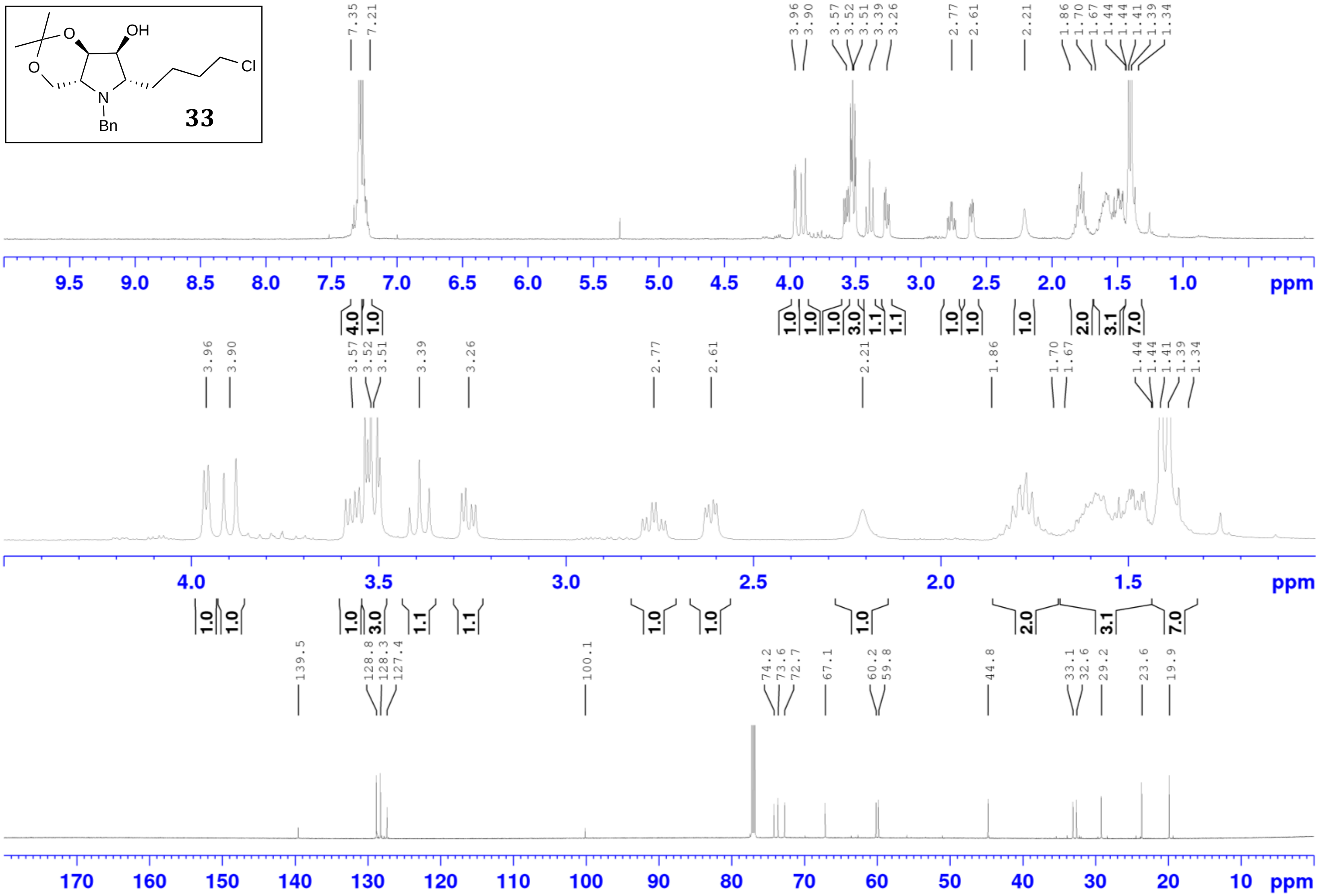

Supplementary Figure 38. ^{1}H and ^{13}C NMR of compound 33

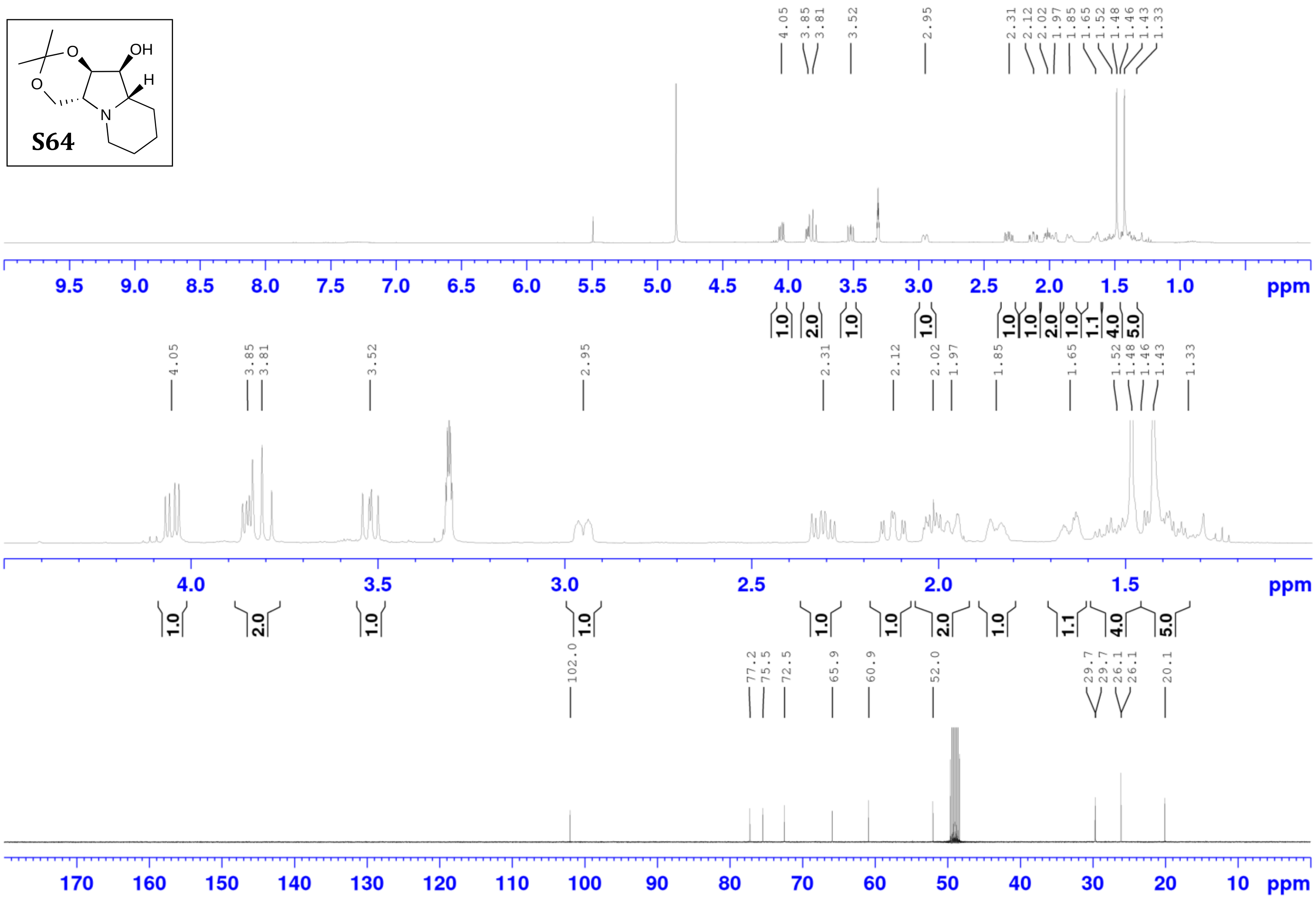

Supplementary Figure 39. ^{1}H and ^{13}C NMR of compound S64

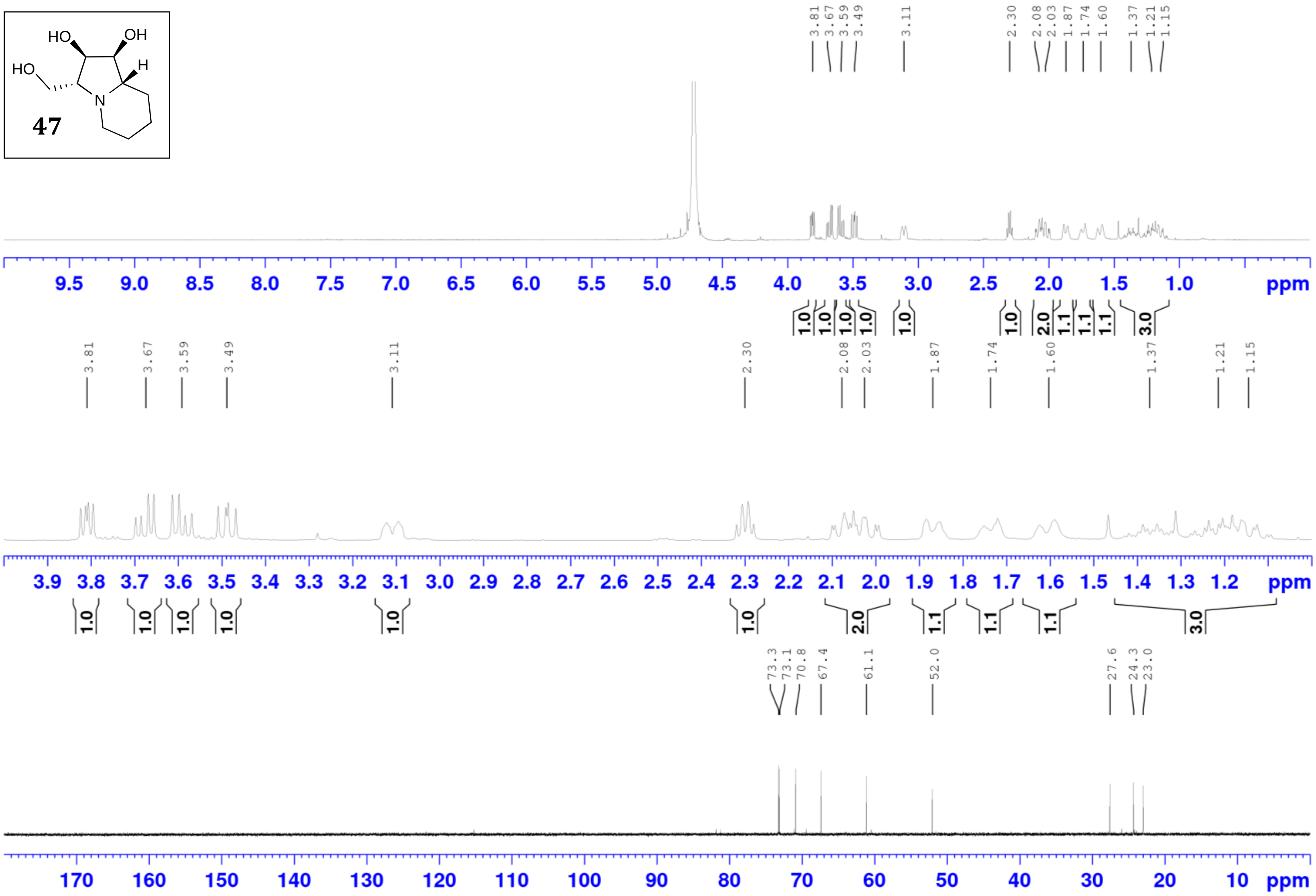

Supplementary Figure 40. ^{1}H and ^{13}C NMR of compound 47

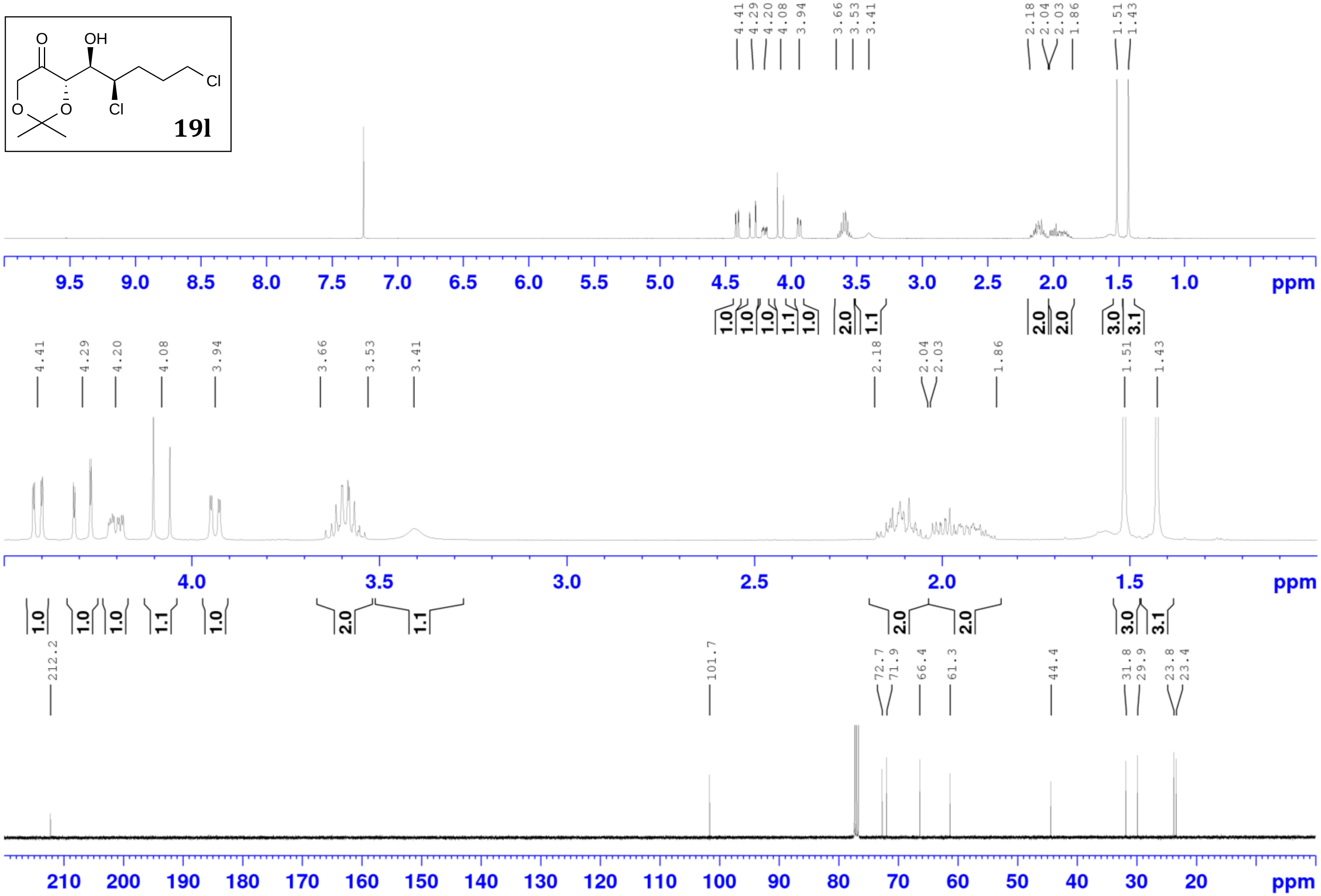

Supplementary Figure 41. ^{1}H **and** ^{13}C **NMR of compound 19l**

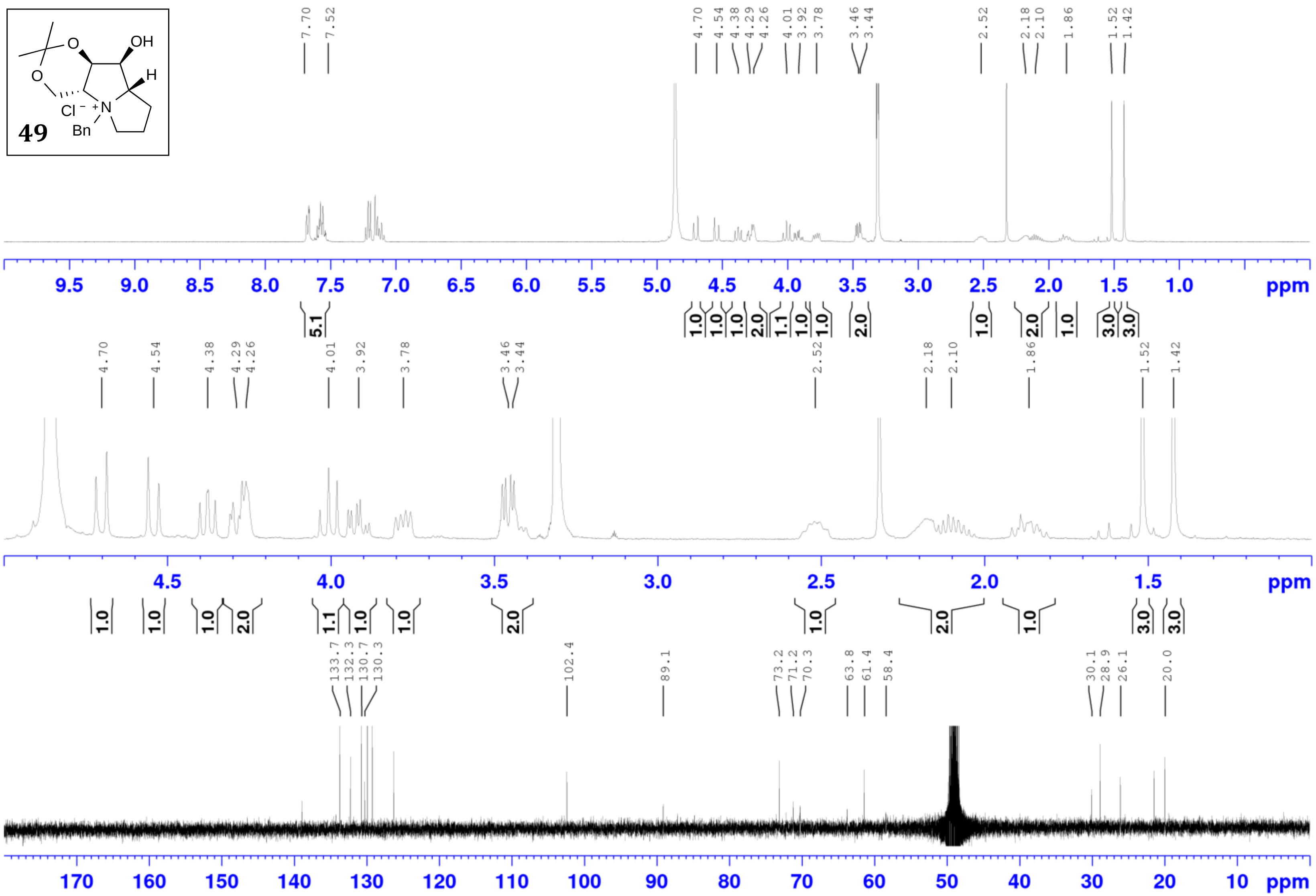

Supplementary Figure 42. ^{1}H and ^{13}C NMR of compound **49**

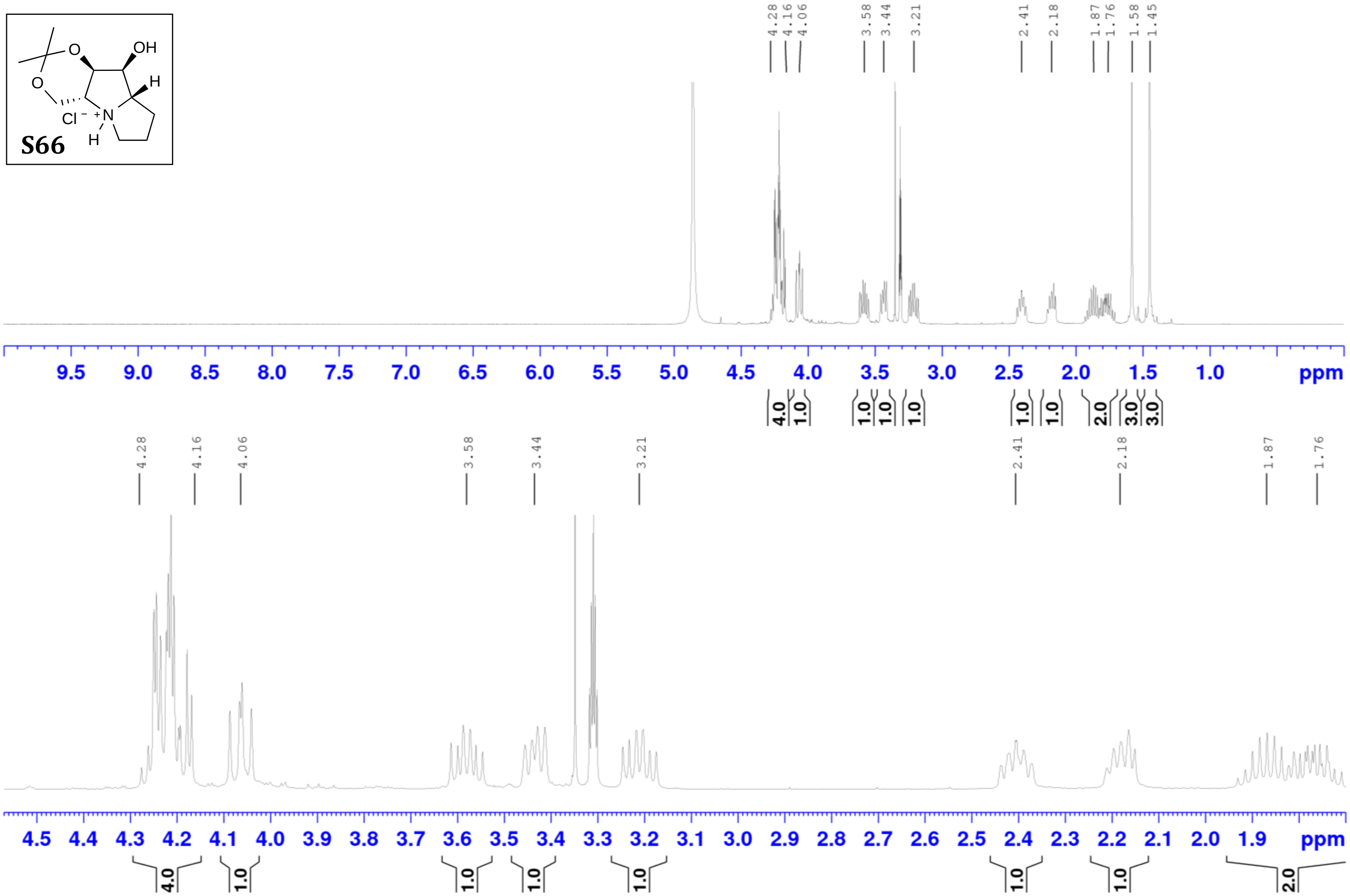

Supplementary Figure 43. ^{1}H and ^{13}C NMR of compound S66

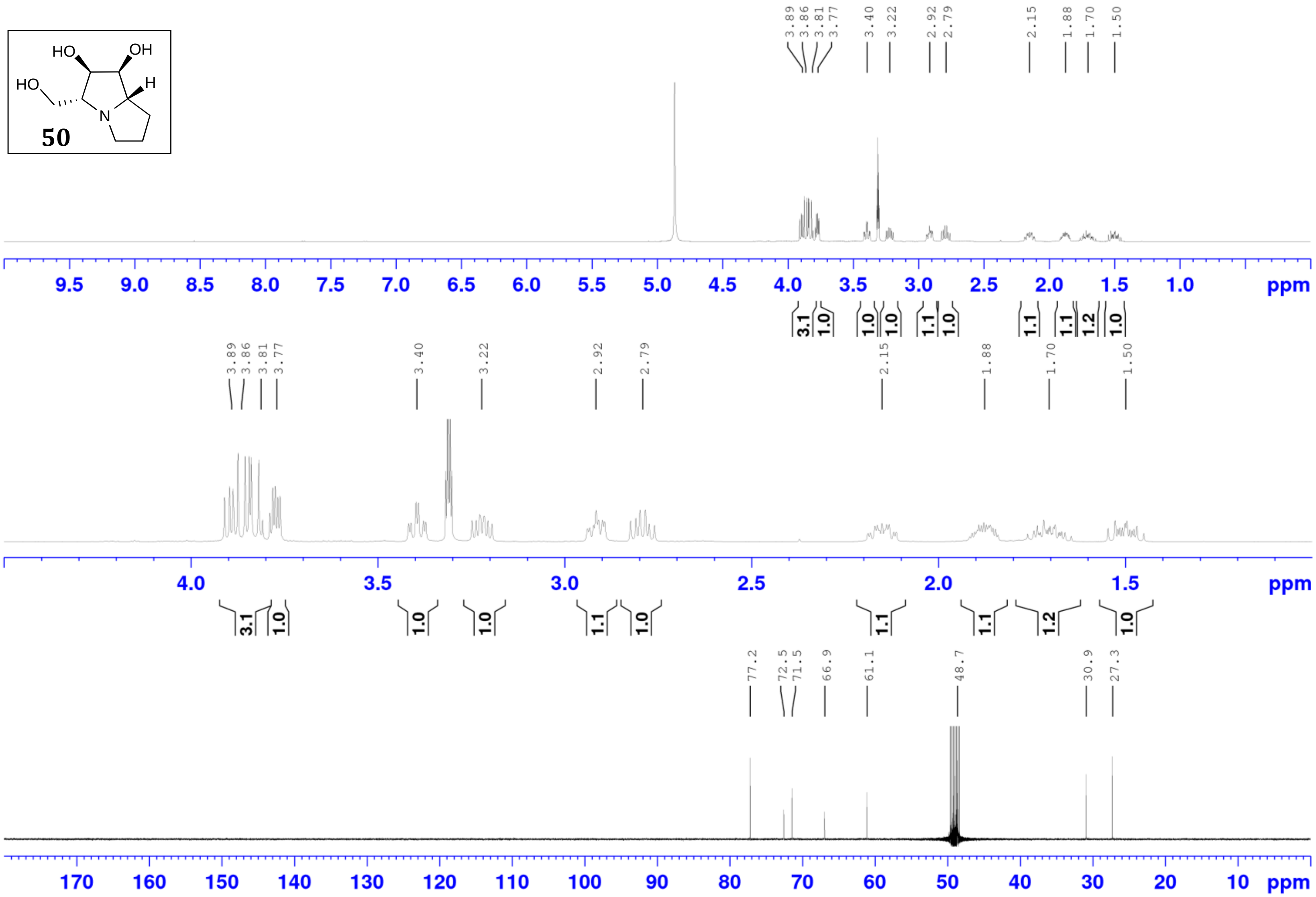

Supplementary Figure 44. ^{1}H **and** ^{13}C **NMR of compound 50**

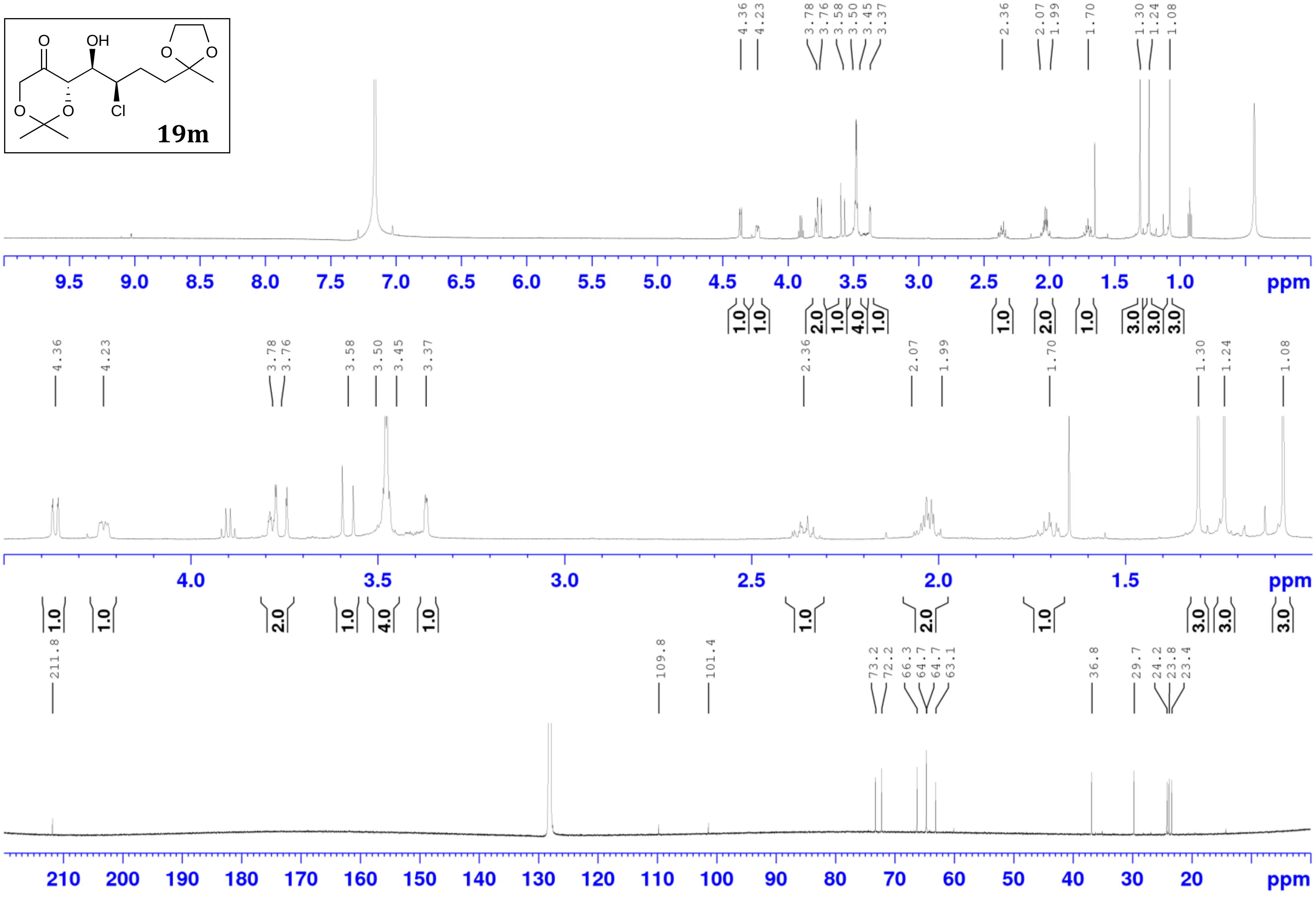

Supplementary Figure 45. ^{1}H and ^{13}C NMR of compound 19m

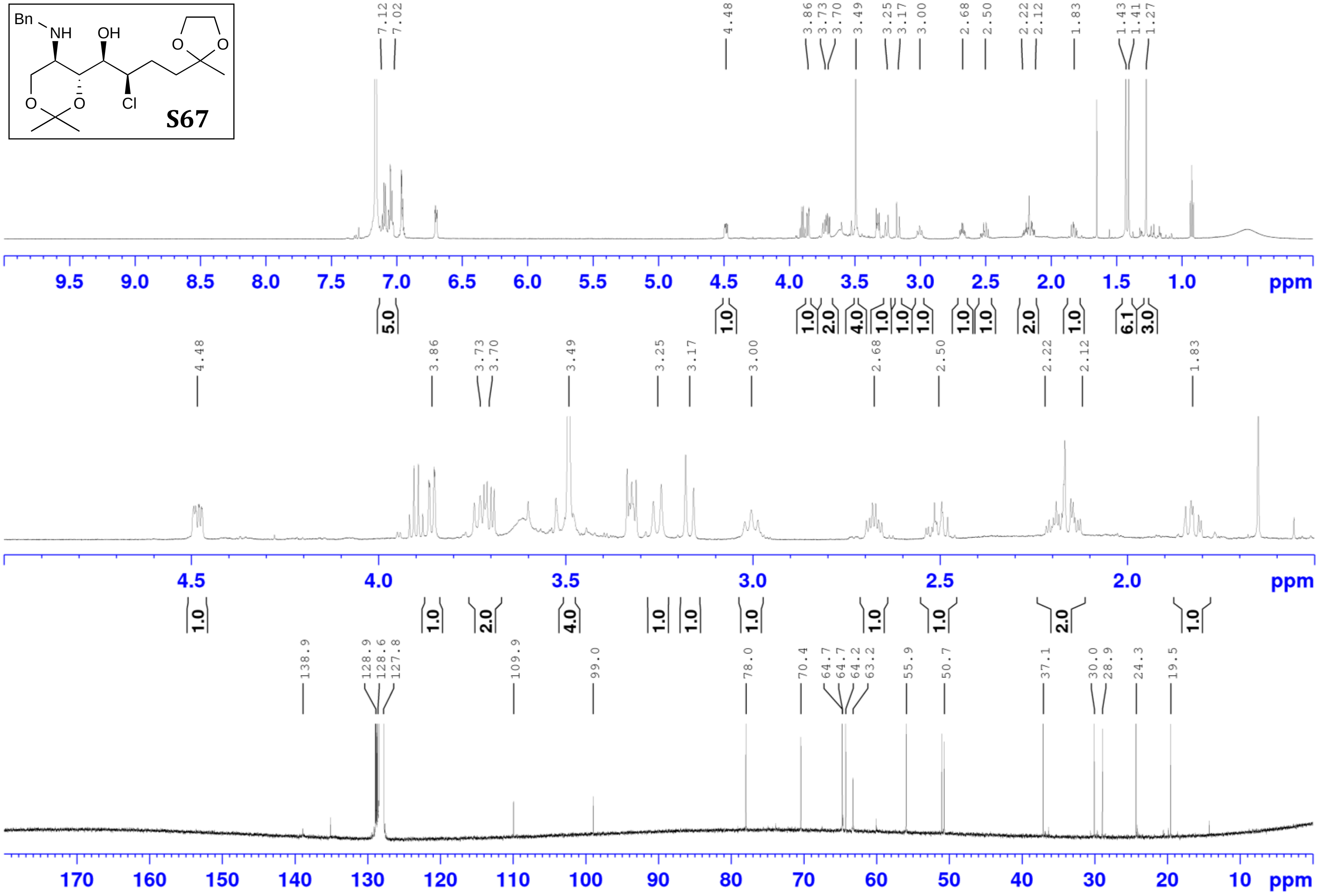

Supplementary Figure 46. ^{1}H and ^{13}C NMR of compound S67

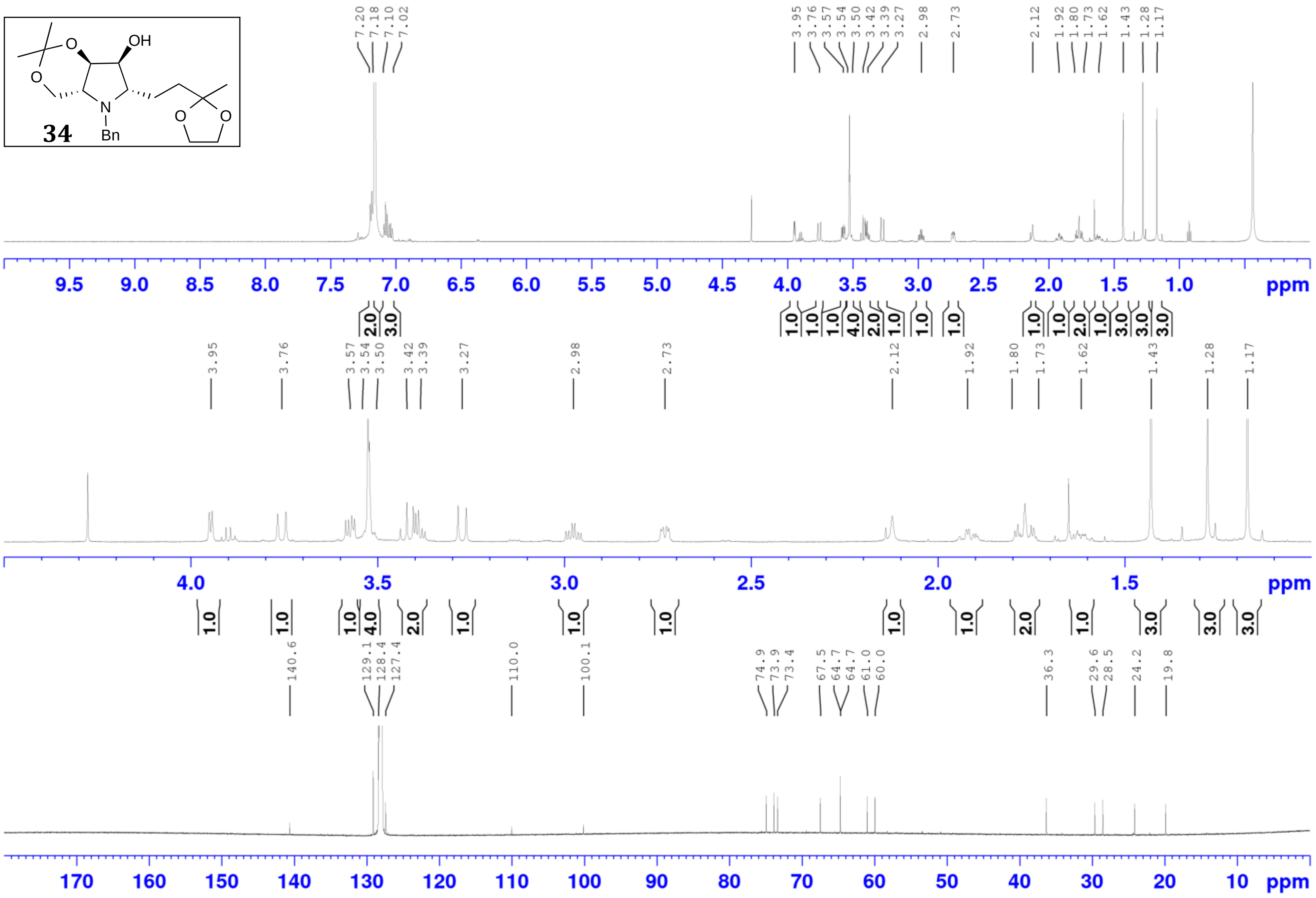

Supplementary Figure 47. ¹H and ¹³C NMR of compound 34

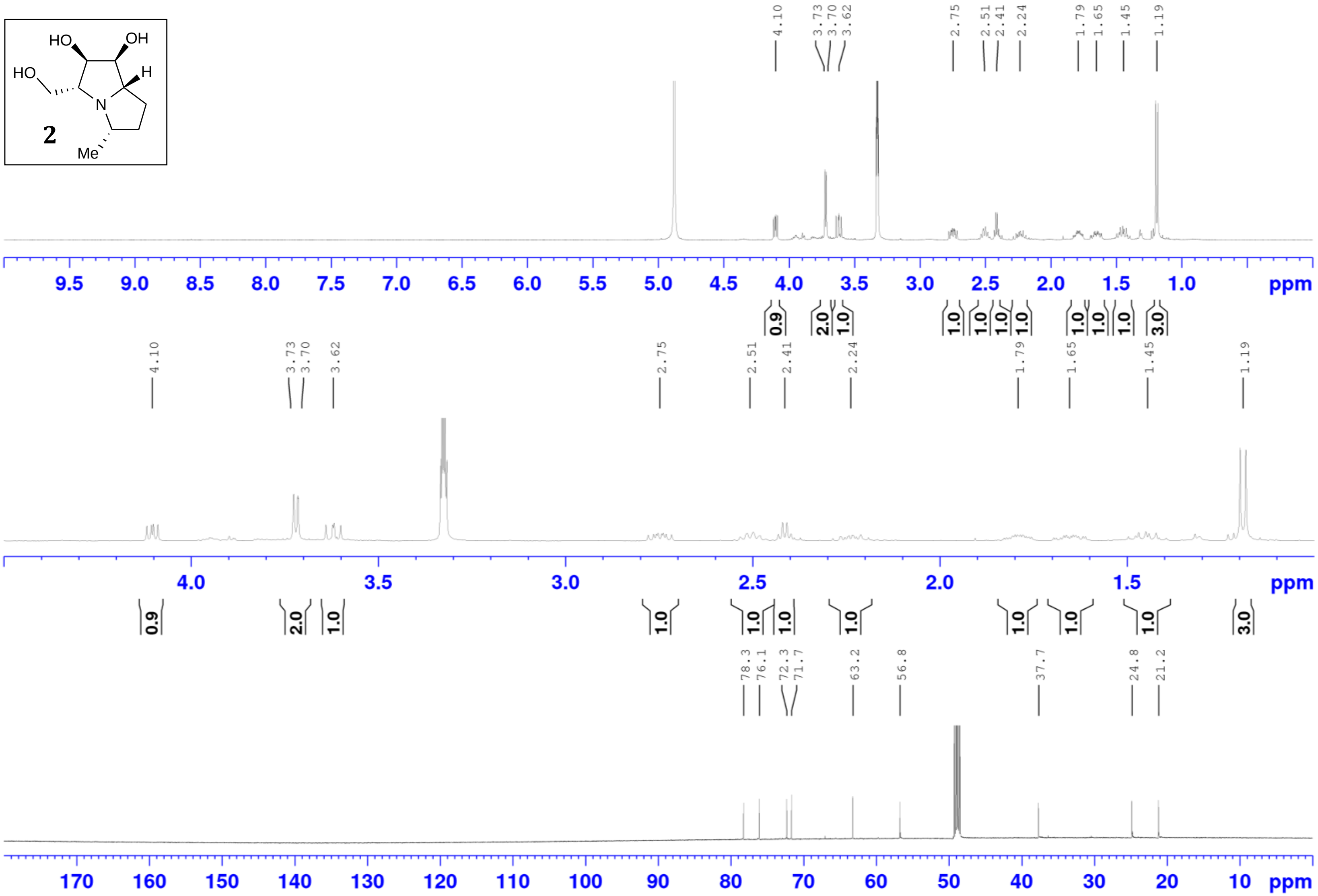

Supplementary Figure 48. ^{1}H and ^{13}C NMR of compound

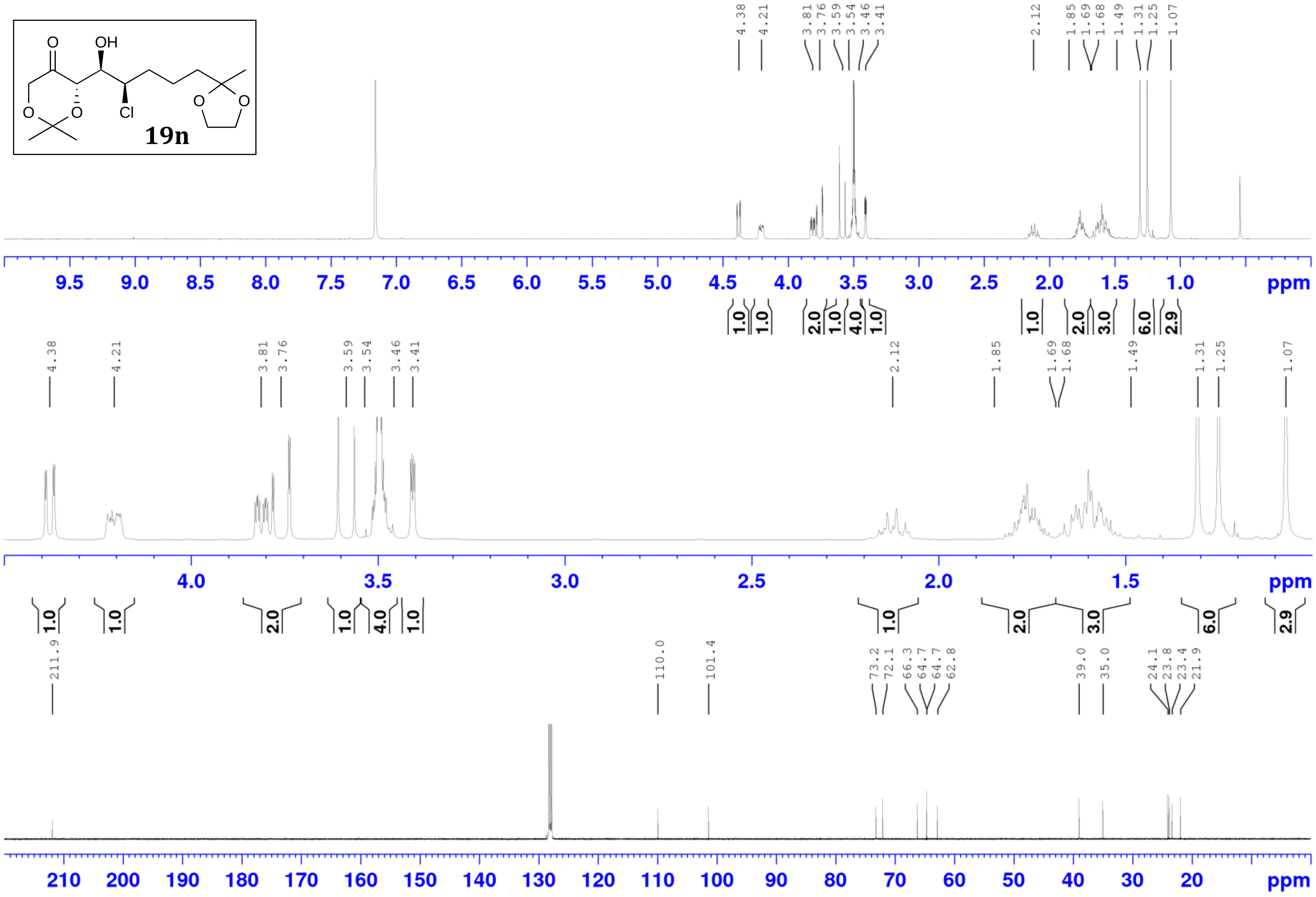

Supplementary Figure 49. ^{1}H and ^{13}C NMR of compound 19n

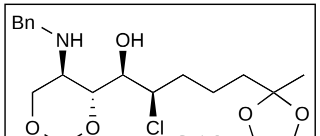

S69

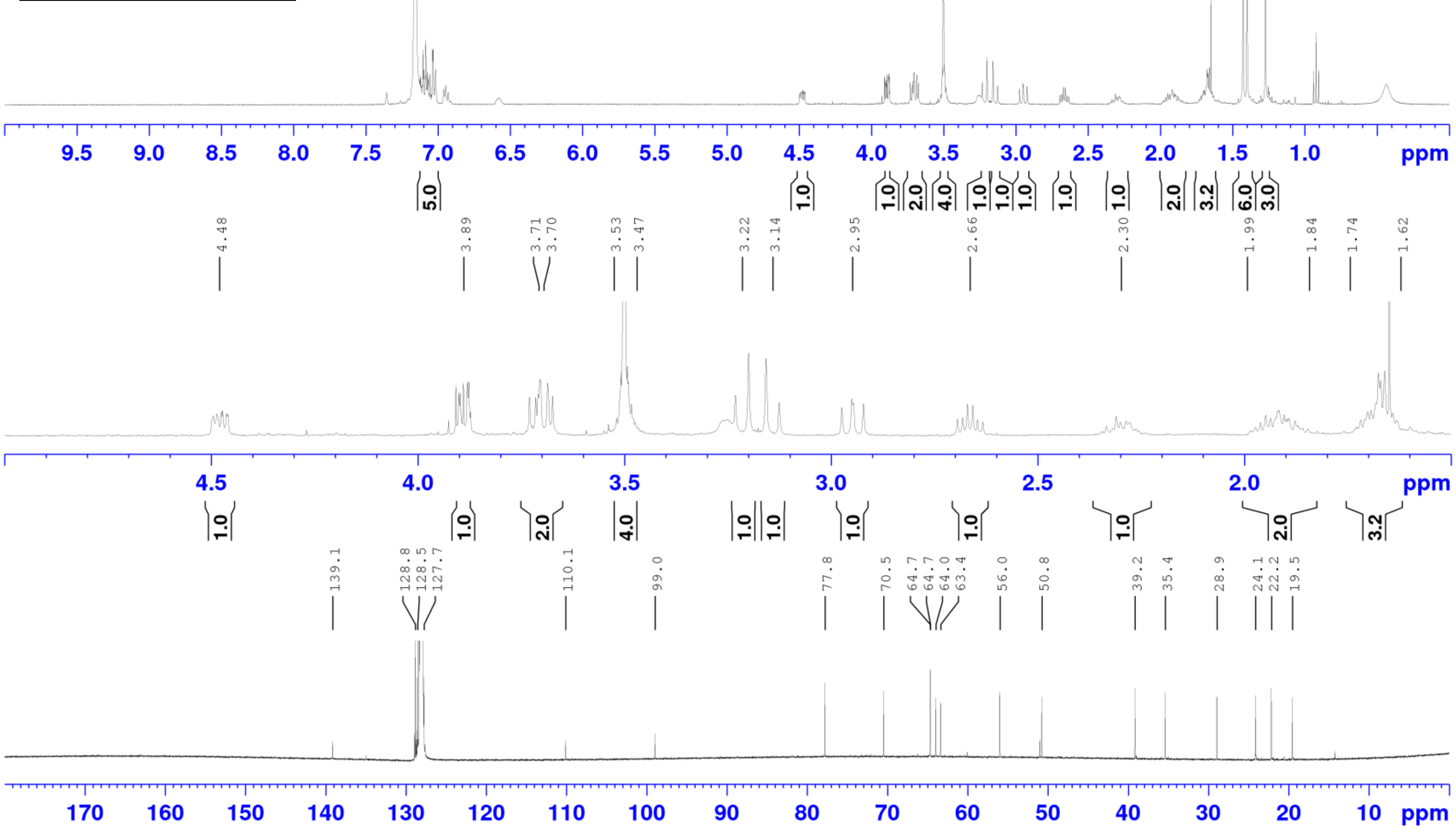

Supplementary Figure 50. ^{1}H and ^{13}C NMR of compound S69

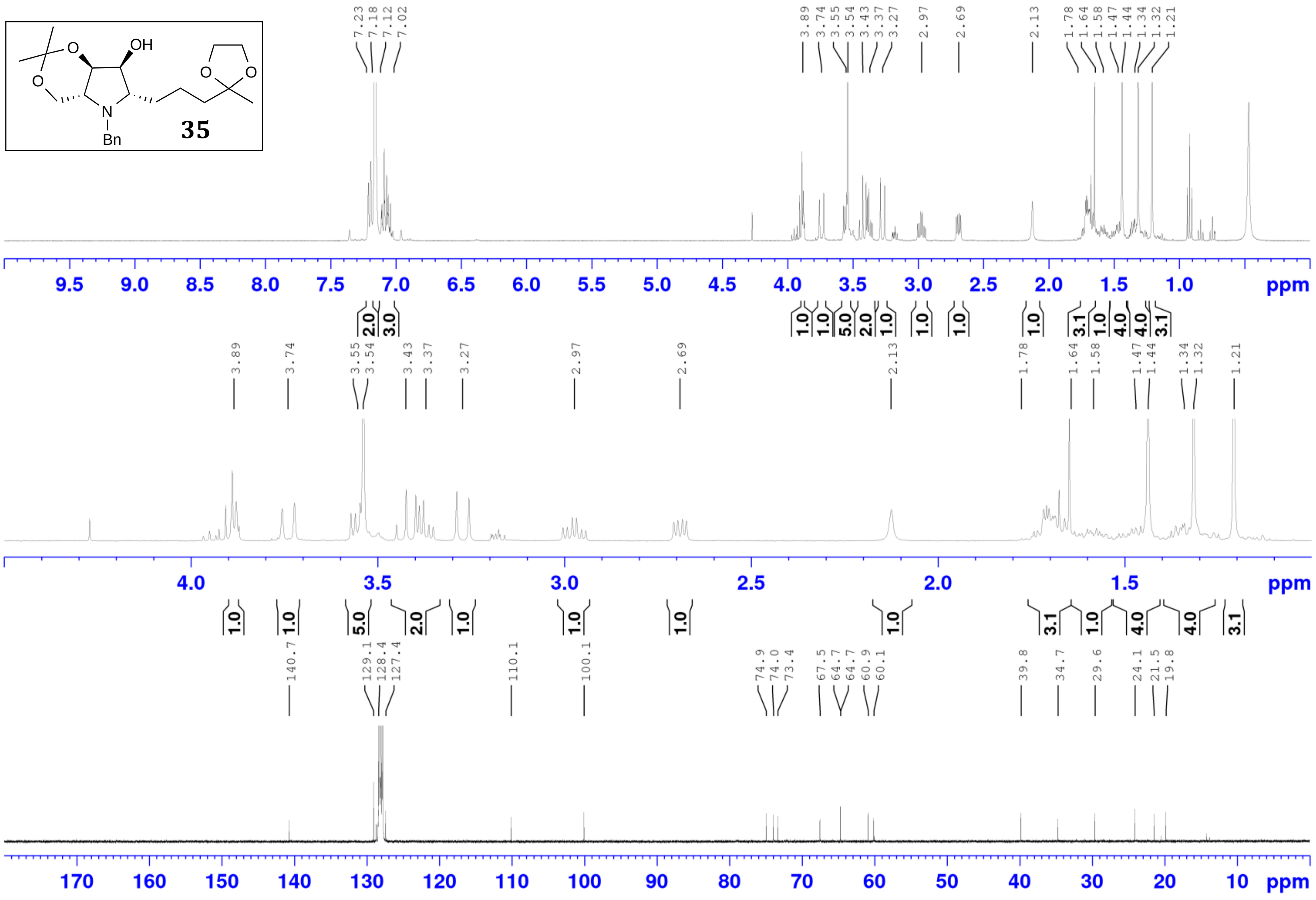

Supplementary Figure 51. ^{1}H and ^{13}C NMR of compound 35

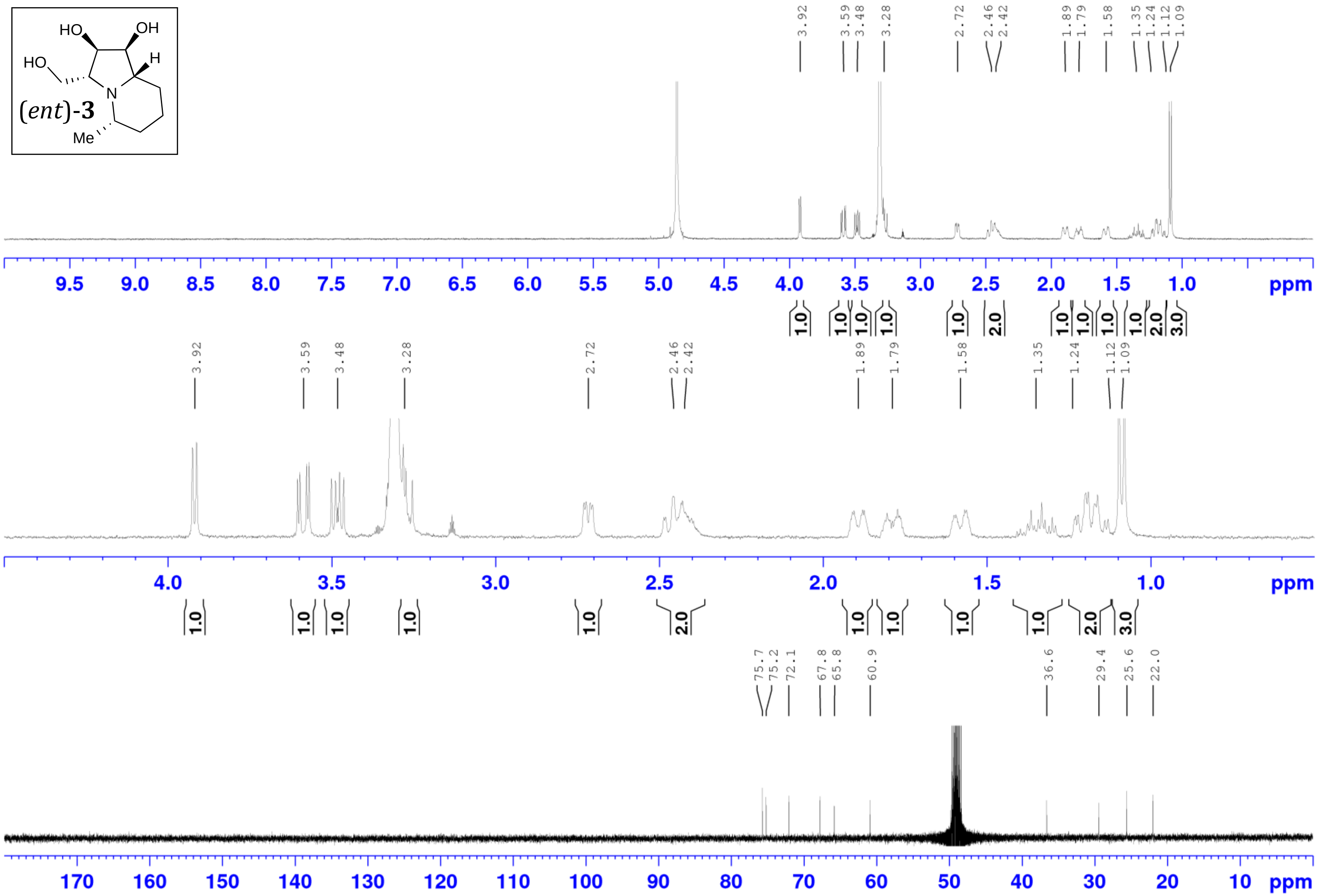

Supplementary Figure 52. ^{1}H **and** ^{13}C **NMR of compound (*ent*)-3**

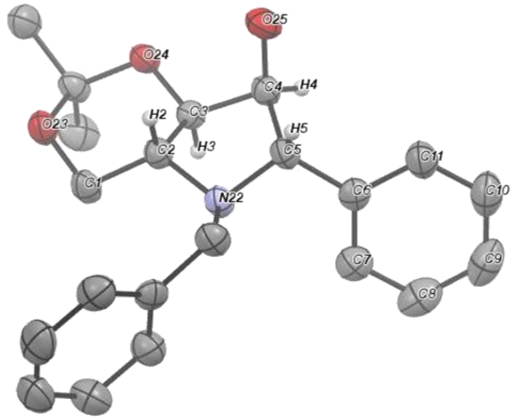

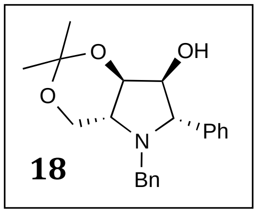

Supplementary Figure 53 – ORTEP representation at 50% ellipsoid probability of **18** in the solid-state structure of **18**. Hydrogen atoms were removed for clarity, except those on the asymmetric carbon atoms.

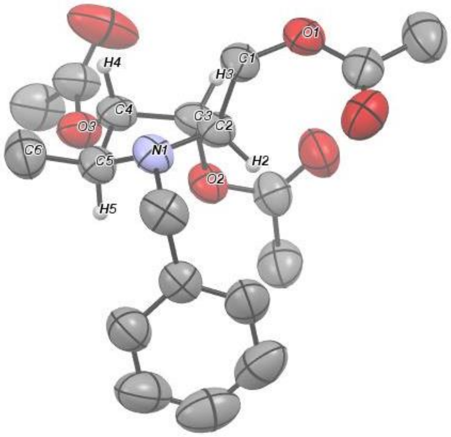

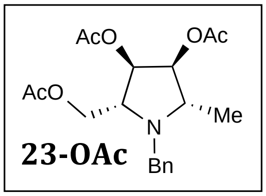

Supplementary Figure 54 – ORTEP representation at 50% ellipsoid probability of **23-OAc** in the solid-state structure of **23-OAc**. Hydrogen atoms were removed for clarity, except those on the asymmetric carbon atoms.

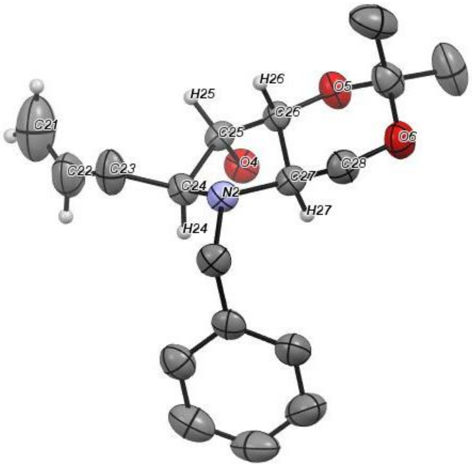

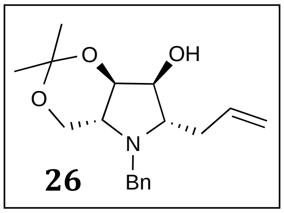

Supplementary Figure 55 – ORTEP representation at 50% ellipsoid probability of **26** in the solid-state structure of **26**. Hydrogen atoms were removed for clarity, except those on the asymmetric carbon atoms.

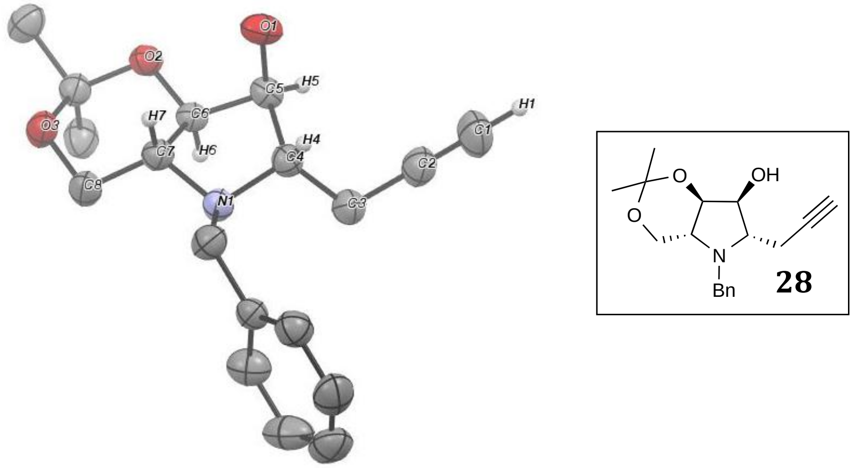

Supplementary Figure 56 – ORTEP representation at 50% ellipsoid probability of **28** in the solid-state structure of **28**. Hydrogen atoms were removed for clarity, except those on the asymmetric carbon atoms.

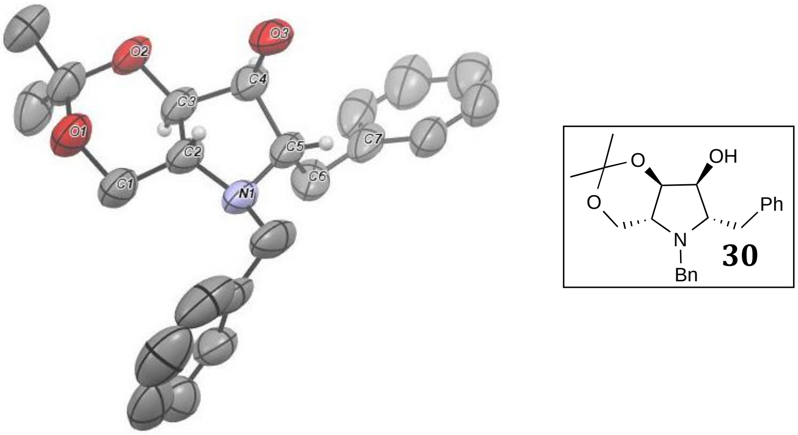

Supplementary Figure 57 – ORTEP representation at 50% ellipsoid probability of **30** in the solid-state structure of **30**. Hydrogen atoms were removed for clarity, except those on the asymmetric carbon atoms.

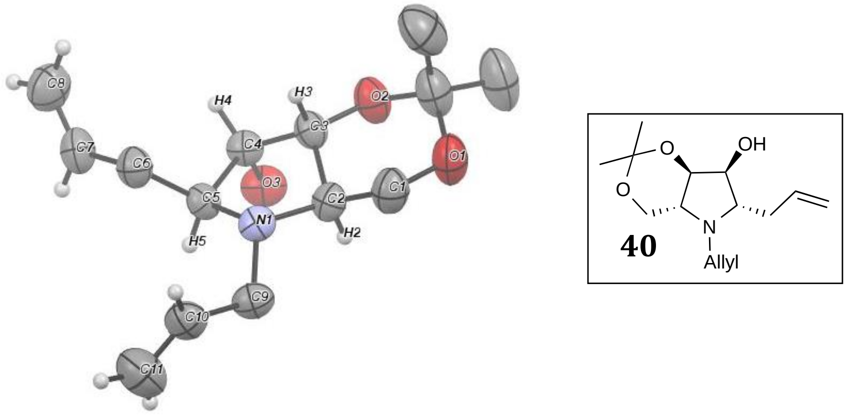

Supplementary Figure 58 – ORTEP representation at 50% ellipsoid probability of **40** in the solid-state structure of **40**. Hydrogen atoms were removed for clarity, except those on the asymmetric carbon atoms and the alkenes.

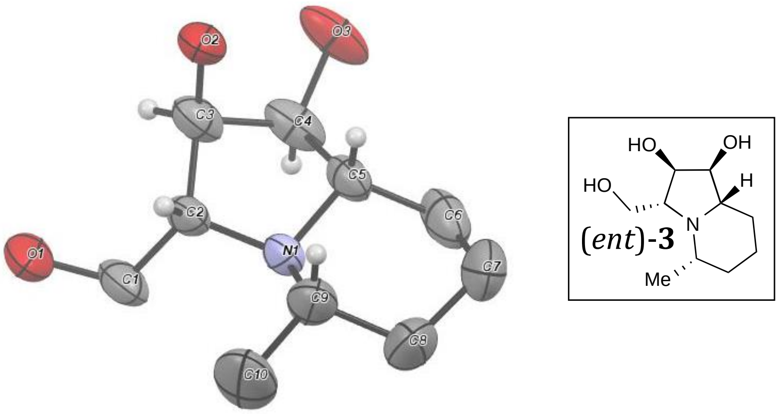

Supplementary Figure 59 – ORTEP representation at 50% ellipsoid probability of (*ent*)-**3** in the solid-state structure of (*ent*)-**3**. Hydrogen atoms were removed for clarity, except those on the asymmetric carbon atoms.

Supplementary Table 1

Copies of HPLC Chromatograms

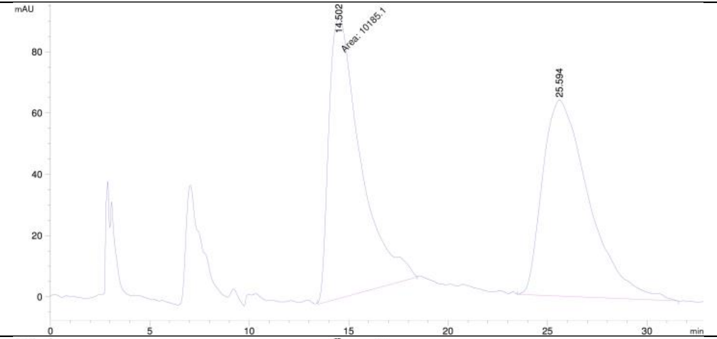

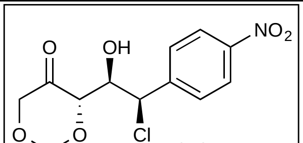

(±)19g

Peak #	RetTime [min]	Type	Width [min]	Area mAU *s	Height [mAU]	Area %
1	14.502	MM T	1.8551	1.01851e4	91.50354	49.5071
2	25.594	VB	2.0447	1.03879e4	63.96331	50.4929

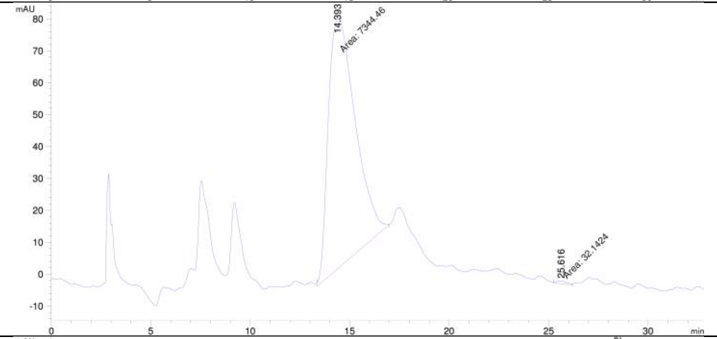

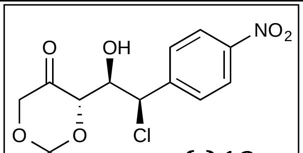

(-)19g

Peak #	RetTime [min]	Type	Width [min]	Area mAU *s	Height [mAU]	Area %
1	14.393	MM T	1.5618	7344.45703	78.37552	99.5643
2	25.616	MM T	0.5905	32.14238	9.07210e-1	0.4357

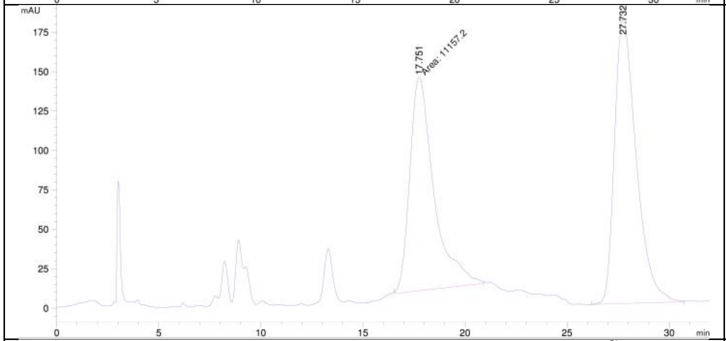

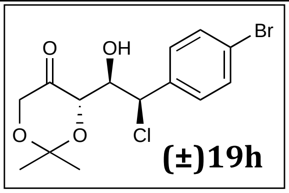

(±)19h

Peak #	RetTime [min]	Type	Width [min]	Area mAU *s	Height [mAU]	Area %
1	17.751	MM T	1.3730	1.11572e4	135.43707	45.4825
2	27.732	BB	1.1126	1.33735e4	180.04979	54.5175

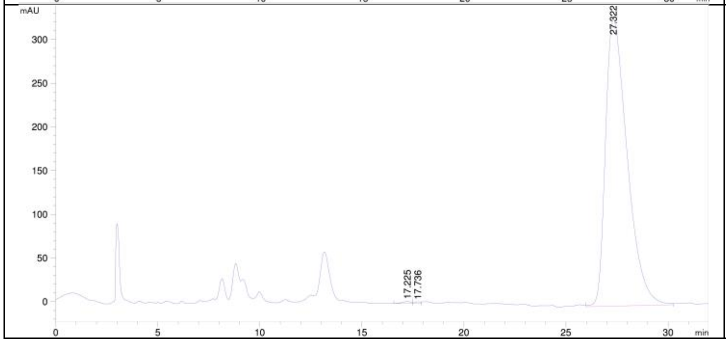

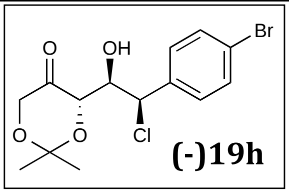

(-)19h

Peak #	RetTime [min]	Type	Width [min]	Area mAU *s	Height [mAU]	Area %
1	17.225	BV	0.4195	73.04308	2.37815	0.2996
2	17.736	VV	0.3127	46.51564	2.08507	0.1908
3	27.322	VB	1.1130	2.42640e4	327.61145	99.5097

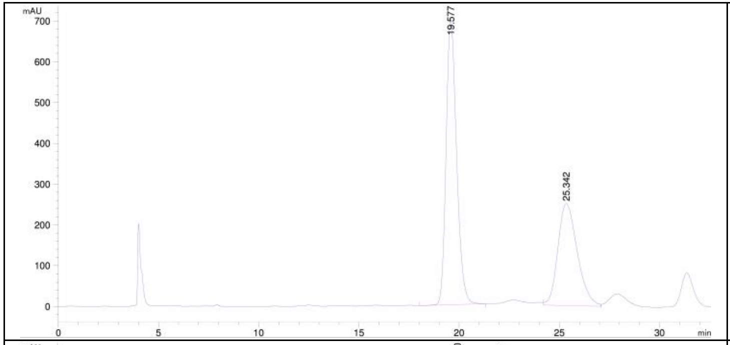

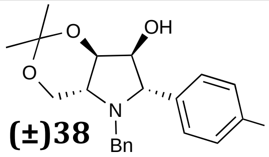

(±)38

Peak #	RetTime [min]	Type	Width [min]	Area mAU *s	Height [mAU]	Area %
1	19.577	VB	0.5604	2.54146e4	697.74414	60.1797
2	25.342	VV	1.0173	1.68166e4	249.66994	39.8203

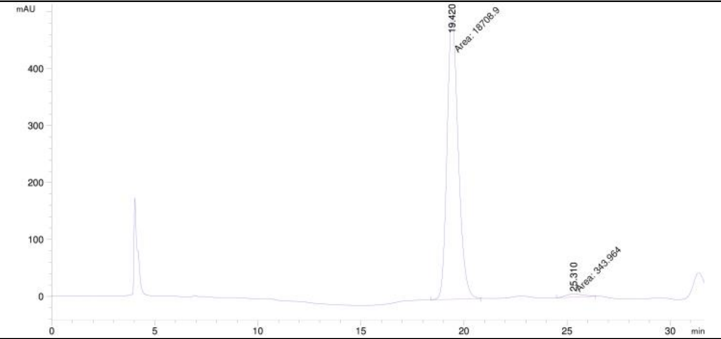

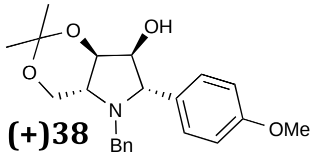

(+)38

Peak #	RetTime [min]	Type	Width [min]	Area mAU *s	Height [mAU]	Area %
1	19.420	MM T	0.6313	1.87089e4	493.94278	98.1947
2	25.310	MM T	0.9940	343.96426	5.76758	1.8053

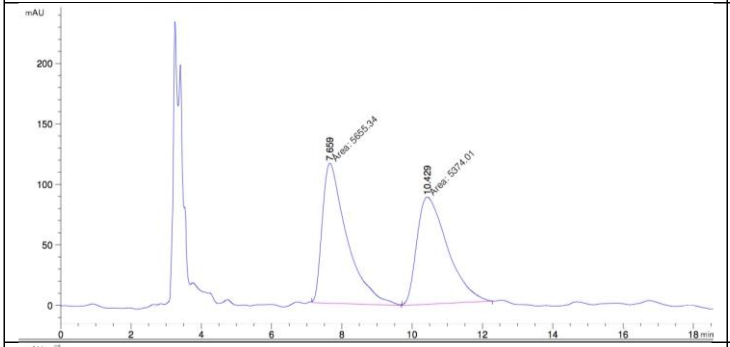

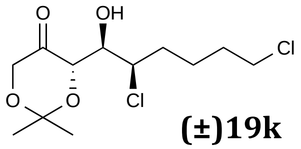

(±)19k

Peak #	RetTime [min]	Type	Width [min]	Area mAU *s	Height [mAU]	Area %
1	7.659	MM T	0.8149	5655.33545	115.65909	51.2754
2	10.429	MM T	1.0099	5374.00928	88.68598	48.7246

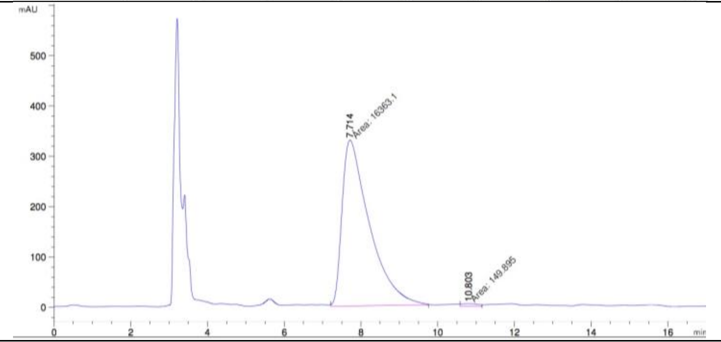

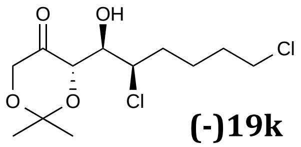

(-)19k

Peak #	RetTime [min]	Type	Width [min]	Area mAU *s	Height [mAU]	Area %
1	7.714	MM T	0.8271	1.63631e4	329.70929	99.0923
2	10.803	MM T	0.5038	149.89484	4.95869	0.9077

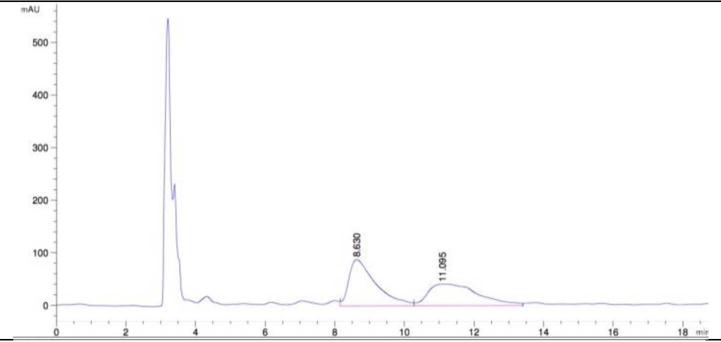

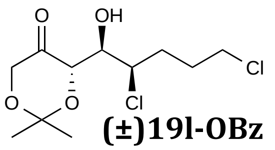

(±)19l-OBz

Peak #	RetTime [min]	Type	Width [min]	Area mAU *s	Height [mAU]	Area %
1	8.630	VV	0.7767	4770.19287	88.38043	53.3060
2	11.095	VV	1.3404	4178.51074	41.20377	46.6940

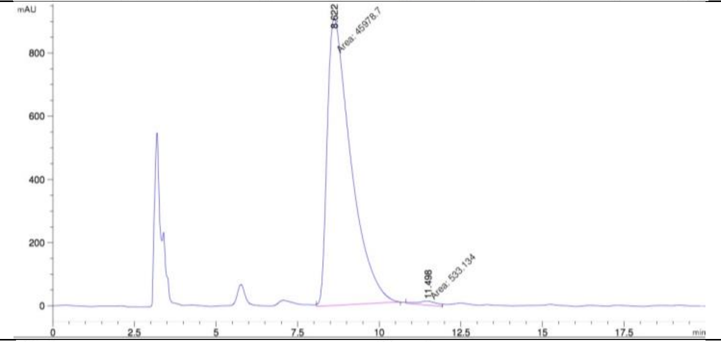

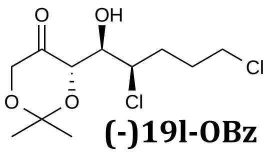

(-)19l-OBz

Peak #	RetTime [min]	Type	Width [min]	Area mAU *s	Height [mAU]	Area %
1	8.622	MM T	0.8439	4.59787e4	908.00916	98.8538
2	11.498	MM T	0.7073	533.13403	12.56267	1.1462

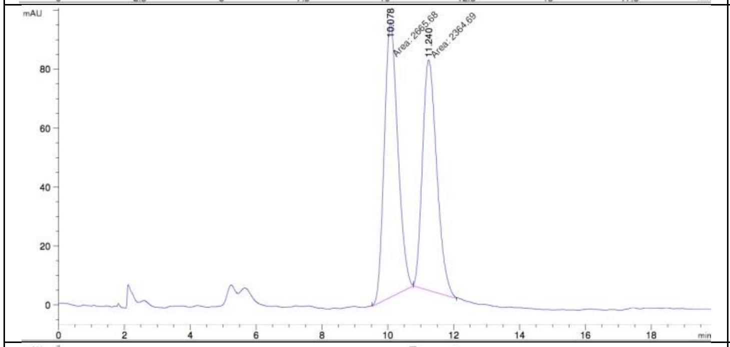

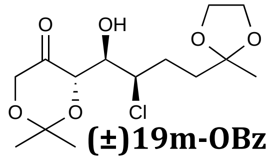

(±)19m-OBz

Peak #	RetTime [min]	Type	Width [min]	Area mAU *s	Height [mAU]	Area %
1	10.078	MM T	0.4762	2665.67773	93.29206	52.9917
2	11.240	MM T	0.5036	2364.69336	78.26585	47.0083

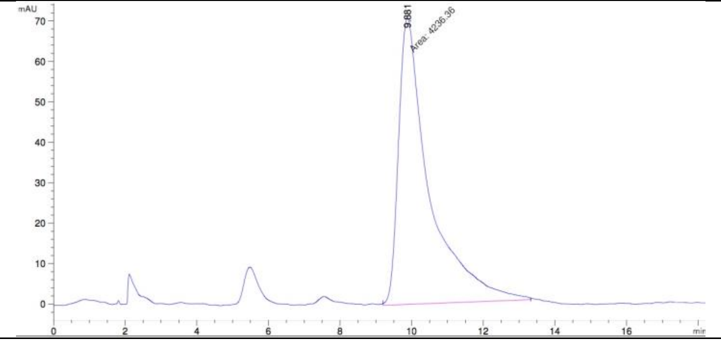

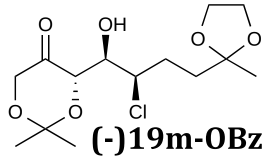

(-)19m-OBz

Peak #	RetTime [min]	Type	Width [min]	Area mAU *s	Height [mAU]	Area %
1	9.881	MM T	0.9972	4236.35986	70.80127	100.0000

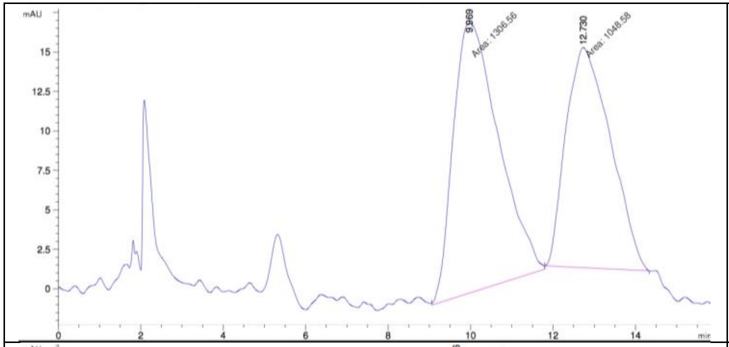

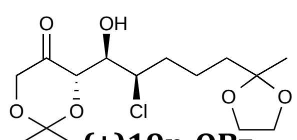

(±)19n-OBz

Peak #	RetTime [min]	Type	Width [min]	Area mAU *s	Height [mAU]	Area %
1	9.969	MM T	1.2828	1306.56250	17.08546	55.4770
2	12.730	MM T	1.2536	1048.58179	13.94075	44.5230

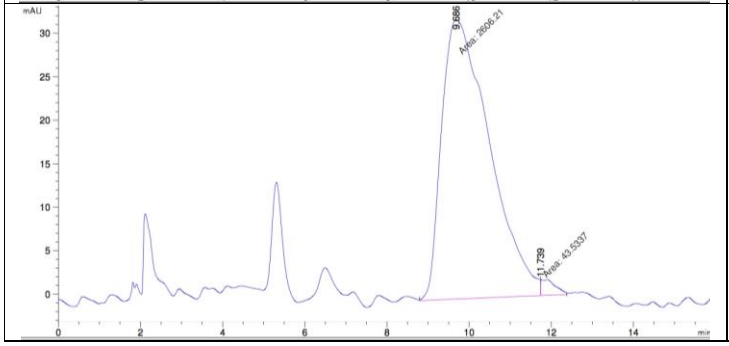

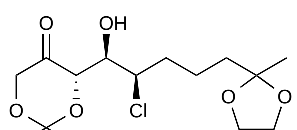

(-)19n-OBz

Peak #	RetTime [min]	Type	Width [min]	Area mAU *s	Height [mAU]	Area %
1	9.686	MF T	1.3540	2606.21411	32.08060	98.3571
2	11.739	FM T	0.3961	43.53367	1.83170	1.6429

Supplementary Table 2

Crystallographic data for pyrrolidine **18** (CCDC #1038918).

	pyrrolidine **18**
CCDC #	1038918
formula	$C_{21}H_{25}NO_3$
formula weight	339.42
crystal system	tetragonal
space group	P_{41}
a (Å)	9.5905(2)
b (Å)	9.5905(2)
c (Å)	19.7424(5)
α (deg)	90
β (deg)	90
γ (deg)	90
V [Å^3]	1815.86(9)
Z, D_{calc} [g/cm^3]	4
T (K)	296(2)
ρ_{calcd} (g/cm^3)	1.242
λ (Å)	0.71073
μ (cm^{-1})	0.082
R indices^a with $I > 2\sigma(I)$ (data)	0.0286
wR_2	0.0726
R_1	0.0304
goodness-of-fit on F^2	1.042
flack parameter	-0.2(2)

Supplementary Table 3

Crystallographic data for pyrrolidine **23-OAc** (CCDC #1038919).

	pyrrolidine **23-OAc**
CCDC #	1038919
formula	$C_{19}H_{25}NO_6$
formula weight	363.40
crystal system	orthorhombic
space group	P_{212121}
a (Å)	8.8437(9)
b (Å)	13.3090(13)
c (Å)	16.8901(17)
α (deg)	90
β (deg)	90
γ (deg)	90
V [Å^3]	1988.0(3)
Z, D_{calc} [g/cm^3]	4
T (K)	296(2)
ρ_{calcd} (g/cm^3)	1.214
λ (Å)	0.71073
μ (cm^{-1})	0.090
R indices^a with $I > 2\sigma(I)$ (data)	0.0389
wR_2	0.1024
R_1	0.0605
goodness-of-fit on F^2	1.018
flack parameter	0.2(4)

Supplementary Table 4

Crystallographic data for pyrrolidine **26** (CCDC #1038920).

	pyrrolidine **26**
CCDC #	1038920
formula	$C_{18}H_{25}NO_3$
formula weight	303.39
crystal system	orthorhombic
space group	P_{212121}
a (Å)	10.6408(3)
b (Å)	17.7283(5)
c (Å)	18.1077(5)
α (deg)	90
β (deg)	90
γ (deg)	90
V [Å^3]	3415.90(17)
Z, D_{calc} [g/cm^3]	8
T (K)	296(2)
ρ_{calcd} (g/cm^3)	1.180
λ (Å)	0.71073
μ (cm^{-1})	0.080
R indices[a] with $I > 2\sigma(I)$ (data)	0.0441
wR_2	0.1061
R_1	0.0705
goodness-of-fit on F^2	1.025
flack parameter	-0.1(4)

Supplementary Table 5

Crystallographic data for pyrrolidine **28** (CCDC #1038921).

	pyrrolidine **28**
CCDC #	1038921
formula	$C_{18}H_{23}NO_3$
formula weight	301.37
crystal system	orthorhombic
space group	P_{212121}
a (Å)	9.3102(3)
b (Å)	9.6848(3)
c (Å)	18.5136(6)
α (deg)	90
β (deg)	90
γ (deg)	90
V [Å^3]	1669.32(9)
Z, D_{calc} [g/cm^3]	4
T (K)	296(2)
ρ_{calcd} (g/cm^3)	1.199
λ (Å)	0.71073
μ (cm^{-1})	0.081
R indices[a] with $I > 2\sigma(I)$ (data)	0.0320
wR_2	0.0798
R_1	0.0351
goodness-of-fit on F^2	1.069
flack parameter	-0.6(4)

Supplementary Table 6

Crystallographic data for pyrrolidine **30** (CCDC #1038922).

	pyrrolidine **30**
CCDC #	1038922
formula	$C_{22}H_{27}NO_3$
formula weight	353.44
crystal system	monoclinic
space group	C_2
a (Å)	30.107(3)
b (Å)	5.8007(5)
c (Å)	11.5447(10)
α (deg)	90
β (deg)	99.634(4)
γ (deg)	90
V [Å^3]	1987.7(3)
Z, D_{calc} [g/cm^3]	4
T (K)	296(2)
ρ_{calcd} (g/cm^3)	1.181
λ (Å)	0.71073
μ (cm^{-1})	0.078
R indices^a with $I > 2\sigma(I)$ (data)	0.0445
wR_2	0.1244
R_1	0.0615
goodness-of-fit on F^2	0.998
flack parameter	-0.8(4)

Supplementary Table 7

Crystallographic data for pyrrolidine **40** (CCDC #1038923).

	pyrrolidine **40**
CCDC #	1038923
formula	$C_{14}H_{23}NO_3$
formula weight	253.33
crystal system	tetragonal
space group	P_{41212}
a (Å)	12.8285(6)
b (Å)	12.8285(6)
c (Å)	18.0935(9)
α (deg)	90
β (deg)	90
γ (deg)	90
V [Å^3]	2977.7(3)
Z, D_{calc} [g/cm^3]	8
T (K)	296(2)
ρ_{calcd} (g/cm^3)	1.242
λ (Å)	0.71073
μ (cm^{-1})	0.079
R indices^a with $I > 2\sigma(I)$ (data)	0.0386
wR_2	0.0883
R_1	0.0633
goodness-of-fit on F^2	1.021
flack parameter	0.5(7)

Supplementary Table 8

Crystallographic data for indolizidine (*ent*)-**3** (CCDC #1038924).

	indolizidine (*ent*)-**3**
CCDC #	1038924
formula	$C_{10}H_{16}NO_3$
formula weight	198.24
crystal system	triclinic
space group	P_1
a (Å)	5.0728(3)
b (Å)	14.5938(7)
c (Å)	15.1058(8)
α (deg)	83.648(3)
β (deg)	81.203(3)
γ (deg)	83.714(3)
V [Å^3]	1093.47(10)
Z, D_{calc} [g/cm^3]	4
T (K)	296(2)
ρ_{calcd} (g/cm^3)	1.204
λ (Å)	0.71073
μ (cm^{-1})	0.081
R indices[a] with $I > 2\sigma(I)$ (data)	0.0664
wR_2	0.2158
R_1	0.0708
goodness-of-fit on F^2	1.112
flack parameter	0.11(10)

Supplementary Methods
General
(*S*)- and (*R*)-Proline (99% purity) were purchased from Sigma-Aldrich and Alfa Aesar, respectively. All reactions described were performed in flame-dried flasks at ambient temperature and under inert atmosphere (N_2) unless otherwise specified. Column chromatography was carried out with 230-400 mesh silica gel (E. Merck, Silica Gel 60). Concentration and removal of trace solvents was done via a Buchi rotary evaporator using acetone-dry-ice condenser and a Welch vacuum pump.

Nuclear magnetic resonance (NMR) spectra were recorded using deuterochloroform ($CDCl_3$), deuteromethanol (CD_3OD), deuterobenzene (C_6D_6) or deuterium oxide (D_2O) as the solvent. Signal positions (δ) are given in parts per million from tetramethylsilane (δ 0) and were measured relative to the signal of the solvent (**^{1}H NMR:** $CDCl_3$: δ 7.26; CD_3OD: δ 3.31; C_6D_6: δ 7.16, D_2O: δ 4.79; **^{13}C NMR:** $CDCl_3$: δ 77.0; CD_3OD: δ 49.0; C_6D_6: δ 128.06). Coupling constants (*J* values) are given in Hertz (Hz) and are reported to the nearest 0.1 Hz. ^{1}H NMR spectral data are tabulated in the order: multiplicity (*s*, singlet; *d*, doublet; *t*, triplet; *q*, quartet; s_{ept}, septet; *m*, multiplet; $_{br}$ broad; $_{app}$ apparent), coupling constants, number of protons. NMR spectra were recorded on a Bruker Avance 600 equipped with a QNP or TCI cryoprobe (600 MHz), Bruker 400 (400 MHz) or Bruker 500 (500 MHz). Diastereomeric ratios (dr) are based on analysis of crude ^{1}H-NMR spectra. Proton assignments in ^{1}H spectra are based on analysis of ^{1}H-^{1}H-COSY and HSQC spectra. Carbon assignments in ^{13}C spectra are based on analysis of HSQC and HMBC spectra. High performance liquid chromatography (**HPLC**) analysis was performed on an Agilent 1100 HPLC, equipped with a variable wavelength UV-Vis detector and Diacel Chiralpak AD or Chiralcel OD-H chiral column (0.46 cm x 25 cm). Enantiomeric excess (*ee*) was determined on the corresponding benzoylated esters (see General Procedure F) of all *syn*-chlorohydrin aldol products except for aldol adducts that contain an aryl function, which did not require derivatization. Infrared (**IR**) spectra were recorded neat on a Perkin Elmer Spectrum Two FTIR spectrometer. Only selected, characteristic absorption data are provided for each compound. Optical rotation was measured on a Perkin-Elmer Polarimeter 341 at 589 nm. Microwaves reactions were performed in a CEM Discover LabMate microwave reactor. Hydrogenolysis/hydrogenation reactions were performed using a ThalesNano H-Cube continuous-flow reactor. DOWEX 1X8-100 (HO^- form) was prepared by washing a column of DOWEX 1X8-100-Cl resin with a 1M aqueous solution of NaOH until no more AgCl precipitated. The resin was then rinsed with H_2O and MeOH thoroughly before usage. Single crystal X-ray crystallographic analyses of were performed on a Bruker X8 APEX II diffractometer.

Single Crystal X-Ray Crystallographic Analysis

For cifs of these compounds, see Supplementary Data 1-7.

COMPOUND 18
Single crystal X-ray crystallographic analysis of compound **18** was performed on a Bruker X8 APEX II diffractometer with graphite monochromated Mo-Kα radiation. A colourless block crystal was mounted on a glass fiber. The data were collected at 296(2) K. Data was collected in a series of ω and φ in 0.50° widths with 10.0 s exposures. The crystal-to-detector distance was 50 mm. The structure was solved by direct methods (intrinsic methods),[1] and refined by least-squares procedures using ShelXle.[2] All non-hydrogen atoms were refined anisotropically. A summary of the crystal data and experimental parameters for structure determinations is given in Supplementary Table 2.

COMPOUND 23-OAc

Single crystal X-ray crystallographic analysis of compound **23-OAc** was performed on a Bruker X8 APEX II diffractometer with graphite monochromated Mo-Kα radiation. A colourless block crystal was mounted on a glass fiber. The data were collected at 296(2) K. Data was collected in a series of ω in 0.50° widths with 5.0 s exposures. The crystal-to-detector distance was 50 mm. The structure was solved by direct methods (intrinsic methods),[1] and refined by least-squares procedures using ShelXle.[2] All non-hydrogen atoms were refined anisotropically. All crystal structure plots were produced using Mercury 3.3. A summary of the crystal data and experimental parameters for structure determinations is given in Supplementary Table 3.

COMPOUND 26

Single crystal X-ray crystallographic analysis of compound **26** was performed on a Bruker X8 APEX II diffractometer with graphite monochromated Mo-Kα radiation. A colourless block crystal was mounted on a glass fiber. The data were collected at 296(2) K. Data was collected in a series of ω in 0.50° widths with 5.0 s exposures. The crystal-to-detector distance was 50 mm. The structure was solved by direct methods (intrinsic methods),[1] and refined by least-squares procedures using ShelXle.[2] All non-hydrogen atoms were refined anisotropically. A summary of the crystal data and experimental parameters for structure determinations is given in Supplementary Table 4.

COMPOUND 28

Single crystal X-ray crystallographic analysis of compound **28** was performed on a Bruker X8 APEX II diffractometer with graphite monochromated Mo-Kα radiation. A colourless block crystal was mounted on a glass fiber. The data were collected at 296(2) K. Data was collected in a series of ω in 0.50° widths with 10.0 s exposures. The crystal-to-detector distance was 50 mm. The structure was solved by direct methods (intrinsic methods),[1] and refined by least-squares procedures using ShelXle.[2] All non-hydrogen atoms were refined anisotropically. A summary of the crystal data and experimental parameters for structure determinations is given in Supplementary Table 5.

COMPOUND 30

Single crystal X-ray crystallographic analysis of compound **30** was performed on a Bruker X8 APEX II diffractometer with graphite monochromated Mo-Kα radiation. A colourless block crystal was mounted on a glass fiber. The data were collected at 296(2) K. Data was collected in a series of ω and φ in 0.50° widths with 5.0 s exposures. The crystal-to-detector distance was 50 mm. The structure was solved by direct methods (intrinsic methods),[1] and refined by least-squares procedures using ShelXle.[2] All non-hydrogen atoms were refined anisotropically. A summary of the crystal data and experimental parameters for structure determinations is given in Supplementary Table 6.

COMPOUND 40

Single crystal X-ray crystallographic analysis of compound **40** was performed on a Bruker X8 APEX II diffractometer with graphite monochromated Mo-Kα radiation. A colourless block crystal was mounted on a glass fiber. The data were collected at 296(2) K. Data was collected in a series of ω in 0.50° widths with 7.0 s exposures. The crystal-to-detector distance was 50 mm. The structure was solved by direct methods (intrinsic methods),[1] and refined by least-squares procedures using ShelXle.[2] All non-hydrogen atoms were refined anisotropically. A summary of the crystal data and experimental parameters for structure determinations is given in Supplementary Table 7.

COMPOUND (*ent*)-3

Single crystal X-ray crystallographic analysis of compound (*ent*)-**3** was performed on a Bruker X8 APEX II diffractometer with graphite monochromated Cu-Kα radiation. A colourless block crystal was mounted on a glass fiber. The data were collected at 296(2) K. Data was collected in a series of ω and φ in 0.80° widths with 2.0 s exposures for $2\theta < 30°$; 5.0 s for $30° < 2\theta < 70°$ and 10.0 s for $2\theta > 70$ °. The crystal-to-detector distance was 50 mm. The structure was solved by direct methods (intrinsic methods),[1] and refined by least-squares procedures using ShelXle.[2] All non-hydrogen atoms were refined anisotropically. A summary of the crystal data and experimental parameters for structure determinations is given in Supplementary Table 8.

General Procedures

General Procedure A (one-pot organocatalytic α-chlorination/aldol reaction)

The aldehyde (1.00 equiv.) was added to a stirred suspension of *N*-chlorosuccinimide (NCS) (1.05 equiv.) and (*S*)-Proline (0.80 equiv.) in CH_2Cl_2 (0.2 M) at room temperature (unless otherwise specified). 2,2-Dimethyl-1,3-dioxan-5-one (**8**) (1.05 equiv.) was then added and the resulting mixture was stirred for a total of 24 hours, or until complete consumption of the intermediate α-chloroaldehyde was observed by ^{1}H-NMR spectroscopic analysis of small samples removed from the reaction mixture. The reaction mixture was then diluted with CH_2Cl_2 and the organic layer was removed and washed twice with water and once with brine. The organic layer was then dried ($MgSO_4$), concentrated under reduced pressure and the crude product was purified by flash chromatography as indicated.

General Procedure B (1,3-*syn*-selective reductive amination)

To a stirred solution of the chlorohydrin (1.0 equiv.) in THF (0.1 M) was added the corresponding amine (2.0-2.5 equiv.) and glacial acetic acid (1.0 equiv.), and the resulting mixture was stirred at 20°C for 2 hours or until complete conversion into the corresponding imine was accomplished (as determined by ^{1}H-NMR spectroscopic analysis of small samples removed from the reaction mixture). $NaCNBH_3$ (2.5 equiv.) was then added and the mixture was stirred for a further 1 hour. The reaction mixture was then diluted with CH_2Cl_2 to a concentration of 0.05 M and treated with water. The layers were separated and the organic layer was washed with brine, dried ($MgSO_4$), and concentrated under reduced pressure. The crude product was purified by flash chromatography as indicated.

General Procedure C (thermal cyclization with $NaHCO_3$ in toluene)

A stirred solution of the chloroaminoalcohol (1.0 equiv) and $NaHCO_3$ (50 wgt%) in dry PhMe (0.1 M) was heated to 105 °C and maintained at this temperature in a sealed vessel until no starting material remained (approximately 24-48 hours). The reaction mixture was then diluted with CH_2Cl_2 and washed with water and brine. The organic layer was dried ($MgSO_4$), concentrated under reduced pressure, and the crude product was purified by flash chromatography as indicated.

General Procedure D (thermal cyclization in MeOH)

The chloroaminoalcohol (1.0 equiv.) was added to a microwave vial and dissolved in MeOH. The vial was then sealed in a CEM Discover LabMate microwave reactor and the resulting mixture was heated at 120 °C (as monitored by a vertically focused IR temperature sensor) for 30 minutes, or until no starting material remained (as determined by TLC analysis). The reaction mixture was then concentrated under reduced pressure. Oftentimes, to facilitate characterization, the crude product was peracetylated and purified by flash chromatography as indicated.

General Procedure E (ring closing metathesis)
A stirred solution of pyrrolidine (1.0 equiv.) and Hoveyda-Grubbs catalyst 2nd Gen. (5 mol%) in degassed PhMe (0.01 M) was heated to 60°C and maintained at this temperature for 2 hours. The reaction mixture was then concentrated and the crude product was purified by flash chromatography as indicated.

General Procedure F (benzoylation)
To a stirred solution of racemic or enantiomerically enriched *syn*-chlorohydrin (1.0 equiv) in dry pyridine (0.1 M) was added benzoyl chloride (2.0 equiv) and dimethylaminopyridine (cat.), and the resulting mixture was stirred at room temperature for 24 hours. The reaction mixture was then diluted with EtOAc and washed sequentially with aqueous HCl (3 x 1 mL, 1 M), saturated $NaHCO_3$ (1 mL), and brine (1 mL). The organic layer was dried ($MgSO_4$), concentrated under reduced pressure, and the crude product was purified by flash chromatography (pentane-EtOAc, 9:1).

General Procedure G (hydrogenolysis/hydrogenation)
A solution of the *N*-benzyl pyrrolidine in a given solvent was passed twice through an H-Cube continuous-flow reactor using a 30 mm 10% Pd/C cartridge. Conditions (unless otherwise indicated): Temperature = 60°C; Flow rate = 0.8 mL/min; H_2 Pressure = 90 bar. The resulting solution was concentrated under reduced pressure, and the crude product was used without further purification.

General Procedure H (removal of the acetal protecting group)
A solution of the protected iminocyclitol and PPTS (cat.) in H_2O/MeOH (1:1) was added to a microwave vial. The vial was sealed in a CEM Discover LabMate microwave reactor and the resulting mixture was heated at 100 °C (as monitored by a vertically focused IR temperature sensor) for 30 minutes. The resulting solution was concentrated under reduced pressure, and the crude product was used without further purification unless otherwise indicated.

General Procedure I (acetylation)
A solution of the iminocyclitol in pyridine/Ac_2O (2:1) was stirred for 16 hours at 20°C. The reaction mixture was diluted with EtOAc and washed with a saturated aqueous solution of $NaHCO_3$ and brine. The organic layer was dried ($MgSO_4$) and concentrated under reduced pressure. The crude product was purified by flash chromatography (pentane-EtOAc).

Synthetic Methods and Characterization Data

Preparation of Aldol Adduct (11)
Following General Procedure **A**, a solution of pentanal (100 μL, 0.94 mmol), NCS (132 mg, 0.99 mmol), (*S*)-Proline (86 mg, 0.77 mmol) and dioxanone **8** (118 μL, 0.99 mmol) in CH_2Cl_2 (5 mL) was stirred for 24 hours. Purification of the crude *syn*- and *anti*-chlorohydrins (dr = 6:1) by flash chromatography (pentane-EtOAc 9:1) afforded chlorohydrin **11** (146 mg, 62% yield) as a white solid.

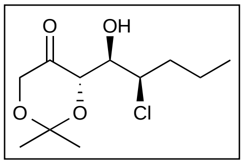

Data for **11**:[3] See reference 3.

Preparation of Chloroaminoalcohol (12a)

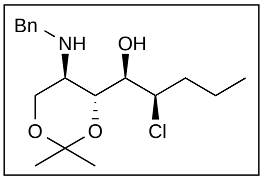

Following General Procedure **B**, a solution of **11**[3] (112 mg, 0.447 mmol), $BnNH_2$ (150 μL, 1.37 mmol) and glacial AcOH (31 μL, 0.448 mmol) in THF (4.5 mL) was stirred for 2 hours then reacted with $NaCNBH_3$ (85 mg, 1.4 mmol) for 1 hour. Purification of the crude product by flash chromatography (pentane-EtOAc 65:35) afforded *syn*-chloroaminoalcohol **12a** (152 mg, 99% yield) as a colourless oil.

Data for **12a**: R_f (pentane-EtOAc 1:1) 0.56.
IR (neat): υ = 3286, 2994, 2958, 1454, 1374, 1263, 1198, 1092, 860, 750, 699 cm^{-1}.
^{1}H-NMR (600 MHz, $CDCl_3$): δ = 7.34-7.26 (*m*, 5H), 4.21 (*ddd*, *J* = 4.6 Hz, *J* = 9.6 Hz, 1H), 4.08 (*dd*, *J* = 5.1 Hz, *J* = 11.5 Hz, 1H), 3.93 (*d*, *J* = 12.6 Hz, 1H), 3.79 (*d*, *J* = 12.6 Hz, 1H), 3.77-3.75 (*m*, 2H), 3.62 (*dd*, *J* = 9.6 Hz, *J* = 11.3 Hz, 1H), 2.97 (*ddd*, *J* = 5.1 Hz, *J* = 9.4 Hz, 1H), 1.93 (*m*, 1H), 1.73 (*m*, 1H), 1.60 (*m*, 1H), 1.47 (*s*, 3H), 1.44 ppm (*m*, 1H), 1.36 (*s*, 3H), 0.95 ppm (*t*, *J* = 7.4 Hz, 3H).
^{13}C-NMR (151 MHz, $CDCl_3$): δ = 137.8, 128.8, 128.4, 127.9, 99.1, 77.4, 69.7, 63.2, 62.8, 55.9, 50.8, 36.7, 28.3, 20.1, 19.4, 13.6 ppm.

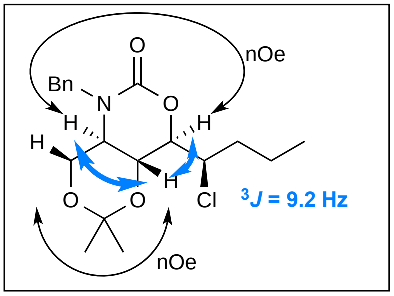

Determination of relative chemistry for the 1,3 syn reductive amination
To a solution of **12a** (20 mg, 0.059 mmol) in CH_2Cl_2 (1.0 mL) was added 1,1'-carbonyldiimidazole (28 mg, 0.18 mmol) and the mixture was stirred at room temperature for 16 hours. The crude mixture was concentrated under reduced pressure and purification of the crude product by flash chromatography (pentane-EtOAc 75:25) afforded the carbamate derivative of **12a** (20 mg, 91 % yield).

Preparation of Iminocyclitol (15)

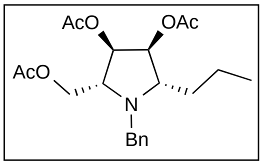

Following General Procedure **D**, a solution of **12a** (45 mg, 0.13 mmol) in MeOH (3.00 mL) was heated for 20 minutes at 120°C. The iminocyclitol HCl salt (34 mg, 85% yield) was obtained as a colourless oil that required no additional purification. To facilitate characterization, the iminocyclitol **15** was peracetylated following General Procedure I. Purification of the crude product by flash chromatography (pentane-EtOAc 7:3) afforded pyrrolidine **15-OAc** as a colourless oil.

Data for **15-OAc**: R_f (pentane-EtOAc 7:3) 0.3; $[\alpha]_D^{20}$ = +8.45 (*c* 1.29 in $CHCl_3$).
IR (neat): υ = 2956, 1743, 1454, 1369, 1227, 1047, 902, 757, 701 cm^{-1}.
^{1}H-NMR (600 MHz, $CDCl_3$): δ = 7.33-7.24 (*m*, 5H), 5.12 (*dd*, *J* = 4.9 Hz, 1H), 5.02 (*dd*, *J* = 4.9 Hz, 1H), 3.97 (*dd*, *J* = 5.7 Hz, *J* = 11.5 Hz, 1H), 3.88 (*d*, *J* = 14.1 Hz, 1H), 3.86 (*dd*, *J* = 4.1 Hz, *J* = 11.6 Hz, 1H), 3.81 (*d*, *J* = 14.1 Hz, 1H), 3.17 (*m*, 1H), 3.01 (*m*, 1H), 2.02 (*s*, 3H), 2.01 (*s*, 3H), 2.01 (*s*, 3H), 1.43-1.23 (*m*, 4H), 0.83 ppm (*t*, *J* = 6.9 Hz, 3H).
^{13}C-NMR (151 MHz, $CDCl_3$): δ = 170.7, 170.0, 169.9, 138.4, 129.2, 128.2, 127.3, 74.4, 72.9, 65.3, 64.5, 63.8, 58.2, 35.1, 20.8, 20.8, 20.7, 18.0, 14.2 ppm.
HRMS (ESI) *m/z* calcd for $C_{21}H_{30}NO_6$ $[M + H]^+$ 392.2068, found 392.2078.

Preparation of Pyrrolidine (16)

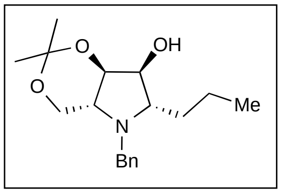

Following General Procedure **C**, a solution of **12a** (38 mg, 0.11 mmol), and $NaHCO_3$ (19 mg) in PhMe (1.0 mL) was stirred for 48 hours at 105°C. The pyrrolidine **16** (34 mg, 99% yield) was isolated as a colourless oil that required no further purification.

Data for **16**: R_f (pentane-EtOAc 8:2) 0.35; $[\alpha]_D^{20}$ = +15 (*c* 0.90 in $CHCl_3$).
IR (neat): υ = 3457, 2989, 2918, 2873, 1449, 1379, 1263, 1208, 1102, 1026, 740, 694 cm^{-1}.
^{1}H-NMR (600 MHz, $CDCl_3$): δ = 7.31-7.21 (*m*, 5H), 3.95 (*d*, *J* = 4.5 Hz, 1H), 3.93 (*d*, *J* = 13.1 Hz, 1H), 3.57 (*dd*, *J* = 4.6 Hz, *J* = 9.8 Hz, 1H), 3.49 (*d*, *J* = 13.1 Hz, 1H), 3.37 (*dd*, *J* = 10.3 Hz, 1H), 3.22 (*dd*, *J* = 4.2 Hz, *J* = 10.4 Hz, 1H), 2.75 (*ddd*, *J* = 4.3 Hz, *J* = 10.1 Hz, 1H), 2.61 (*dd*, *J* = 2.9 Hz, *J* = 6.7 Hz, 1H), 2.17 (*s*, 1H), 1.52-1.45 (*m*, 2H),1.42-1.34 (*m*, 2H), 1.40 (*s*, 3H), 1.39 (*s*, 3H), 0.94 ppm (*t*, *J* = 6.8 Hz, 3H).
^{13}C-NMR (151 MHz, $CDCl_3$): δ = 139.8, 128.8, 128.2, 127.3, 100.1, 74.3, 73.7, 72.7, 67.3, 60.2, 59.8, 36.3, 29.2, 19.9, 19.7, 14.3 ppm.
HRMS (ESI) *m/z* calcd for $C_{18}H_{28}NO_3$ $[M + H]^+$ 306.2064, found 306.2076.

Preparation of Aldol Adduct (17)

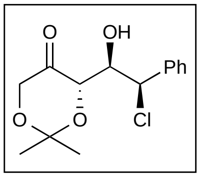

Following General Procedure **A**, a solution of phenylacetaldehyde (664 µL, 5.94 mmol), NCS (832 mg, 6.23 mmol), (*S*)-proline (546 mg, 4.75 mmol) and dioxanone **8** (743 µL, 6.23 mmol) in CH_2Cl_2 (30 mL) was stirred for 24 hours at 0°C. Purification of the crude *syn*- and *anti*-chlorohydrins (dr = 2:1) by flash chromatography (pentane-EtOAc 9:1 to 85:15) afforded *syn*-chlorohydrin **17** (711 mg, 42% yield) as a light yellow oil.

Data for **17**:[3] See reference 3.

Preparation of Pyrrolidine (18)

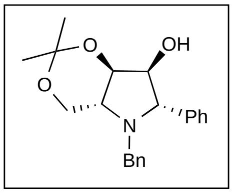

Following General Procedure **B**, a solution of **17**[3] (130 mg, 0.457 mmol), $BnNH_2$ (125 µL, 1.15 mmol) and glacial AcOH (27.0 µL, 0.457 mmol) in THF (4.55 mL) was stirred for 1 hour then reacted with $NaCNBH_3$ (72 mg, 1.15 mmol) for 1 additional hour. Purification of the crude product by flash chromatography (pentane-EtOAc 8:2) afforded pyrrolidine **18** (126 mg, 81% yield) as a crystalline solid.

Data for **18**: mp = 108-111°C (EtOH); R_f (pentane-EtOAc 6:4) 0.81; $[\alpha]_D^{20}$ = +11 (*c* 0.70 in $CHCl_3$).
IR (neat): υ = 3444, 2988, 2874, 1454, 1381, 1210, 1048, 853, 753, 700 cm^{-1}.
^{1}H-NMR (600 MHz, $CDCl_3$): δ = 7.53 (*d*, *J* = 7.4 Hz, 2H), 7.38 (*t*, *J* = 7.5 Hz, 2H), 7.31-7.20 (*m*, 6H), 4.03 (*d*, *J* = 4.4 Hz, 1H), 3.90 (*d*, *J* = 12.9 Hz, 1H), 3.74 (*s*, 1H), 3.74 (*dd*, *J* = 4.5 Hz, *J* = 9.6 Hz, 1H), 3.47 (*d*, *J* = 12.8 Hz, 1H), 3.46 (*dd*, *J* = 10.5 Hz, 1H), 3.25 (*dd*, *J* = 4.1 Hz, *J* = 10.5 Hz, 1H), 2.89 (*ddd*, *J* = 4.1 Hz, *J* = 10.5 Hz, 1H), 2.31 (*s*, 1H), 1.42 (*s*, 3H), 1.40 ppm (*s*, 3H).
^{13}C-NMR (151 MHz, $CDCl_3$): δ = 141.3, 139.2, 128.9, 128.5, 128.2, 127.4, 127.4, 127.3, 100.3, 76.9, 76.8, 74.1, 67.2, 59.6, 58.7, 29.2, 19.8 ppm.
HRMS (ESI) *m/z* calcd for $C_{21}H_{26}NO_3$ $[M + H]^+$ 340.1907, found 340.1886.

Preparation of Iminoribitol (4)

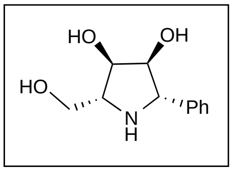

Following General Procedure **H**, a solution **18** (20 mg, 0.059 mmol) and PPTS (15 mg, 0.059 mmol) in 1:1 H_2O/MeOH (4.0 mL) was warmed to 100°C in a microwave reactor and maintained at this temperature for 30 minutes then concentrated *in vacuo*. Following General Procedure **G**, a solution of crude iminocyclitol *p*-toluenesulfonate salt (28 mg, 0.059 mmol) in MeOH (20 mL) was passed through an H-Cube reactor. Conditions: Temperature = 35°C; Flow rate = 0.8 mL/min; H_2 Pressure = 40 bar. The resulting mixture was stirred with DOWEX 1X8-100 (HO^- form) for a further 30 min and the resin was removed by filtration. Concentration of the produce mixture and purification of the crude product by flash chromatography on C_{18} silica gel (H_2O) afforded iminoribitol **4** (10 mg, 83% yield) as a colourless oil.

Data for **4**: $[\alpha]_D^{20}$ = -31 (*c* 0.48 in MeOH).
IR (neat): υ = 3306, 2918, 1560, 1494, 1454, 1406, 1347, 1081, 951, 757, 699 cm^{-1}.
^{1}H-NMR (400 MHz, CD_3OD): δ = 7.45-7.42 (*m*, 2H), 7.37-7.32 (*m*, 2H), 7.27 (*ddt*, *J* = 1.4 Hz, *J* = 6.4 Hz, *J* = 8.5 Hz, 1H), 4.01 ($_{app}$*d*, *J* = 7.2 Hz, 1H), 3.97 (*dd*, *J* = 4.7 Hz, *J* = 6.0 Hz, 1H), 3.86 (*dd*, *J* = 6.1 Hz, *J* = 7.2 Hz, 1H), 3.74 (*d*, *J* = 4.5 Hz, 2H), 3.15 ppm (*q*, *J* = 4.5 Hz, 1H).
^{13}C-NMR (151 MHz, CD_3OD): δ = 142.6, 129.5, 128.5, 128.1, 79.2, 73.6, 68.1, 66.8, 63.2 ppm.
HRMS (ESI) *m/z* calcd for $C_{11}H_{15}NO_3$ $[M + H]^+$ 210.1125, found 210.1111.

Preparation of Aldol Adduct (19a)

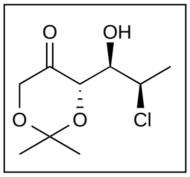

Following General Procedure **A**, a solution of propanal (450 µL, 6.18 mmol), NCS (866 mg, 6.49 mmol), (*S*)-Proline (568 mg, 4.94 mmol) and dioxanone **8** (775 µL, 6.49 mmol) in CH_2Cl_2 (30 mL) was stirred for 24 hours. Purification of the crude *syn*- and *anti*-chlorohydrins (dr = 2.5:1) by flash chromatography (pentane-EtOAc 9:1) afforded *syn*-chlorohydrin **19a** (606 mg, 44% yield) as a colourless oil**.**

Data for **19a**:[3] See reference 3.

Preparation of Chloroaminoalcohol (S53)

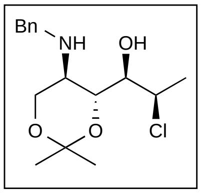

Following General Procedure **B**, a solution of **19a**[3] (200 mg, 0.898 mmol), $BnNH_2$ (245 µL, 2.25 mmol) and glacial AcOH (52.0 µL, 0.898 mmol) in THF (9.00 mL) was stirred for 1 hour then reacted with $NaCNBH_3$ (141 mg, 2.25 mmol) for 1 additional hour. Purification of the crude product by flash chromatography (pentane-EtOAc 65:35) afforded *syn*-chloroaminoalcohol **S53** (251 mg, 89% yield) as a colourless oil.

Data for **S56**: R_f (pentane-EtOAc 1:1) 0.52.
IR (neat): υ = 3280, 2999, 2962, 1452, 1378, 1261, 1197, 1095, 859, 749, 697 cm^{-1}.
^{1}H-NMR (400 MHz, $CDCl_3$): δ = 7.36-7.27 (*m*, 5H), 4.39 (*dq*, *J* = 1.2 Hz, *J* = 6.9 Hz, 1H), 4.09 (*dd*, *J* = 5.1 Hz, *J* = 11.5 Hz, 1H), 3.90 (*d*, *J* = 12.5 Hz, 1H), 3.78 (*d*, *J* = 12.5 Hz, 1H), 3.70 (*dd*, *J* = 1.3 Hz, *J* = 8.4 Hz, 1H), 3.66 (*dd*, *J* = 8.5 Hz, 1H), 3.58 (*dd*, *J* = 9.3 Hz, *J* = 11.5 Hz, 1H), 2.95 (*ddd*, *J* = 5.1 Hz, *J* = 9.3 Hz, 1H), 1.57 (*d*, *J* = 6.9 Hz, 3H), 1.46 ppm (*s*, 3H), 1.36 ppm (*s*, 3H).
^{13}C-NMR (101 MHz, $CDCl_3$): δ = 138.1, 128.7, 128.3, 127.8, 99.0, 78.2, 70.1, 63.4, 57.8, 55.8, 50.9, 28.3, 21.1, 19.4 ppm.

Preparation of Pyrrolidine (22)

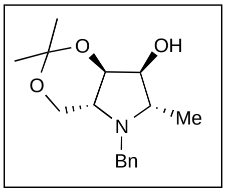

Following General Procedure **C**, a solution of **S53** (24 mg, 0.077 mmol), and $NaHCO_3$ (12 mg) in PhMe (1.0 mL) was stirred for 48 hours at 105°C. The pyrrolidine **22** (18 mg, 83% yield) was isolated as a white solid that required no further purification.

Data for **22**: mp = 86-90 °C; R_f (pentane-EtOAc 8:2) 0.33; $[\alpha]_D^{20}$ = +3.9 (*c* 0.23 in $CHCl_3$).
IR (neat): υ = 3417, 2968, 2916, 2849, 1454, 1379, 1265, 1192, 1109, 1051, 754, 703 cm^{-1}.
^{1}H-NMR (400 MHz, $CDCl_3$): δ = 7.32-7.21 (*m*, 5H), 3.89 (*d*, *J* = 13.0 Hz, 1H), 3.88 (*dd*, *J* = 1.2 Hz, *J* = 4.5 Hz, 1H), 3.63 (*dd*, *J* = 4.6 Hz, *J* = 9.9 Hz, 1H), 3.51 (*d*, *J* = 13.0 Hz, 1H), 3.41 (*dd*, *J* = 10.5 Hz, 1H), 3.31 (*dd*, *J* = 4.4 Hz, *J* = 10.5 Hz, 1H), 2.78 (*ddd*, *J* = 4.4 Hz, *J* = 10.2 Hz, 1H), 2.73 (*q*, *J* = 6.7 Hz, 1H), 2.18 (*d*, *J* = 1.2 Hz, 1H), 1.42 (*s*, 3H), 1.39 (*s*, 3H), 1.15 ppm (*d*, *J* = 6.8 Hz, 3H).
^{13}C-NMR (151 MHz, $CDCl_3$): δ = 139.5, 129.0, 128.2, 127.3, 100.1, 75.5, 73.9, 68.2, 67.2, 60.4, 59.2, 29.2, 19.9, 19.8 ppm.
HRMS (ESI) *m/z* calcd for $C_{16}H_{24}NO_3$ $[M + H]^+$ 278.1751, found 278.1735.

Preparation of Iminocyclitol (23)

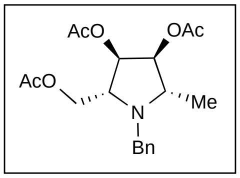

Following General Procedure **D**, a solution of **S53** (36 mg, 0.11 mmol) in MeOH (3.00 mL) was heated for 20 minutes at 120°C. The iminocyclitol HCl salt (25 mg, 81% yield) was obtained as a colourless oil that required no additional purification. To facilitate characterization, the iminocyclitol **23** was peracetylated following General Procedure **I**. Purification of the crude product by flash chromatography (pentane-EtOAc 75:25) afforded pyrrolidine **23-OAc** as a crystalline solid.

Data for **23-OAc**: mp = 55-56°C (EtOH); R_f (pentane-EtOAc 7:3) 0.25; $[\alpha]_D^{20}$ = -9.6 (*c* 0.50 in $CHCl_3$).
IR (neat): υ = 2963, 2935, 2881, 1735, 1447, 1378, 1226, 1102, 1032, 941, 908, 842, 759, 711 cm^{-1}.
^{1}H-NMR (400 MHz, $CDCl_3$): δ = 7.34-7.23 (*m*, 5H), 5.14 (*dd*, *J* = 3.4 Hz, *J* = 4.8 Hz, 1H), 4.80 (*dd*, *J* = 4.9 Hz, 7.4 Hz, 1H), 3.92-3.90 (*m*, 2H), 3.88 (*d*, *J* = 13.9 Hz, 1H), 3.79 (*d*, *J* = 14.0 Hz, 1H), 3.13 (*m*, 1H), 3.04 (*m*, 1H), 2.03 (*s*, 6H), 2.00 (*s*, 3H), 1.08 ppm (*d*, *J* = 6.2 Hz, 3H).
^{13}C-NMR (151 MHz, $CDCl_3$): δ = 170.8, 170.0, 169.9, 138.1, 129.1, 128.3, 127.3, 75.9, 72.7, 65.6, 64.0, 60.2, 56.9, 20.8, 20.8, 20.7, 18.2 ppm.
HRMS (ESI) *m/z* calcd for $C_{19}H_{26}NO_6$ $[M + H]^+$ 364.1755, found 364.1768.

Preparation of Aldol Adduct (19b)

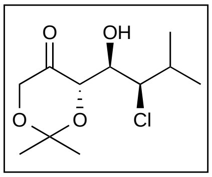

Following General Procedure **A**, a solution of isovaleraldehyde (100 μL, 0.93 mmol), NCS (131 mg, 0.98 mmol), (*S*)-Proline (85 mg, 0.74 mmol) and dioxanone **8** (117 μL, 0.98 mmol) in CH_2Cl_2 (5 mL) was stirred for 24 hours. Purification of the crude *syn*- and *anti*-chlorohydrins (dr = 22:1) 1 by flash chromatography (pentane-EtOAc 85:15) afforded *syn*-chlorohydrin **19b** (148 mg, 64% yield) as a white solid.

Data for **19b**:[3] See reference 3.

Preparation of Chloroaminoalcohol (S54)

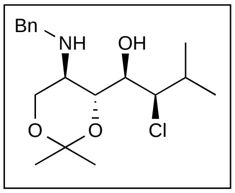

Following General Procedure **B**, a solution of **19b**[3] (100 mg, 0.400 mmol), $BnNH_2$ (109 µL, 1.00 mmol) and glacial AcOH (23 µL, 0.400 mmol) in THF (4.00 mL) was stirred for 2 hours then reacted with $NaCNBH_3$ (63 mg, 1.0 mmol) for 1 additional hour. Purification of the crude product by flash chromatography (pentane-EtOAc 65:35) afforded *syn*-chloroaminoalcohol **S54** (94 mg, 69% yield) as a colourless oil.

Data for **S57**: R_f (pentane-EtOAc 1:1) 0.77.
IR (neat): υ = 3300, 3004, 2965, 1460, 1378, 1259, 1202, 1087, 858, 750, 699 cm^{-1}.
^{1}H-NMR (600 MHz, $CDCl_3$): δ = 7.34-7.27 (*m*, 5H), 4.08 (*dd*, *J* = 5.2 Hz, *J* = 11.6 Hz, 1H), 3.94 (*dd*, *J* = 1.4 Hz, *J* = 8.4 Hz, 1H), 3.90 (*d*, *J* = 12.5 Hz, 1H), 3.89 (*dd*, *J* = 1.3 Hz, *J* = 9.3 Hz, 1H), 3.78 (*d*, *J* = 12.5 Hz, 1H), 3.73 (*dd*, *J* = 8.5 Hz, *J* = 9.7 Hz, 1H), 3.58 (*dd*, *J* = 9.2 Hz, *J* = 11.5 Hz, 1H), 2.94 (*ddd*, *J* = 5.1 Hz, *J* = 9.4 Hz, 1H), 2.15 (*m*, 1H), 1.46 (*s*, 3H), 1.36 (*s*, 3H), 1.13 (*d*, *J* = 6.7 Hz, 3H), 1.05 ppm (*d*, *J* = 6.7 Hz, 3H).
^{13}C-NMR (151 MHz, $CDCl_3$): δ = 138.3, 128.7, 128.3, 127.7, 99.1, 75.4, 70.2, 69.6, 63.5, 56.1, 50.9, 32.2, 28.3, 21.1, 20.1, 19.4 ppm.

Preparation of Pyrrolidine (24)

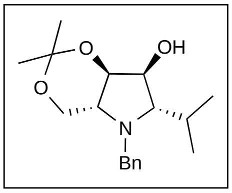

Following General Procedure **C**, a solution of **S54** (90 mg, 0.26 mmol), and $NaHCO_3$ (45 mg) in PhMe (2.6 mL) was stirred for 48 hours at 105°C. The pyrrolidine **24** (68 mg, 85% yield) was isolated as a white solid that required no further purification.

Data for **24**: mp = 59-61 °C; R_f (pentane-EtOAc 1:1) 0.9; $[\alpha]_D^{20}$ = +26 (*c* 0.70 in $CHCl_3$).
IR (neat): υ = 3445, 2955, 2872, 1455, 1380, 1209, 1109, 1025, 854, 742, 700 cm^{-1}.
^{1}H-NMR (400 MHz, $CDCl_3$): δ = 7.33-7.19 (*m*, 5H), 4.02 (*d*, *J* = 4.6 Hz, 1H), 3.99 (*d*, *J* = 13.4 Hz, 1H), 3.47 (*dd*, *J* = 4.7 Hz, *J* = 9.7 Hz, 1H), 3.44 (*d*, *J* = 13.4 Hz, 1H), 3.33 (*dd*, *J* = 10.4 Hz, 1H), 3.13 (*dd*, *J* = 4.2 Hz, *J* = 10.4 Hz, 1H), 2.74 (*ddd*, *J* = 4.2 Hz, *J* = 10.2 Hz, 1H), 2.52 (*d*, *J* = 4.7 Hz, 1H), 2.18 (*s*, 1H), 1.80 (*m*, 1H), 1.39 (*s*, 3H), 1.38 (*s*, 3H), 1.05 (*d*, *J* = 6.9 Hz, 3H), 0.97 ppm (*d*, *J* = 6.8 Hz, 3H).
^{13}C-NMR (151 MHz, $CDCl_3$): δ = 140.3, 128.5, 128.2, 127.2, 100.0, 78.4, 74.5, 70.1, 67.4, 60.7, 60.1, 29.3, 29.2, 19.9, 19.8, 17.8 ppm.
HRMS (ESI) *m/z* calcd for $C_{18}H_{28}NO_3$ $[M + H]^+$ 306.2064, found 306.2082.

Preparation of Iminocyclitol (25)

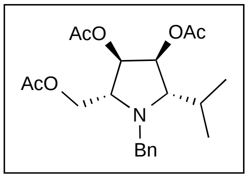

Following General Procedure **D**, a solution of **S54** (47 mg, 0.14 mmol) in MeOH (3.00 mL) was heated for 20 minutes at 120°C. The iminocyclitol HCl salt (32 mg, 78% yield) was obtained as a colourless oil that required no additional purification. To facilitate characterization, the iminocyclitol **25** was peracetylated following General Procedure **I**. Purification of the crude product by flash chromatography (pentane-EtOAc 7:3) afforded pyrrolidine **25-OAc** as a colourless oil.

Data for **25-OAc**: R_f (pentane-EtOAc 7:3) 0.4; $[\alpha]_D^{20}$ = +14 (*c* 0.40 in $CHCl_3$).
IR (neat): υ = 2958, 1744, 1454, 1368, 1228, 1066, 900, 837, 756, 701 cm^{-1}.
^{1}H-NMR (400 MHz, $CDCl_3$): δ = 7.35-7.23 (*m*, 5H), 5.16 (*dd*, *J* = 4.4 Hz, 1H), 5.09 (*dd*, *J* = 4.7 Hz, *J* = 6.2 Hz, 1H), 3.96 (*dd*, *J* = 5.3 Hz, *J* = 11.6 Hz, 1H), 3.92 (*d*, *J* = 14.1 Hz, 1H), 3.85 (*dd*, *J* = 3.6 Hz, *J* = 11.5 Hz, 1H), 3.83 (*d*, *J* = 14.0 Hz, 1H), 3.20 (*m*, 1H), 2.92 (*dd*, *J* = 4.6 Hz, 1H), 2.01 (*s*, 3H), 2.01 (*s*, 3H), 1.99 (*s*, 3H), 1.68 (*m*, 1H), 0.96 (*d*, *J* = 6.9 Hz, 3H), 0.91 ppm (*d*, *J* = 7.0 Hz, 3H).
^{13}C-NMR (151 MHz, $CDCl_3$): δ = 170.7, 169.8, 169.8, 138.5, 129.1, 128.3, 127.3, 72.9, 71.9, 71.7, 63.8, 63.5, 58.9, 29.9, 20.8, 20.8, 20.7, 19.3, 17.4 ppm.
HRMS (ESI) *m/z* calcd for $C_{21}H_{30}NO_6$ $[M + H]^+$ 392.2068, found 392.2091.

Preparation of Aldol Adduct (19c)

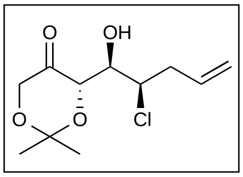

Following General Procedure **A**, a solution of 4-pentenal (100 μL, 1.01 mmol), NCS (141 mg, 1.06 mmol), (*S*)-Proline (93 mg, 0.81 mmol) and dioxanone **8** (127 μL, 1.06 mmol) in CH_2Cl_2 (4 mL) was stirred for 24 hours. Purification of the crude *syn*- and *anti*-chlorohydrins (dr = 5:1) by flash chromatography (pentane-EtOAc 9:1) afforded *syn*-chlorohydrin **19c** (130 mg, 52% yield) as a glassy solid.

Data for **19c**:[3] See reference 3.

Preparation of Chloroaminoalcohol (S55)

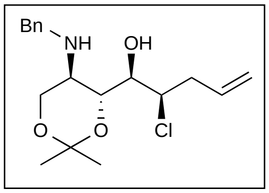

Following General Procedure **B**, a solution of **19c**[3] (1.000 g, 4.021 mmol), $BnNH_2$ (1.096 mL, 10.05 mmol) and glacial AcOH (230 μL, 4.02 mmol) in THF (40.0 mL) was stirred for 2 hours then reacted with $NaCNBH_3$ (632 mg, 10.05 mmol) for 1 hour. Purification of the crude product by flash chromatography (pentane-EtOAc 65:35) afforded *syn*-chloroaminoalcohol **S55** (1.300 g, 96% yield) as a colourless oil.

Data for **S55**: R_f (pentane-EtOAc 1:1) 0.55.
IR (neat): υ = 3602, 3107, 2976, 2921, 2884, 1640, 1455, 1378, 1220, 1206, 1109, 1023, 933, 867, 835, 699 cm^{-1}.
^{1}H-NMR (400 MHz, $CDCl_3$): δ = 7.36-7.26 (*m*, 5H), 5.87 (*m*, 1H), 5.18 (*m*, 1H), 5.13 (*m*, 1H), 4.23 (*ddd*, *J* = 1.3 Hz, *J* = 5.8 Hz, *J* = 8.9 Hz, 1H), 4.09 (*dd*, *J* = 5.2 Hz, *J* = 11.5 Hz, 1H), 3.89 (*d*, *J* = 12.6 Hz, 1H), 3.80 (*dd*, *J* = 1.3 Hz, *J* = 8.4 Hz, 1H), 3.78 (*d*, *J* = 12.6 Hz, 1H), 3.69 (*dd*, *J* = 8.5 Hz, *J* = 9.7 Hz, 1H), 3.56 (*dd*, *J* = 9.3 Hz, *J* = 11.5 Hz, 1H), 2.94 (*ddd*, *J* = 5.2 Hz, *J* = 9.5 Hz, 1H), 2.68 (*m*, 1H), 2.58 (*m*, 1H), 1.45 (*s*, 3H), 1.36 ppm (*s*, 3H).
^{13}C-NMR (125 MHz, $CDCl_3$): δ = 138.3, 134.7, 128.7, 128.2, 127.7, 117.7, 99.0, 76.7, 69.9, 63.5, 61.8, 55.9, 50.9, 38.9, 28.3, 19.3 ppm.

Preparation of Pyrrolidine (26)

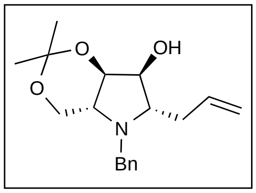

Following General Procedure **C**, a solution of **S55** (30 mg, 0.088 mmol), and $NaHCO_3$ (15 mg) in PhMe (1.0 mL) was stirred for 48 hours at 105°C. Purification of the crude product by flash chromatography (pentane-EtOAc 7:3) afforded pyrrolidine **26** (25 mg, 96% yield) as a crystalline solid.

Data for **26**: mp = 92-93 °C (EtOH); R_f (pentane-EtOAc 1:1) 0.9.
$[\alpha]_D^{20}$ = +21 (*c* 0.50 in $CHCl_3$).
IR (neat): υ = 3450, 3087, 2981, 2917, 2866, 1643, 1455, 1384, 1213, 1195, 1112, 1025, 912, 864, 837, 701, 655 cm^{-1}.
^{1}H-NMR (600 MHz, $CDCl_3$): δ = 7.32-7.22 (*m*, 5H), 5.89 (*m*, 1H), 5.12 (*m*, 1H), 5.09 (*m*, 1H), 3.99 (*dd*, *J* = 1.0 Hz, *J* = 4.5 Hz, 1H), 3.95 (*d*, *J* = 13.0 Hz, 1H), 3.56 (*dd*, *J* = 4.6 Hz, *J* = 9.8 Hz, 1H), 3.52 (*d*, *J* = 13.0 Hz, 1H), 3.38 (*dd*, *J* = 10.4 Hz, 1H), 3.25 (*dd*, *J* = 4.2 Hz, *J* = 10.4 Hz, 1H), 2.78 (*ddd*, *J* = 4.2 Hz, *J* = 10.0 Hz, 1H), 2.74 (*dd*, *J* = 4.3 Hz, *J* = 8.4 Hz, 1H), 2.27 (*m*, 1H), 2.16 (*m*, 1H), 2.15 (*s*, 1H), 1.41 (*s*, 3H), 1.39 ppm (*s*, 3H).
^{13}C-NMR (151 MHz, $CDCl_3$): δ = 139.7, 135.2, 128.8, 128.2, 127.3, 117.2, 100.1, 74.0, 73.0, 72.0, 67.2, 60.3, 59.6, 38.0, 29.2, 19.9 ppm.
HRMS (ESI) *m/z* calcd for $C_{18}H_{26}NO_3$ $[M + H]^+$ 304.1907, found 304.1914.

Preparation of Iminocyclitol (27)

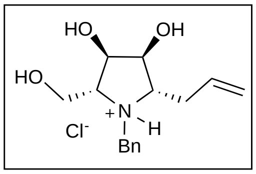

Following General Procedure **D**, a solution of **S55** (22 mg, 0.064 mmol) in MeOH (2.0 mL) was heated for 20 minutes at 120°C. Purification of the crude product by flash chromatography (CH_2Cl_2-MeOH 8:2) afforded iminocyclitol HCl salt **27** (15 mg, 79% yield) as a colourless oil. To facilitate characterization, **27** (7 mg, 0.03 mmol) was peracetylated following General Procedure **I**. Purification of the crude product by flash chromatography (pentane-EtOAc 8:2) afforded pyrrolidine **27-OAc** as a colourless oil.

Data for **27**: R_f (CH_2Cl_2-MeOH 8:2) 0.45.
IR (neat): υ = 3462, 2959, 2928, 2853, 1643, 1465, 1368, 1221, 1058, 854, 767, 703 cm^{-1}.
^{1}H-NMR (600 MHz, $CDCl_3$): δ = 7.34-7.26 (*m*, 5H), 5.81 (*m*, 1H), 5.09 (*m*, 1H), 5.08 (*m*, 1H), 4.03 (*dd*, *J* = 5.1 Hz, 1H), 3.88 (*d*, *J* = 13.4 Hz, 1H), 3.86 (*dd*, *J* = 4.8 Hz, 1H), 3.75 (*d*, *J* = 13.3 Hz, 1H), 3.44 (*dd*, *J* = 1.5 Hz, *J* = 11.1 Hz, 1H), 3.25 (*dd*, *J* = 3.8 Hz, *J* = 11.1 Hz, 1H), 2.99 (*m*, 1H), 2.94 (*m*, 1H), 2.77-2.31 (3 x *brs*, 3 x 1H), 2.23 (*m*, 1H), 2.13 ppm (*m*, 1H).
^{13}C-NMR (151 MHz, $CDCl_3$): δ = 138.6, 134.5, 129.0, 128.5, 127.6, 117.9, 74.4, 72.9, 69.8, 68.3, 60.3, 58.4, 37.3 ppm.
HRMS (ESI) *m/z* calcd for $C_{15}H_{22}NO_3$ $[M + H]^+$ 264.1594, found 264.1608.

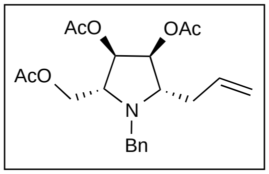

Data for **27-OAc**: R_f (CH_2Cl_2-MeOH 8:2) 0.45; $[\alpha]_D^{20}$ = +1.4 (*c* 0.36 in $CHCl_3$).
IR (neat): υ = 3462, 2959, 2928, 2853, 1643, 1465, 1368, 1221, 1058, 854, 767, 703 cm^{-1}.
^{1}H-NMR (400 MHz, $CDCl_3$): δ = 7.34-7.24 (*m*, 5H), 5.80 (*m*, 1H), 5.13 (*dd*, *J* = 4.5 Hz, 1H), 5.07-5.02 (*m*, 2H), 5.02 (*dd*, *J* = 4.7 Hz, *J* = 6.0 Hz, 1H), 3.93 (*dd*, *J* = 5.9 Hz, *J* = 11.4 Hz, 1H), 3.91 (*d*, *J* = 13.8 Hz, 1H), 3.85 (*dd*, *J* = 4.1 Hz, *J* = 11.5 Hz, 1H), 3.82 (*d*, *J* = 13.7 Hz, 1H), 3.17 (*m*, 1H), 3.11 (*m*, 1H), 2.22-2.14 (*m*, 2H), 2.03 (*s*, 3H), 2.01 (*s*, 3H), 2.01 ppm (*s*, 3H).
^{13}C-NMR (151 MHz, $CDCl_3$): δ = 170.7, 169.8, 169.8, 138.1, 133.9, 129.2, 128.3, 127.4, 117.6, 73.7, 72.7, 64.9, 64.8, 63.8, 57.9, 36.6, 20.8, 20.7, 20.7 ppm.
HRMS (ESI) *m/z* calcd for $C_{21}H_{28}NO_6$ $[M + H]^+$ 390.1911, found 390.1905.

Preparation of Aldol Adduct (19d)

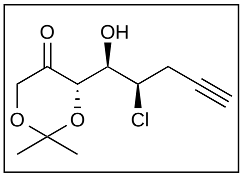

Following General Procedure **A**, a solution of 4-pentynal (2.00 g, 24.4 mmol), NCS (3.42 g, 25.6 mmol), (*S*)-proline (2.24 g, 19.5 mmol) and dioxanone **8** (3.06 mL, 25.6 mmol) in CH_2Cl_2 (120 mL) was stirred for 24 hours. Purification of the crude *syn*- and *anti*-chlorohydrins (dr = 3:1) by flash chromatography (pentane-EtOAc 85:15) afforded *syn*-chlorohydrin **19d** (3.21 g, 54% yield) as a colourless oil.

Data for **19d**:[3] See reference 3.

Preparation of Chloroaminoalcohol (S56)

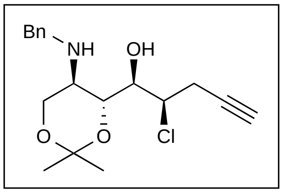

Following General Procedure **B**, a solution of **19d**[3] (125 mg, 0.507 mmol), $BnNH_2$ (138 μL, 1.27 mmol) and glacial AcOH (29 μL, 0.51 mmol) in THF (5.0 mL) was stirred for 2 hours then reacted with $NaCNBH_3$ (80 mg, 1.3 mmol) for 1 additional hour. Purification of the crude product by flash chromatography (pentane-EtOAc 65:35) afforded *syn*-chloroaminoalcohol **S56** (121 mg, 71% yield) as a colourless oil.

Data for *syn*-chloroaminoalcohol **S56**: R_f (pentane-EtOAc 1:1) 0.53; **S56** was carried forward without further characterization

Preparation of Pyrrolidine (28)

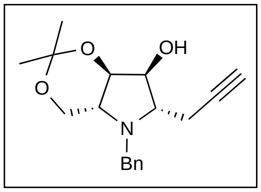

Following General Procedure **C**, a solution of **S56** (65 mg, 0.19 mmol), and $NaHCO_3$ (33 mg) in PhMe (2.0 mL) was stirred for 48 hours at 105°C. The pyrrolidine **28** (53 mg, 91% yield) was isolated as a crystalline solid that required no further purification.

Data for **28**: mp = 111-115°C (EtOH); R_f (pentane-EtOAc 8:2) 0.20.
$[\alpha]_D^{20}$ = +11 (*c* 0.74 in $CHCl_3$).
IR (neat): υ = 3446, 3292, 2990, 2917, 2879, 1454, 1382, 1268, 1210, 1195, 1121, 1026, 858, 755, 701, 644 cm^{-1}.
^{1}H-NMR (600 MHz, $CDCl_3$): δ = 7.33-7.22 (*m*, 5H), 4.14 (*d*, *J* = 4.4 Hz, 1H), 3.96 (*d*, *J* = 13.0 Hz, 1H), 3.71 (*dd*, *J* = 4.5 Hz, *J* = 9.8 Hz, 1H), 3.60 (*d*, *J* = 13.1 Hz, 1H), 3.43 (*dd*, *J* = 10.5 Hz, 1H), 3.31 (*dd*, *J* = 4.3 Hz, *J* = 10.5 Hz, 1H), 2.87-2.82 (*m*, 2H), 2.36 (*ddd*, *J* = 2.5 Hz, *J* = 4.8 Hz, *J* = 16.8 Hz, 1H), 2.23 (*ddd*, *J* = 2.5 Hz, *J* = 7.6 Hz, *J* = 16.8 Hz, 1H), 2.21 (*s*, 1H), 2.00 (*t*, *J* = 2.5 Hz, 1H), 1.42 (*s*, 3H), 1.40 ppm (*s*, 3H).
^{13}C-NMR (151 MHz, $CDCl_3$): δ = 139.3, 128.8, 128.3, 127.4, 100.2, 81.6, 73.9, 73.5, 70.7, 69.6, 67.1, 60.2, 59.5, 29.2, 23.6, 19.8 ppm.
HRMS (ESI) *m/z* calcd for $C_{18}H_{24}NO_3$ $[M + H]^+$ 302.1751, found 302.1761.

Preparation of Iminocyclitol (29)

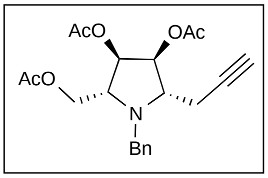

Following General Procedure **D**, a solution of **S56** (46 mg, 0.14 mmol) in MeOH (3.00 mL) was heated for 20 minutes at 120°C. The iminocyclitol HCl salt (36 mg, 88% yield) was obtained as a colourless oil that required no additional purification. To facilitate characterization, the iminocyclitol **29** was peracetylated following General Procedure **I**. Purification of the crude product by flash chromatography (pentane-EtOAc 8:2) afforded pyrrolidine **29-OAc** as a colourless oil.

Data for **29-OAc**: R_f (pentane-EtOAc 8:2) 0.35; $[\alpha]_D^{20}$ = +2.1 (*c* 0.56 in $CHCl_3$).
IR (neat): υ = 3286, 2928, 2848, 1741, 1454, 1369, 1223, 1042, 896, 755, 699, 644 cm^{-1}.
^{1}H-NMR (600 MHz, $CDCl_3$): δ = 7.33-7.25 (*m*, 5H), 5.23 (*dd*, *J* = 4.6 Hz, 1H), 5.17 (*dd*, *J* = 4.7 Hz, *J* = 5.8 Hz, 1H), 3.99 (*dd*, *J* = 6.1 Hz, *J* = 11.5 Hz, 1H), 3.95-3.89 (*m*, 2H), 3.91 (*dd*, *J* = 4.2 Hz, *J* = 11.6 Hz, 1H), 3.22 (*m*, 1H), 3.18 (*m*, 1H), 2.32 (*ddd*, *J* = 2.6 Hz, *J* = 4.3 Hz, *J* = 17.0 Hz, 1H), 2.25 (*ddd*, *J* = 2.6 Hz, *J* = 5.9 Hz, *J* = 17.1 Hz, 1H), 2.03 (*s*, 3H), 2.03 (*s*, 3H), 2.01 (*s*, 3H), 2.00 ppm (*t*, *J* = 2.6 Hz, 1H).
^{13}C-NMR (151 MHz, $CDCl_3$): δ = 170.7, 169.8, 169.8, 137.9, 129.1, 128.4, 127.5, 80.5, 74.0, 72.5, 70.5, 64.9, 64.0, 63.7, 58.0, 22.6, 20.8, 20.7, 20.7 ppm.
HRMS (ESI) *m/z* calcd for $C_{21}H_{26}NO_6$ $[M + H]^+$ 388.1755, found 388.1739.

Preparation of Aldol Adduct (19e)

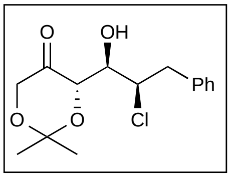

Following General Procedure **A**, a solution of hydrocinnamaldehyde (100 μL, 0.76 mmol), NCS (106 mg, 0.80 mmol), (*S*)-proline (70 mg, 0.61 mmol) and dioxanone **8** (96 μL, 0.80 mmol) in CH_2Cl_2 (3.5 mL) was stirred for 24 hours. Purification of the crude *syn*- and *anti*-chlorohydrins (dr = 3.2:1) by flash chromatography (pentane-EtOAc 85:15) afforded *syn*-chlorohydrin **19e** (144 mg, 64% yield) as a white solid.

Data for **19e**:[3] See reference 3.

Preparation of Chloroaminoalcohol (S57)

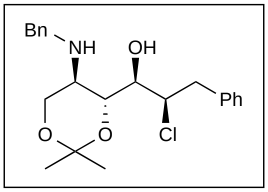

Following General Procedure **B**, a solution of **19e**[3] (100 mg, 0.335 mmol), $BnNH_2$ (91 μL, 0.84 mmol) and glacial AcOH (19 μL, 0.33 mmol) in THF (3.4 mL) was stirred for 2 hours then reacted with $NaCNBH_3$ (53 mg, 0.84 mmol) for 1 additional hour. Purification of the crude product by flash chromatography (pentane-EtOAc 65:35) afforded *syn*-chloroaminoalcohol **S57** (110 mg, 84% yield) as a colourless oil.

Data for **S57**: R_f (pentane-EtOAc 1:1) 0.67.
IR (neat): υ = 3282, 3062, 3029, 2993, 2939, 2854, 1454, 1379, 1264, 1201, 1092, 1033, 866, 751, 700 cm^{-1}.
^{1}H-NMR (500 MHz, $CDCl_3$): δ = 7.35-7.23 (*m*, 10H), 4.40 (*ddd*, *J* = 1.1 Hz, *J* = 6.9 Hz, *J* = 8.1 Hz, 1H), 4.07 (*dd*, *J* = 5.1 Hz, *J* = 11.5 Hz, 1H), 3.90 (*d*, *J* = 12.6 Hz, 1H), 3.78 (*dd*, *J* = 1.1 Hz, *J* = 8.5 Hz, 1H), 3.77 (*d*, *J* = 12.5 Hz, 1H), 3.71 (*dd*, *J* = 8.6 Hz, *J* = 9.6 Hz, 1H), 3.55 (*dd*, *J* = 9.3 Hz, *J* = 11.4 Hz, 1H), 3.19 (*dd*, *J* = 7.0 Hz, *J* = 14.1 Hz, 1H), 3.16 (*dd*, *J* = 8.2 Hz, *J* = 14.0, 1H), 2.90 (*ddd*, *J* = 5.2 Hz, *J* = 9.5 Hz, 1H), 1.43 (*s*, 3H), 1.33 ppm (*s*, 3H).
^{13}C-NMR (101 MHz, $CDCl_3$): δ = 138.2, 138.2, 129.5, 128.8, 128.3, 128.3, 127.8, 126.6, 99.1, 76.1, 70.0, 63.4, 63.2, 55.8, 50.9, 40.7, 28.2, 19.3 ppm.

Preparation of Pyrrolidine (30)

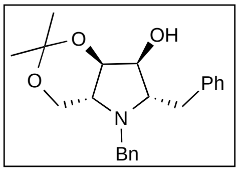

Following General Procedure **C**, a solution of **S57** (75 mg, 0.19 mmol), and $NaHCO_3$ (35 mg) in PhMe (5.0 mL) was stirred for 48 hours at 105°C. The pyrrolidine **30** (65 mg, 96% yield) was isolated as a crystalline solid that required no further purification.

Data for **30**: mp = 119-121°C (EtOH); R_f (pentane-EtOAc 8:2) 0.25.
$[\alpha]_D^{20}$ = +1.8 (*c* 0.49 in $CHCl_3$).
IR (neat): υ = 3484, 2984, 2923, 2888, 1451, 1386, 1269, 1214, 1163, 1123, 1027, 860, 743, 693, 652, 546 cm^{-1}.
^{1}H-NMR (600 MHz, $CDCl_3$): δ = 7.34-7.18 (*m*, 10H), 4.01 (*d*, *J* = 4.3 Hz, 1H), 3.85 (*d*, *J* = 12.7 Hz, 1H), 3.54 (*d*, *J* = 12.8 Hz, 1H), 3.37 (*dd*, *J* = 4.3 Hz, *J* = 9.8 Hz, 1H), 3.37 (*dd*, *J* = 10.4 Hz, 1H), 3.21 (*dd*, *J* = 4.3 Hz, *J* = 10.4 Hz, 1H), 2.96 (*dd*, *J* = 5.3 Hz, *J* = 8.0 Hz, 1H), 2.81 (*ddd*, *J* = 4.2 Hz, *J* = 10.1 Hz, 1H), 2.78 (*dd*, *J* = 5.2 Hz, *J* = 13.9 Hz, 1H), 2.67 (*dd*, *J* = 8.1 Hz, *J* = 13.9 Hz, 1H), 2.06 (*s*, 1H), 1.36 (*s*, 3H), 1.32 ppm (*s*, 3H)
^{13}C-NMR (151 MHz, $CDCl_3$): δ = 139.5, 138.7, 129.5, 129.0, 128.3, 128.3, 127.4, 126.4, 100.1, 73.8, 73.2, 73.0, 67.3, 60.2, 60.2, 40.3, 29.2, 19.7 ppm.
HRMS (ESI) *m/z* calcd for $C_{22}H_{28}NO_3$ $[M + H]^+$ 354.2064, found 354.2064.

Preparation of Iminocyclitol (31)

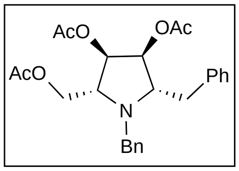

Following General Procedure **D**, a solution of **S57** (41 mg, 0.11 mmol) in MeOH (3.00 mL) was heated for 20 minutes at 120°C. The iminocyclitol HCl salt (36 mg, 97% yield) was obtained as a colourless oil that required no additional purification. To facilitate characterization, the iminocyclitol **31** was peracetylated following General Procedure **I**. Purification of the crude product by flash chromatography (pentane-EtOAc 7:3) afforded pyrrolidine **31-OAc** as a colourless oil.

Data for **31-OAc**: R_f (pentane-EtOAc 7:3) 0.59; $[\alpha]_D^{20}$ = +3.05 (*c* 1.32 in $CHCl_3$).
IR (neat): υ = 2922, 1744, 1495, 1368, 1227, 1046, 757, 701 cm^{-1}.
^{1}H-NMR (600 MHz, $CDCl_3$): δ =7.33 (*m*, 2H), 7.31-7.23 (*m*, 5H), 7.18 (*m*, 1H), 7.12 (*m*, 2H), 5.03 (*dd, J* = 4.4 Hz, 1H), 5.01 (*dd, J* = 4.5 Hz, *J* = 5.5 Hz, 1H), 3.88 (*d, J* = 13.7 Hz, 1H), 3.87-3.82 (*m*, 2H), 3.81 (*d, J* = 13.7 Hz, 1H), 3.28 (*m*, 1H), 3.19 (*m*, 1H), 2.78 (*dd, J* = 5.3 Hz, *J* = 13.8 Hz, 1H), 2.64 (*dd, J* = 7.5 Hz, *J* = 13.8 Hz, 1H), 2.00 (*s*, 3H), 1.97 (*s*, 3H), 1.80 ppm (*s*, 3H).
^{13}C-NMR (151 MHz, $CDCl_3$): δ = 170.7, 169.8, 169.7, 137.9, 137.8, 129.6, 129.4, 128.4, 128.2, 127.5, 126.4, 74.3, 72.8, 66.1, 64.4, 64.0, 58.2, 39.9, 20.8, 20.7, 20.5 ppm.
HRMS (ESI) *m/z* calcd for $C_{25}H_{30}NO_6$ $[M + H]^+$ 440.2068, found 440.2039.

Preparation of Aldol Adduct (19f)

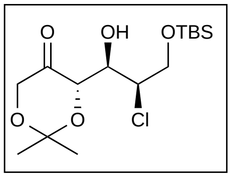

Following General Procedure **A**, a solution of 3-((*tert*-butyldimethylsilyl)oxy)-propanal[4] (508 mg, 2.70 mmol), NCS (379 mg, 2.84 mmol), (*S*)-proline (248 mg, 2.16 mmol) and dioxanone **8** (339 µL, 2.84 mmol) in CH_2Cl_2 (13 mL) was stirred for 24 hours. Purification of the crude *syn*- and *anti*-chlorohydrins (dr = 2:1) by flash chromatography (pentane-EtOAc 9:1) afforded *syn*-chlorohydrin **19f** (464 mg, 49% yield) as a colourless oil.

Data for **19f**:[3] See reference 3.

Preparation of Chloroaminoalcohol (S58)

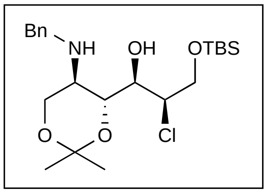

Following General Procedure **B**, a solution of **19f**[3] (97 mg, 0.27 mmol), $BnNH_2$ (75 µL, 0.69 mmol) and glacial AcOH (16 µL, 0.27 mmol) in THF (2.7 mL) was stirred for 2 hours then reacted with $NaCNBH_3$ (45 mg, 0.69 mmol) for 1 additional hour. Purification of the crude product by flash chromatography (pentane-EtOAc 9:1) afforded *syn*-chloroaminoalcohol **S58** (94 mg, 75% yield) as a colourless oil.

Data for **S58**: R_f (pentane-EtOAc 9:1) 0.29.
IR (neat): υ = 3211, 3069, 3017, 2999, 2940, 2852, 1454, 1380, 1267, 1203, 1107, 1069, 836, 777, 665 cm^{-1}.
^{1}H-NMR (600 MHz, $CDCl_3$): δ = 7.34-7.27 (*m*, 5H), 4.24 (*ddd, J* = 1.2 Hz, *J* = 6.5 Hz, *J* = 7.4 Hz, 1H), 4.09 (*dd, J* = 5.2 Hz, *J* = 11.5 Hz, 1H), 4.00 (*dd, J* = 1.2 Hz, *J* = 8.5 Hz, 1H), 3.95 (*dd, J* = 7.3 Hz, *J* = 10.4 Hz, 1H), 3.90 (*d, J* = 12.5 Hz, 1H), 3.83 (*dd, J* = 6.4 Hz, *J* = 10.4 Hz, 1H), 3.77 (*d, J* = 12.5 Hz, 1H), 3.69 (*dd, J* = 8.6 Hz, *J* = 9.7 Hz, 1H), 3.57 (*dd, J* = 9.4 Hz, *J* = 11.5 Hz, 1H), 2.94 (*ddd, J* = 5.1 Hz, *J* = 9.6 Hz, 1H), 1.46 (*s*, 3H), 1.36 (*s*, 3H), 0.91 (*s*, 9H), 0.10 (*s*, 3H), 0.09 ppm (*s*, 3H).
^{13}C-NMR (151 MHz, $CDCl_3$): δ = 138.4, 128.7, 128.3, 127.7, 99.0, 74.0, 69.6, 64.4, 63.6, 61.7, 55.9, 50.9, 28.4, 25.9, 19.3, 18.4, -5.3, -5.3 ppm.

Preparation of Pyrrolidine (32)

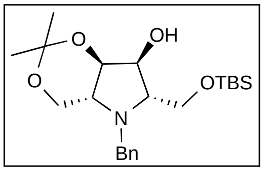

Following General Procedure **C**, a solution of **S58** (35 mg, 0.079 mmol), and $NaHCO_3$ (18 mg) in PhMe (1.0 mL) was stirred for 48 hours at 105°C. The pyrrolidine **32** (31 mg, 97% yield) was isolated as a colourless oil that required no further purification.

Data for **32**: R_f (pentane-EtOAc 8:2) 0.35; $[\alpha]_D^{20}$ = +2.1 (*c* 0.15 in $CHCl_3$).
IR (neat): υ = 3458, 2927, 2856, 1455, 1381, 1253, 1209, 1101, 1071, 835, 775, 700 cm^{-1}.
^{1}H-NMR (600 MHz, $CDCl_3$): δ = 7.32-7.21 (*m*, 5H), 4.16 (*d*, *J* = 4.3 Hz, 1H), 3.91 (*d*, *J* = 13.0 Hz, 1H), 3.63 (*d*, *J* = 13.0 Hz, 1H), 3.63 (*dd*, *J* = 4.3 Hz, *J* = 9.8 Hz, 1H), 3.52 (*dd*, *J* = 4.3 H, *J* = 10.5 Hz, 1H), 3.43 (*dd*, *J* = 10.4 Hz, 1H), 3.38 (*dd*, *J* = 6.6 Hz, *J* = 10.5 Hz, 1H), 3.32 (*dd*, *J* = 4.2 Hz, *J* = 10.4 Hz, 1H), 2.85 (*ddd*, *J* = 4.2 Hz, *J* = 10.2 Hz, 1H), 2.76 (*dd*, *J* = 4.4 Hz, *J* = 6.5 Hz, 1H), 2.16 (*s*, 1H), 1.41 (*s*, 3H), 1.40 (*s*, 3H), 0.89 (*s*, 9H), 0.03 (*s*, 3H), 0.02 ppm (*s*, 3H).
^{13}C-NMR (151 MHz, $CDCl_3$): δ = 139.7, 128.9, 128.2, 127.3, 100.0, 74.2, 73.6, 71.9, 67.2, 64.0, 60.3, 59.9, 29.2, 25.9, 19.8, 18.2, -5.4, -5.4 ppm.
HRMS (ESI) *m/z* calcd for $C_{22}H_{38}NO_4Si$ $[M + H]^+$ 408.2565, found 408.2536.

Preparation of Aldol Adduct (19g)

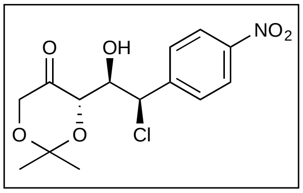

Following General Procedure **A**, a solution of (4-nitrophenyl)acetaldehyde[5] (263 mg, 1.59 mmol), NCS (183 mg, 1.59 mmol), (*S*)-Proline (128 mg, 0.956 mmol) and dioxanone **8** (207 mg, 1.59 mmol) in CH_2Cl_2 (8.00 mL) was stirred for 72 hours at 0°C. Purification of the crude *syn*- and *anti*-chlorohydrins (dr = 3.6:1) by flash chromatography (CH_2Cl_2-toluene-acetone 88.5:10:1.5) afforded chlorohydrin **19g** (251 mg, 48% yield) as a colourless oil.

Data for **19g**: R_f (CH_2Cl_2-Toluene-Acetone 88.5:10:1.5) 0.25; $[\alpha]_D^{20}$ = -88 (*c* 1.0 in $CHCl_3$).
IR (neat): υ = 3501, 3080, 2989, 2940, 2891, 1742, 1606, 1524, 1377, 1349, 1223, 1109, 1084, 1037, 857, 837, 712 cm^{-1}.
^{1}H-NMR (400 MHz, $CDCl_3$): δ = 8.22 (*d*, *J* = 8.8 Hz, 2H), 7.72 (*d*, *J* = 8.7 Hz, 2H), 5.31 (*d*, *J* = 2.0 Hz, 1H), 4.47 (*dd*, *J* = 1.5 Hz, *J* = 8.2 Hz, 1H), 4.27 (*dd*, *J* = 1.5 Hz, *J* = 17.7 Hz, 1H), 4.14 (*ddd*, *J* = 2.7 Hz, *J* = 8.2 Hz, 1H), 4.09 (*d*, *J* = 17.7 Hz, 1H), 3.41 (*dd*, *J* = 1.1 Hz, *J* = 2.9 Hz, 1H), 1.56 (*s*, 3H), 1.48 ppm (*s*, 3H).
^{13}C-NMR (151 MHz, $CDCl_3$): δ = 211.2, 147.8, 145.3, 129.4, 123.5, 101.8, 72.9, 72.9, 66.5, 60.8, 23.8, 23.5 ppm; **HRMS** (ESI) *m/z* calcd for $C_{14}H_{17}ClNO_6$ $[M + H]^+$ 330.0739, found 330.0745.

Determination of enantiomeric excess of chlorohydrin **19g**

Following General Procedure **A**, using a 1:1 mixture of (*S*):(*R*) proline, a racemic sample of the chlorohydrin **19g** was prepared. The enantiomeric chlorohydrins were separated by chiral HPLC using a DIACEL CHIRALCEL-OD-H column; flow rate 1.1 mL/min; eluent: hexanes-*i*PrOH 93:7; detection at 220 nm; retention time = 14.50 min for (-)-**19g**; 25.59 min for (+)-**19g** (see chromatograms Supplementary Table 1). The enantiomeric excess of the optically enriched chlorohydrin was determined using the same method (99% ee).

Preparation of Pyrrolidine (36)

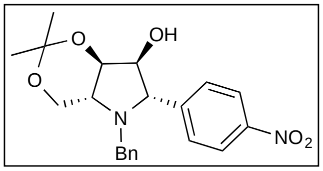

Following General Procedure **B**, a solution of **19g** (100 mg, 0.304 mmol), $BnNH_2$ (83 µL, 0.76 mmol) and glacial AcOH (17.5 µL, 0.304 mmol) in THF (3.00 mL) was stirred for 1 hour then reacted with $NaCNBH_3$ (48 mg, 0.76 mmol) for 1 additional hour. Purification of the crude product by flash chromatography (pentane-EtOAc 7:3) afforded pyrrolidine **36** (98 mg, 84% yield) as a crystalline solid.

Data for **36**: mp = 44-48°C; R_f (pentane-EtOAc 1:1) 0.75; $[\alpha]_D^{20}$ = -5.5 (*c* 0.22 in $CHCl_3$).
IR (neat): υ = 3674, 2988, 2901, 1598, 1518, 1455, 1382, 1346, 1209, 1117, 1077, 1051, 909, 857, 733, 699 cm^{-1}.
^{1}H-NMR (400 MHz, $CDCl_3$): δ = 8.21 (*d*, *J* = 8.7 Hz, 2H), 7.67 (*d*, *J* = 8.7 Hz, 2H), 7.26-7.21 (*m*, 5H), 4.00 (*d*, *J* = 4.6 Hz, 1H), 3.84 (*s*, 1H), 3.81 (*d*, *J* = 12.9 Hz, 1H), 3.70 (*dd*, *J* = 4.5 Hz, *J* = 9.8 Hz, 1H), 3.56 (*d*, *J* = 12.9 Hz, 1H), 3.53 (*dd*, *J* = 10.4 Hz, 1H), 3.36 (*dd*, *J* = 4.1 Hz, *J* = 10.4 Hz, 1H), 2.95 (*ddd*, *J* = 4.1 Hz, *J* = 10.0 Hz, 1H), 2.38 (*d*, *J* = 1.3 Hz, 1H), 1.43 (*s*, 3H), 1.41 ppm (*s*, 3H).
^{13}C-NMR (151 MHz, $CDCl_3$): δ = 149.0, 147.3, 138.2, 128.9, 128.3, 128.1, 127.7, 123.7, 100.5, 76.7, 76.1, 73.9, 66.9, 59.6, 59.0, 29.1, 19.8 ppm.
HRMS (ESI) *m/z* calcd for $C_{21}H_{25}N_2O_5$ $[M + H]^+$ 385.1758, found 385.1787.

Preparation of Aldol Adduct (19h)

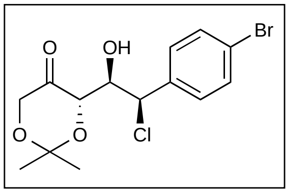

Following General Procedure **A**, a solution of 4-bromophenylacetaldehyde[5] (500 mg, 2.51 mmol), NCS (335 g, 2.51 mmol), (*S*)-proline (231 g, 2.01 mmol) and dioxanone **8** (300 µL, 2.51 mmol) in CH_2Cl_2 (13.0 mL) was stirred for 72 hours at 0°C. Purification of the crude *syn*- and *anti*-chlorohydrins (dr = 3:1) by flash chromatography (pentane-EtOAc 88:12) afforded *syn*-chlorohydrin **19h** (525 g, 58% yield) as a colourless oil.

Data for **19h**: R_f (pentane-EtOAc 85:15) 0.25; $[\alpha]_D^{20}$ -59.1 (*c* 1.8 in $CHCl_3$).
IR (neat): υ = 3484, 2989, 2117, 1741, 1489, 1376, 1223, 1109, 1084, 1011, 847 cm^{-1}.
^{1}H-NMR (400 MHz, $CDCl_3$): δ = 7.49 (*d*, *J* = 8.6 Hz, 1H), 7.40 (*d*, *J* = 8.5 Hz, 1H), 5.22 (*d*, *J* = 3.2 Hz, 1H), 4.41 (*dd*, *J* = 1.4 Hz, *J* = 7.5 Hz, 1H), 4.21 (*dd*, *J* = 1.4 Hz, *J* = 17.5 Hz, 1H), 4.14 (*dd*, *J* = 3.1 Hz, *J* = 7.5 Hz, 1H), 4.04 (*d*, *J* = 17.5 Hz, 1H), 3.32 (*s*, 1H), 1.53 (*s*, 3H), 1.47 ppm (*s*, 3H).
^{13}C-NMR (101 MHz, $CDCl_3$): δ = 210.7, 137.3, 131.5, 130.1, 122.7, 101.6, 73.5, 73.4, 66.5, 61.7, 23.7, 23.6 ppm.
HRMS (ESI) *m/z* calcd for $C_{14}H_{17}BrClO_4$ $[M + H]^+$ 362.9993, found 362.9998.

Determination of enantiomeric excess of chlorohydrin **19h**

Following General Procedure **A**, using a 1:1 mixture of (*S*):(*R*) proline, a racemic sample of the chlorohydrin **19h** was prepared. The enantiomeric chlorohydrins were separated by chiral HPLC using a DIACEL CHIRALPAK-AD column; flow rate 1.1 mL/min; eluent: hexanes-*i*PrOH 96:4; detection at 220 nm; retention time = 17.75 min for (+)-**19h**; 27.73 min for (-)-**19h** (see chromatograms in Supplementary Table 1). The enantiomeric excess of the optically enriched chlorohydrin was determined using the same method (99% ee).

Preparation of Pyrrolidine (37)

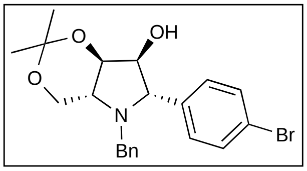

Following General Procedure **B**, a solution of **19h** (100 mg, 0.277 mmol), $BnNH_2$ (76 μL, 0.70 mmol) and glacial AcOH (16 μL, 0.28 mmol) in THF (2.8 mL) was stirred for 1 hour then reacted with $NaCNBH_3$ (43 mg, 0.69 mmol) for 1 additional hour. Purification of the crude product by flash chromatography (pentane-EtOAc 75:25) afforded pyrrolidine **37** (100 mg, 87% yield) as a white foam.

Data for **37**: R_f (pentane-EtOAc 65:35) 0.5; $[\alpha]_D^{20}$ = +11 (*c* 1.4 in $CHCl_3$).
IR (neat): υ = 3448, 3029, 2992, 2921, 2878, 1486, 1381, 1270, 1210, 1194, 1114, 1026, 1010, 979, 852, 817, 734, 700, 546, 518 cm^{-1}.
^{1}H-NMR (400 MHz, $CDCl_3$): δ = 7.49 (*d*, *J* = 8.4 Hz, 2H), 7.40 (*d*, *J* = 8.4 Hz, 2H), 7.26-7.21 (*m*, 5H), 3.98 (*d*, *J* = 4.4 Hz, 1H), 3.85 (*d*, *J* = 12.9 Hz, 1H), 3.69 (*s*, 1H), 3.68 (*dd*, *J* = 5.2 Hz, *J* = 9.0 Hz, 1H), 3.47 (*d*, *J* = 12.9 Hz, 1H), 3.46 (*dd*, *J* = 10.6 Hz, 1H), 3.26 (*dd*, *J* = 4.1 Hz, *J* = 10.5 Hz, 1H), 2.88 (*ddd*, *J* = 4.0 Hz, *J* = 10.0 Hz, 1H), 2.38 (*s*, 1H), 1.42 (*s*, 3H), 1.40 ppm (*s*, 3H).
^{13}C-NMR (151 MHz, $CDCl_3$): δ = 140.5, 138.8, 131.6, 129.1, 128.8, 128.2, 127.5, 121.1, 100.4, 76.7, 76.3, 74.0, 67.1, 59.5, 58.7, 29.2, 19.8 ppm.
HRMS (ESI) *m/z* calcd for $C_{21}H_{25}BrNO_3$ $[M + H]^+$ 418.1012, found 418.1018.

Preparation of Aldol Adduct (19i)

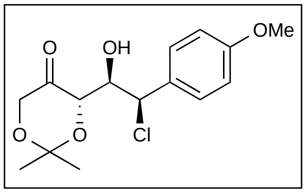

Following General Procedure **A**, a solution of 4-methoxyphenylacetaldehyde[5] (600 mg, 4.00 mmol), NCS (534 mg, 4.00 mmol), (*S*)-Proline (368 mg, 3.20 mmol) and dioxanone **8** (477 μL, 4.00 mmol) in CH_2Cl_2 (20 mL) was stirred for 168 hours at 0°C. Purification of the crude *syn*- and *anti*-chlorohydrins (dr = 3.6:1) by flash chromatography (pentane-EtOAc 9:1 to 82:18) afforded *syn*-chlorohydrin **19i** (236 mg, 19% yield; R_f (pentane-EtOAc 75:25) 0.4) as a colourless oil which was carried forward to the next reaction without further characterization

Preparation of Pyrrolidine (38)

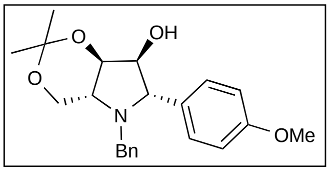

Following General Procedure **B**, a solution of **19i** (24 mg, 0.076 mmol), $BnNH_2$ (21 μL, 0.19 mmol) and glacial AcOH (4.4 μL, 0.076 mmol) in THF (0.70 mL) was stirred for 1 hour then reacted with $NaCNBH_3$ (12 mg, 0.19 mmol) for 1 additional hour. Purification of the crude product by flash chromatography (pentane-EtOAc 7:3) afforded pyrrolidine **38** (21 mg, 76% yield) as a colourless oil.

Data for **38**: R_f (pentane-EtOAc 8:2) 0.37; $[\alpha]_D^{20}$ = +11 (*c* 0.57 in $CHCl_3$).
IR (neat): υ = 3461, 2989, 2911, 1610, 1509, 1454, 1380, 1243, 1209, 1169, 1111, 1077, 1026, 977, 909, 853, 827, 732, 699 cm^{-1}.
^{1}H-NMR (400 MHz, $CDCl_3$): δ = 7.44 (*d*, *J* = 8.6 Hz, 2H), 7.28-7.19 (*m*, 5H), 6.92 (*d*, *J* = 8.7 Hz, 2H), 3.99 (*d*, *J* = 4.8 Hz, 1H), 3.89 (*d*, *J* = 13.0 Hz, 1H), 3.82 (*s*, 3H), 3.73 (*dd*, *J* = 4.8 Hz, *J* = 9.6 Hz, 1H), 3.68 (*s*, 1H), 3.44 (*dd*, *J* = 10.5 Hz, 1H), 3.43 (*d*, *J* = 13.1 Hz, 1H), 3.24 (*dd*, *J* = 4.2 Hz, *J* = 10.5 Hz, 1H), 2.86 (*ddd*, *J* = 4.3 Hz, *J* = 10.1 Hz, 1H), 2.31 (*d*, *J* = 1.3 Hz, 1H), 1.42 (*s*, 3H), 1.40 ppm (*s*, 3H).
^{13}C-NMR (101 MHz, $CDCl_3$): δ = 158.9, 139.3, 133.5, 128.8, 128.5, 128.1, 127.3, 113.9, 100.3, 76.8, 76.4, 74.2, 67.2, 59.6, 58.5, 55.3, 29.2, 19.8 ppm.
HRMS (ESI) *m/z* calcd for $C_{22}H_{28}NO_4$ $[M + H]^+$ 370.2013, found 370.2026.

Determination of enantiomeric excess of pyrrolidine **38**

Following General Procedure **A** then **B**, using a 1:1 mixture of (*S*):(*R*) proline, a racemic sample of the pyrrolidine **38** was prepared. The enantiomeric pyrrolidines were separated by chiral HPLC using a DIACEL CHIRALPAK-AD column; flow rate 0.8 mL/min; eluent: hexanes-*i*PrOH 96:4; detection at 220 nm; retention time = 19.58 min for (+)-**38**; 25.34 min for (-)-**38** (see chromatograms in Supplementary Table 1). The enantiomeric excess of the optically enriched pyrrolidine was determined using the same method (96% ee).

Preparation of Aldol Adduct (19j)

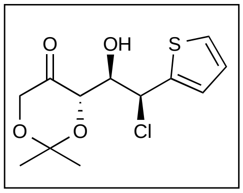

Following General Procedure **A**, a solution of 2-thienylacetaldehyde[5] (400 mg, 3.17 mmol), NCS (423 mg, 3.17 mmol), (*S*)-proline (291 mg, 2.54 mmol) and dioxanone **8** (379 μL, 3.17 mmol) in CH_2Cl_2 (16 mL) was stirred for 168 hours at 0°C. Concentration of the crude *syn*- and *anti*-chlorohydrins (dr = 1.8:1) afforded crude *syn*-chlorohydrin **19j** (142 mg, 22% yield as determined by ^{1}H NMR using an internal standard and based on unreacted starting material) as a intractable mixture which was carried forward to the next reaction without further purification or characterization.

Preparation of Pyrrolidine (39)

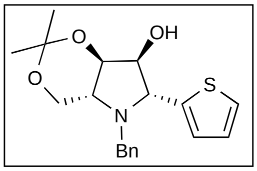

Following General Procedure **B**, a solution of **19j** (142 mg, 0.488 mmol), $BnNH_2$ (600 μL, 5.50 mmol) and glacial AcOH (126 μL, 2.19 mmol) in THF (10 mL) was stirred for 1 hour then reacted with $NaCNBH_3$ (346 mg, 5.50 mmol) for 1 additional hour. Purification of the crude product by flash chromatography (CH_2Cl_2-toluene-acetone 88.5:10:1.5) afforded pyrrolidine **39** (97 mg, 58% yield) as a colourless oil.

Data for **39**: R_f (CH_2Cl_2-toluene-acetone 88.5:10:1.5) 0.25; $[\alpha]_D^{20}$ = -0.77 (*c* 0.78 in $CHCl_3$).
IR (neat): υ = 3444, 3063, 3028, 2991, 2922, 2878, 1495, 1455, 1381, 1270, 1210, 1194, 1177, 1115, 1045, 1026, 978, 930, 853, 743, 699, 518 cm^{-1}.
^{1}H-NMR (400 MHz, $CDCl_3$): δ = 7.38-7.33 (*m*, 2H), 7.30-7.22 (*m*, 4H), 7.07 (*dt*, *J* = 1.0 Hz, *J* = 3.4 Hz, 1H), 7.01 (*dd*, *J* = 3.5 Hz, *J* = 5.1 Hz, 1H), 4.10 (*m*, 1H), 4.07 (*d*, *J* = 13.0 Hz, 1H), 4.05 (*s*, 1H), 3.81 (*dd*, *J* = 4.6 Hz, *J* = 9.5 Hz, 1H), 3.52 (*d*, *J* = 12.9 Hz, 1H), 3.42 (*dd*, *J* = 10.5 Hz, 1H) 3.15 (*dd*, *J* = 4.2 Hz, *J* = 10.6 Hz, 1H), 2.86 (*ddd*, *J* = 4.3 Hz, *J* = 10.1 Hz, 1H), 2.33 (*d*, *J* = 1.4 Hz, 1H), 1.41 (*s*, 3H), 1.39 ppm (*s*, 3H).
^{13}C-NMR (151 MHz, $CDCl_3$): δ = 147.3, 139.1, 128.9, 128.2, 127.5, 127.4, 124.5, 124.0, 100.4, 77.1, 73.9, 72.0, 67.1, 59.5, 58.9, 29.2, 19.8 ppm.
HRMS (ESI) *m/z* calcd for $C_{19}H_{24}NO_3S$ $[M + H]^+$ 346.1471, found 346.1453.

Preparation of Chloroaminoalcohol (S59)

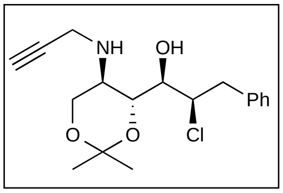

Following General Procedure **B**, a solution of **19e**[3] (300 mg, 1.00 mmol), propargylamine (161 μL, 2.50 mmol) and glacial AcOH (58 μL, 1.0 mmol) in THF (10.0 mL) was stirred for 2 hours then reacted with $NaCNBH_3$ (157 mg, 2.5 mmol) for 1 additional hour. Purification of the crude product by flash chromatography (pentane-EtOAc 7:3) afforded *syn*-chloroaminoalcohol **S59** (245 mg, 72% yield) as a colourless oil.

Data for **S59**: R_f (pentane-EtOAc 65:35) 0.45.
IR (neat): υ = 3609, 3288, 2989, 2901, 2120, 1455, 1380, 1265, 1201, 1166, 1095, 1044, 866, 760, 711, 698 cm^{-1}.
^{1}H-NMR (400 MHz, $CDCl_3$): δ = 7.37-7.21 (*m*, 5H), 4.43 (*ddd*, *J* = 1.3 Hz, *J* = 7.3 Hz, *J* = 8.1 Hz, 1H), 4.17 (*dd*, *J* = 5.1 Hz, *J* = 11.5 Hz, 1H), 3.80 (*dd*, *J* = 1.3 Hz, *J* = 8.4 Hz, 1H), 3.73 (*dd*, *J* = 8.4 Hz, *J* = 9.4 Hz, 1H), 3.61 (*dd*, *J* = 9.2 Hz, *J* = 11.6 Hz, 1H), 3.50 (*d*, *J* = 2.5 Hz, 2H), 3.19 (*dd*, *J* = 7.2 Hz, *J* = 14.1 Hz, 1H), 3.15 (*dd*, *J* = 8.3 Hz, *J* = 14.1 Hz, 1H), 2.97 (*ddd*, *J* = 5.1 Hz, *J* = 9.5 Hz, 1H), 2.27 (*t*, *J* = 2.5 Hz, 1H), 1.44 (*s*, 3H), 1.34 ppm (*s*, 3H).
^{13}C-NMR (101 MHz, $CDCl_3$): δ = 138.1, 129.5, 128.4, 126.7, 99.1, 80.7, 75.9, 72.9, 70.1, 63.3, 63.2, 56.4, 40.7, 35.9, 28.2, 19.4 ppm.

Preparation of Pyrrolidine (42)

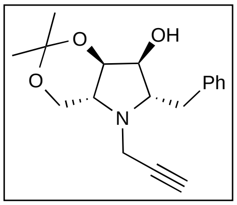

Following General Procedure **C**, a solution of **S59** (187 mg, 0.554 mmol), and $NaHCO_3$ (93 mg) in PhMe (7.0 mL) was stirred for 48 hours at 105°C. Purification of the crude product by flash chromatography (pentane-EtOAc 75:25) afforded pyrrolidine **42** (153 mg, 92% yield) as a crystalline solid.

Data for **42**: mp = 115-116°C (neat); R_f (pentane-EtOAc 8:2) 0.25.
$[\alpha]_D^{20}$ = +9.3 (*c* 0.44 in $CHCl_3$).
IR (neat): υ = 3428, 3297, 3230, 3025, 2996, 2918, 2885, 2107, 1495, 1455, 1380, 1324, 1255, 1213, 1193, 1099, 1079, 1051, 1019, 981, 933, 859, 831, 746, 698, 675, 649, 577 cm^{-1}.
^{1}H-NMR (400 MHz, $CDCl_3$): δ = 7.32-7.19 (*m*, 5H), 4.14 (*dd*, *J* = 4.2 Hz, *J* = 10.1 Hz, 1H), 4.01 (*m*, 1H), 3.75 (*dd*, *J* = 10.2 Hz, 1H), 3.39 (*dd*, *J* = 4.5 Hz, *J* = 9.9 Hz, 1H), 3.35 (*dd*, *J* = 2.2 Hz, *J* = 17.5 Hz, 1H), 3.28 (*dd*, *J* = 2.2 Hz, *J* = 17.4 Hz, 1H), 3.09 (*dd*, *J* = 5.7 Hz, *J* = 7.7 Hz, 1H), 3.01 (*ddd*, *J* = 4.2 Hz, *J* = 9.9 Hz, 1H), 2.84 (*dd*, *J* = 5.4 Hz, *J* = 13.9 Hz, 1H), 2.70 (*dd*, *J* = 8.1 Hz, *J* = 13.9 Hz, 1H), 2.24 (*t*, *J* = 2.2 Hz, 1H), 2.02 (*d*, *J* = 1.5 Hz, 1H), 1.44 (*s*, 3H), 1.41 ppm (*s*, 3H).
^{13}C-NMR (151 MHz, $CDCl_3$): δ = 138.4, 129.4, 128.3, 126.4, 100.4, 78.9, 73.6, 73.1, 73.0, 70.8, 66.2, 56.9, 40.4, 39.7, 29.2, 19.7 ppm.
HRMS (ESI) *m/z* calcd for $C_{18}H_{24}NO_3$ $[M + H]^+$ 302.1751, found 302.1754.

Preparation of Chloroaminoalcohol (S60)

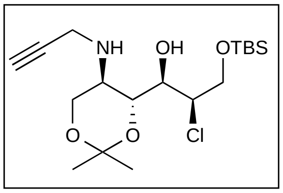

Following General Procedure **B**, a solution of **19f**[3] (100 mg, 0.28 mmol), propargylamine (45 µL, 0.71 mmol) and glacial AcOH (16 µL, 0.28 mmol) in THF (2.8 mL) was stirred for 2 hours then reacted with $NaCNBH_3$ (45 mg, 0.71 mmol) for 1 additional hour. Purification of the crude product by flash chromatography (pentane-EtOAc 75:25) afforded *syn*-chloroaminoalcohol **S60** (82 mg, 74% yield) as a colourless oil.

Data for **S60**: R_f (pentane-EtOAc 75:25) 0.8.
IR (neat): υ = 3568, 3293, 2992, 2952, 2929, 2884, 2856, 2110, 1463, 1379, 1257, 1199, 1096, 836, 777, 662 cm^{-1}.
^{1}H-NMR (500 MHz, $CDCl_3$): δ = 4.26 (*ddd*, *J* = 1.2 Hz, *J* = 6.1 Hz, *J* = 7.2 Hz, 1H), 4.16 (*dd*, *J* = 5.1 Hz, *J* = 11.6 Hz, 1H), 4.03 (*dd*, *J* = 1.2 Hz, *J* = 8.5 Hz, 1H), 3.94 (*dd*, *J* = 7.2 Hz, *J* = 10.5 Hz, 1H), 3.85 (*dd*, *J* = 6.1 Hz, *J* = 10.5 Hz, 1H), 3.67 (*dd*, *J* = 8.7 Hz, *J* = 9.6 Hz, 1H), 3.60 (*dd*, *J* = 9.2 Hz, *J* = 11.6 Hz, 1H), 3.46 (*d*, *J* = 2.5 Hz, 2H), 3.01 (*ddd*, *J* = 5.1 Hz, *J* = 9.4 Hz, 1H), 2.25 (*t*, *J* = 2.5 Hz, 1H), 1.46 (*s*, 3H), 1.36 (*s*, 3H), 0.91 (*s*, 9H), 0.09 ppm (*s*, 6H).
^{13}C-NMR (101 MHz, $CDCl_3$): δ = 99.0, 81.0, 74.3, 72.6, 69.7, 64.7, 63.3, 61.4, 56.3, 35.8, 28.3, 25.9, 19.4, 18.3, -5.4, -5.4 ppm.

Preparation of Pyrrolidine (43)

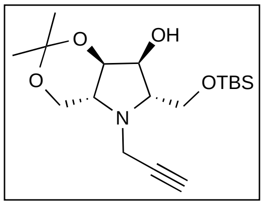

Following General Procedure **C**, a solution of **S60** (58 mg, 0.15 mmol), and $NaHCO_3$ (30 mg) in PhMe (3.0 mL) was stirred for 48 hours at 105°C. Purification of the crude product by flash chromatography (pentane-EtOAc 75:25) afforded pyrrolidine **43** (48 mg, 91% yield) as a colourless oil.

Data for **43**: R_f (pentane-EtOAc 85:15) 0.25; $[\alpha]_D^{20}$ = -4.5 (*c* 0.80 in $CHCl_3$).
IR (neat): υ = 3459, 3310, 2993, 2951, 2928, 2856, 2098, 1462, 1382, 1321, 1253, 1209, 1192, 1104, 1073, 838, 777, 666 cm^{-1}.
^{1}H-NMR (400 MHz, $CDCl_3$): δ = 4.14 (*dd*, *J* = 4.5 Hz, *J* = 10.2 Hz, 1H), 4.11 (*m*, 1H), 3.78 (*dd*, *J* = 10.3 Hz, 1H), 3.65 (*dd*, *J* = 5.2 Hz, *J* = 10.5 Hz, 1H), 3.61 (*dd*, *J* = 4.5 Hz, *J* = 10.0 Hz, 1H), 3.53 (*dd*, *J* = 5.6 Hz, *J* = 10.5 Hz, 1H), 3.51 (*dd*, *J* = 2.3 Hz, *J* = 17.4 Hz, 1H), 3.41 (*dd*, *J* = 2.3 Hz, *J* = 17.4 Hz, 1H), 3.03 (*ddd*, *J* = 4.4 Hz, *J* = 10.2 Hz, 1H), 2.89 (*dd*, *J* = 5.5 Hz, 1H), 2.24 (*t*, *J* = 2.3 Hz, 1H), 2.10 (*d*, *J* = 1.3 Hz, 1H), 1.48 (*s*, 3H), 1.46 (*s*, 3H), 0.90 (*s*, 9H), 0.06 ppm (*s*, 6H).
^{13}C-NMR (101 MHz, $CDCl_3$): δ = 100.3, 78.9, 74.0, 73.1, 71.6, 71.2, 66.1, 63.9, 56.8, 41.0, 29.2, 25.9, 19.8, 18.3, -5.3, -5.4 ppm.
HRMS (ESI) *m/z* calcd for $C_{18}H_{34}NO_4Si$ $[M + H]^+$ 356.2252, found 356.2278.

Preparation of Chloroaminoalcohol (S61)

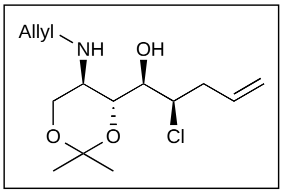

Following General Procedure **B**, a solution of **19c**[3] (50 mg, 0.20 mmol), allylamine (30 µL, 0.40 mmol) and glacial AcOH (12 µL, 0.20 mmol) in THF (2.0 mL) was stirred for 2 hours then reacted with $NaCNBH_3$ (31 mg, 0.49 mmol) for 1 additional hour. Purification of the crude product by flash chromatography (pentane-EtOAc 6:4) afforded *syn*-chloroaminoalcohol **S61** (48 mg, 83% yield) as a colourless oil.

Data for **S61**: R_f (pentane-EtOAc 1:1) 0.55.
IR (neat): υ = 3133, 2956, 2919, 2880, 1643, 1457, 1384, 1216, 1202, 1110, 1021, 929, 863, 837, 703 cm^{-1}.
^{1}H-NMR (600 MHz, $CDCl_3$): δ = 5.89 (*m*, 1H), 5.83 (*m*, 1H), 5.20 (*m*, 1H), 5.19 (*m*, 1H), 5.14 (*m*, 1H), 5.13 (*m*, 1H), 4.23 (*ddd*, *J* = 1.1 Hz, *J* = 5.8 Hz, *J* = 8.8 Hz, 1H), 4.08 (*dd*, *J* = 5.1 Hz, *J* = 11.5 Hz, 1H), 3.82 (*dd*, *J* = 1.0 Hz, *J* = 12.6 Hz, 1H), 3.69 (*dd*, *J* = 8.5 Hz, *J* = 9.8 Hz, 1H), 3.53 (*dd*, *J* = 9.5 Hz, *J* = 11.4 Hz, 1H), 3.39 (*m*, 1H), 3.23 (*m*, 1H), 2.88 (*ddd*, *J* = 5.1 Hz, *J* = 9.7 Hz, 1H), 2.68 (*m*, 1H), 2.58 (*m*, 1H), 1.46 (*s*, 3H), 1.37 ppm (*s*, 3H).
^{13}C-NMR (151 MHz, $CDCl_3$): δ = 135.2, 134.7, 117.7, 117.4, 99.0, 76.7, 69.9, 63.6, 61.9, 55.7, 49.3, 38.9, 28.4, 19.3 ppm.

Preparation of Pyrrolidine (40)

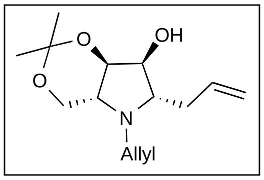

Following General Procedure **C**, a solution of **S61** (25 mg, 0.087 mmol), and $NaHCO_3$ (10 mg) in PhMe (1.0 mL) was stirred for 48 hours at 105°C. The pyrrolidine **40** (20 mg, 91% yield) was isolated as a crystalline solid that required no further purification.

Data for **40**: mp = 44-47°C (EtOH); R_f (pentane-EtOAc 6:4) 0.55.
$[\alpha]_D^{20}$ = -13 (*c* 0.68 in $CHCl_3$).
IR (neat): υ = 3438, 3076, 2993, 2873, 1641, 1437, 1380, 1318, 1266, 1210, 1194, 1115, 1029, 993, 919, 864, 838 cm^{-1}.
^{1}H-NMR (400 MHz, $CDCl_3$): δ = 5.90-5.77 (*m*, 2H), 5.26-5.05 (*m*, 4H), 4.11 (*dd*, *J* = 4.3 Hz, *J* = 10.3 Hz, 1H), 3.97 (*dd*, *J* = 1.0 Hz, *J* = 4.5 Hz, 1H), 3.74 (*dd*, *J* = 10.4 Hz, 1H), 3.56 (*dd*, *J* = 4.5 Hz, *J* = 9.8 Hz, 1H), 3.30 (*dddd*, *J* = 1.4 Hz, *J* = 5.9 Hz, *J* = 13.9 Hz, 1H), 3.18 (*m*, 1H), 2.81 (*ddd*, *J* = 4.4 Hz, *J* = 10.2 Hz, 1H), 2.65 (*dd*, *J* = 4.2 Hz, *J* = 9.2 Hz, 1H), 2.31 (*m*, 1H), 2.13 (*d*, *J* = 1.0 Hz, 1H), 2.10 (*m*, 1H), 1.48 (*s*, 3H), 1.44 ppm (*s*, 3H).
^{13}C-NMR (151 MHz, $CDCl_3$): δ = 135.9, 135.1, 117.5, 117.1, 100.2, 73.9, 72.9, 71.2, 67.0, 59.3, 57.5, 38.3, 29.2, 19.9 ppm.
HRMS (ESI) *m/z* calcd for $C_{14}H_{24}NO_3$ $[M + H]^+$ 254.1751, found 254.1778.

Preparation of Indolizidine (45)

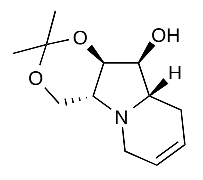

Following General Procedure **E**, a solution of **40** (5.0 mg, 0.020 mmol) and Hoveyda-Grubbs catalyst 2nd Gen. (0.6 mg, 0.001 mmol) in degassed PhMe (2.0 mL) was heated for 2 hours at 60°C. Purification of the crude product by flash chromatography (pentane-EtOAc 4:6 to EtOAc) afforded indolizidine **45** (4.2 mg, 93% yield) as a colourless oil.

Data for **45**: R_f (pentane-EtOAc 4:6) 0.44; $[\alpha]_D^{20}$ = -31 (*c* 0.25 in $CHCl_3$).
IR (neat): υ = 3438, 3033, 2993, 2917, 2849, 2774, 1644, 1461, 1380, 1256, 1211, 1192, 1126, 1086, 1012, 854, 802, 662 cm^{-1}.
^{1}H-NMR (600 MHz, $CDCl_3$): δ = 5.78 (*m*, 1H), 5.70 (*m*, 1H), 4.11 (*dd*, *J* = 4.3 Hz, *J* = 10.2 Hz, 1H), 4.01 (*dd*, *J* = 1.8 Hz, *J* = 5.9 Hz, 1H), 3.90 (*dd*, *J* = 10.0 Hz, 1H), 3.64 (*dd*, *J* = 6.1 Hz, 1H), 3.29 (*d*, *J* = 14.5 Hz, 1H), 2.99 (*d*, *J* = 14.6 Hz, 1H), 2.51 (*m*, 1H), 2.48-2.40 (*m*, 2H), 2.30 (*d*, *J* = 1.8 Hz, 1H), 2.22 (*m*, 1H), 1.51 (*s*, 3H), 1.48 ppm (*s*, 3H).
^{13}C-NMR (151 MHz, $CDCl_3$): δ = 126.0, 125.6, 101.0, 74.3, 71.8, 70.5, 65.5, 58.8, 50.3, 30.4, 29.3, 20.0 ppm.
HRMS (ESI) *m/z* calcd for $C_{12}H_{20}NO_3$ $[M + H]^+$ 226.1438, found 226.1458.

Preparation of Iminocyclitol (41)

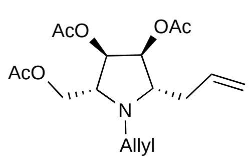

Following General Procedure **D**, a solution of **S61** (26 mg, 0.090 mmol) in MeOH (3.00 mL) was heated for 20 minutes at 120°C. The iminocyclitol HCl salt (19 mg, 83% yield) was obtained as a colourless oil which required no additional purification. To facilitate characterization, the iminocyclitol **41** was peracetylated following General Procedure **I**. Purification of the crude product by flash chromatography (pentane-EtOAc 75:25) afforded pyrrolidine **41-OAc** as a colourless oil.

Data for **41-OAc**: R_f (pentane-EtOAc 75:25) 0.35; $[\alpha]_D^{20}$ = +12.7 (*c* 1.45 in $CHCl_3$).
IR (neat): υ = 3075, 2921, 2848, 1742, 1641, 1436, 1368, 1223, 1046, 998, 919 cm^{-1}.
^{1}H-NMR (400 MHz, $CDCl_3$): δ = 5.86 (*m*, 1H), 5.80 (*m*, 1H), 5.24-5.03 (*m*, 4H), 5.14 (*dd*, *J* = 4.8 Hz, 1H), 4.99 (*dd*, *J* = 5.1 Hz, *J* = 6.1 Hz, 1H), 4.08 (*dd*, *J* = 5.5 Hz, *J* = 11.5 Hz, 1H), 4.00 (*dd*, *J* = 3.7 Hz, *J* = 11.4 Hz, 1H), 3.39 (*dd*, *J* = 6.4 Hz, *J* = 14.4 Hz, 1H), 3.30 (*dd*, *J* = 6.9 Hz, *J* = 14.3 Hz, 1H), 3.12 (*m*, 1H), 3.07 (*m*, 1H), 2.24 (*m*, 2H), 2.07 (*s*, 3H), 2.06 (*s*, 3H), 2.04 ppm (*s*, 3H).
^{13}C-NMR (151 MHz, $CDCl_3$): δ = 170.8, 169.9, 169.8, 134.2, 133.9, 118.2, 117.5, 73.7, 72.6, 64.1, 63.7, 63.7, 55.4, 36.5, 20.9, 20.8, 20.7 ppm.
HRMS (ESI) *m/z* calcd for $C_{17}H_{26}NO_6$ $[M + H]^+$ 340.1755, found 340.1773.

Preparation of Aldol Adduct (19k)

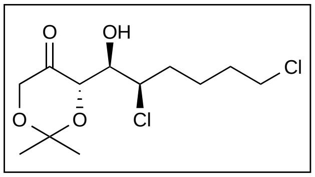

Following General Procedure **A**, a solution of 6-chlorohexanal (**46**)[6] (1.000 g, 7.429 mmol), NCS (991 mg, 7.43 mmol), (*S*)-Proline (683 mg, 5.94 mmol) and dioxanone **8** (887 μL, 7.43 mmol) in CH_2Cl_2 (37 mL) was stirred for 24 hours. Purification of the crude *syn*- and *anti*-chlorohydrins (dr = 4.4:1) by flash chromatography (pentane-EtOAc 87:13) afforded *syn*-chlorohydrin **19k** (1.359 g, 61% yield) as a colourless oil.

Data for **19k**: R_f (pentane-EtOAc 85:15) 0.35; $[\alpha]_D^{20}$ = -112 (*c* 0.630 in $CHCl_3$).
IR (neat): υ = 3517, 2989, 1736, 1376, 1222, 1159, 1088, 1036, 862, 554 cm^{-1}.
^{1}H-NMR (400 MHz, $CDCl_3$): δ = 4.41 (*dd*, *J* = 1.5 Hz, *J* = 8.8 Hz, 1H), 4.29 (*dd*, *J* = 1.5 Hz, *J* = 17.6 Hz, 1H), 4.19 (*ddd*, *J* = 1.7 Hz, *J* = 4.6 Hz, *J* = 9.4 Hz, 1H), 4.08 (*d*, *J* = 17.6 Hz, 1H), 3.93 (*dd*, *J* = 1.7 Hz, *J* = 8.8 Hz, 1H), 3.56 (*t*, *J* = 6.5 Hz, 2H), 3.39 (s_{br}, 1H), 2.02 (*m*, 1H), 1.89-1.66 (*m*, 4H), 1.59 (*m*, 1H), 1.51 (*s*, 3H), 1.42 ppm (*s*, 3H).
^{13}C-NMR (101 MHz, $CDCl_3$): δ = 212.3, 101.6, 72.7, 71.7, 66.4, 61.8, 44.7, 33.7, 32.1, 24.3, 23.8, 23.4 ppm.
HRMS (ESI) *m/z* calcd for $C_{12}H_{20}Cl_2NaO_4$ $[M + Na]^+$ 321.0631, found 321.0615.

Determination of enantiomeric excess of chlorohydrin **19k**
Following General Procedure **A**, using a 1:1 mixture of (*S*):(*R*) proline, a racemic sample of the chlorohydrin **19k** was prepared. Following General Procedure **F**, optically enriched and racemic samples of **19k** (14 mg, 0.047 mmol) were converted into the corresponding benzoyl esters. The enantiomeric benzoyl esters were separated by chiral HPLC using a DIACEL CHIRALPAK-AD column; flow rate 1.0 mL/min; eluent: hexanes-*i*PrOH 95:5; detection at 220 nm; retention time = 7.66 min for (-)-**19k-OBz**; 10.43 min for (+)-**19k-OBz** (see chromatograms in Supplementary Table 1). The enantiomeric excess of the optically enriched benzoyl ester was determined using the same method (98% ee).

Preparation of (S62)

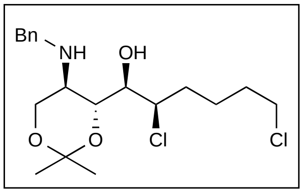

Following General Procedure **B**, a solution of **19k** (200 mg, 0.669 mmol), $BnNH_2$ (229 μL, 1.67 mmol) and glacial AcOH (38 μL, 0.67 mmol) in THF (6.7 mL) was stirred for 2 hours then reacted with $NaCNBH_3$ (105 mg, 1.67 mmol) for 1 additional hour. Purification of the crude product by flash chromatography (pentane-EtOAc 65:35) afforded *syn*-chloroaminoalcohol **S62** (260 mg; R_f (pentane-EtOAc 1:1) 0.75) as a colourless oil. The material was carried forward to the next reaction without further characterization.

Preparation of Pyrrolidine (33)

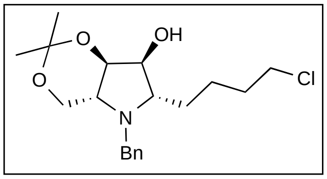

Following General Procedure **C**, a solution of **S62** (260 mg, 0.666 mmol), and $NaHCO_3$ (130 mg) in PhMe (6.6 mL) was stirred for 48 hours at 105°C. The pyrrolidine **33** (220 mg, 93% yield over two steps) was isolated as a light yellow oil that required no further purification.

Data for **33**: R_f (pentane-EtOAc 75:25) 0.37; $[\alpha]_D^{20}$ = +12 (*c* 0.58 in $CHCl_3$).
IR (neat): υ = 3457, 2989, 2836, 1454, 1381, 1209, 1194, 1120, 1075, 1026, 977, 851, 753, 701 cm^{-1}.
^{1}H-NMR (400 MHz, $CDCl_3$): δ = 7.35-7.21 (*m*, 5H), 3.96 (*d*, *J* = 4.5 Hz, 1H), 3.90 (*d*, *J* = 13.0 Hz, 1H), 3.57 (*dd*, *J* = 4.5 Hz, *J* = 9.7 Hz, 1H), 3.52 (*t*, *J* = 6.6 Hz, 2H), 3.51 (*d*, *J* = 13.0 Hz, 1H), 3.39 (*dd*, *J* = 10.4 Hz, 1H), 3.26 (*dd*, *J* = 4.3 Hz, *J* = 10.4 Hz, 1H), 2.77 (*ddd*, *J* = 4.3 Hz, *J* = 10.1 Hz, 1H), 2.61 (*dd*, *J* = 3.8 Hz, *J* = 8.8 Hz, 1H), 2.21 (*s*, 1H), 1.86-1.70 (*m*, 2H), 1.67-1.44 (*m*, 3H), 1.44-1.34 (*m*, 1H) 1.41 (*s*, 3H), 1.39 ppm (*s*, 3H).
^{13}C-NMR (151 MHz, $CDCl_3$): δ = 139.5, 128.8, 128.2, 127.4, 100.1, 74.2, 73.6, 72.7, 67.1, 60.2, 59.8, 44.8, 33.1, 32.6, 29.2, 23.6, 19.9 ppm.
HRMS (ESI) *m/z* calcd for $C_{19}H_{29}ClNO_3$ $[M + H]^+$ 354.1830, found 354.1845.

Preparation of Pyrrolidine (S63)

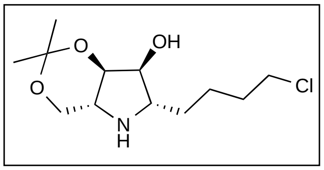

Following General Procedure **G**, a solution of **33** (30 mg, 0.085 mmol) in MeOH (15 mL) was passed through an H-Cube reactor twice. Concentration of the methanolic crude extract afforded pyrrolidine **S63**, which was carried forward to the next reaction without further purification.

Preparation of Indolizidine (S64)

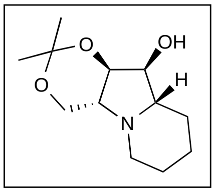

A stirred solution of **S63** (22 mg, 0.085 mmol) and $NaHCO_3$ (40 mg, 0.48 mmol) in MeOH (1 mL) was warmed to 80°C and was maintained at this temperature for 16h. The mixture was then cooled down to room temperature and the solids were filtered and triturated with CH_2Cl_2. This process was repeated and the filtrate was combined with the previous methanolic layer. The solution was concentrated under reduced pressure. Purification of the crude mixture by flash chromatography (CH_2Cl_2-MeOH 9:1) afforded indolizidine **S64** (15 mg, 79% yield over two steps) as a crystalline solid.

Data for **S64**: mp = 110-112°C (neat); R_f (CH_2Cl_2-MeOH 9:1) 0.45; $[\alpha]_D^{20}$ = -38 (*c* 0.50 in $CHCl_3$).
IR (neat): υ = 3121, 2992, 2934, 1453, 1380, 1251, 1210, 1193, 1174, 1087, 1024, 860, 722, 523 cm^{-1}.
^{1}H-NMR (400 MHz, CD_3OD): δ = 4.05 (*dd, J* = 4.4 Hz, *J* = 10.2 Hz, 1H), 3.85 (*dd, J* = 4.1 Hz, 7.1 Hz, 1H), 3.81 (*dd, J* = 10.4 Hz, 1H), 3.52 (*dd, J* = 7.1 Hz, *J* = 9.5 Hz, 1H), 2.95 (*m*, 1H), 2.31 (*ddd, J* = 4.4 Hz, *J* = 9.6 Hz, *J* = 10.4 Hz, 1H), 2.12 (*ddd, J* = 2.9 Hz, *J* = 10.9 Hz, *J* = 11.6 Hz, 1H), 2.02 (*m*, 1H), 1.97 (*m*, 1H), 1.85 (*m*, 1H), 1.65 (*m*, 1H), 1.52 (*m*, 1H), 1.48 (*s*, 3H), 1.43 (*s*, 3H) 1.46-1.33 ppm (*m*, 2H).
^{13}C-NMR (101 MHz, CD_3OD): δ = 102.0, 77.2, 75.5, 72.5, 65.9, 60.9, 52.0, 29.7, 29.7, 26.1, 26.1, 20.1 ppm.
HRMS (ESI) *m/z* calcd for $C_{12}H_{22}NO_3$ $[M + H]^+$ 228.1594, found 228.1600.

Preparation of Indolizidine (47)

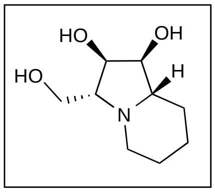

Following General Procedure **H**, a solution of **S64** (10 mg, 0.044 mmol) and PPTS (cat.) in 1:1 H_2O/MeOH (2 mL) was warmed to 100°C in a microwave reactor for 20 minutes. The mixture was cooled to room temparature, DOWEX 1X8-100 (HO^- form) was added to the flask, and was then stirred for a further 30 min. The resin was removed by filtration and concentration of the crude mixture under reduced pressure afforded indolizidine **47**[7] (7 mg, 88% yield) as an oil.

Data for **47** matches literature data:[7] $[\alpha]_D^{20}$ = -40 (*c* 0.60, H_2O) Lit. (for enantiomer of **47**) $[\alpha]_D^{22}$ = + 38 (*c* 0.48, H_2O).
^{1}H-NMR (400 MHz, D_2O): δ = 3.81 (*dd, J* = 4.6 Hz, *J* = 7.0 Hz, 1H), 3.67 (*dd, J* = 5.0 Hz, *J* = 11.7 Hz, 1H), 3.59 (*dd, J* = 6.0 Hz, *J* = 11.7 Hz, 1H), 3.49 (*dd, J* = 7.0 Hz, *J* = 9.2 Hz, 1H), 3.11 (*ddd, J* = 2.9 Hz, *J* = 10.9 Hz, 1H), 2.30 (*ddd, J* = 5.1 Hz, *J* = 10.6 Hz, 1H), 2.08 (*m*, 1H), 2.03 (*ddd, J* = 2.9 Hz, *J* = 10.9 Hz, *J* = 11.9 Hz, 1H), 1.87 (*m*, 1H), 1.74 (*m*, 1H), 1.60 (*m*, 1H), 1.37 (*qdd, J* = 4.1 Hz, *J* = 12.9 Hz, 1H), 1.21 (*qdd, J* = 3.6 Hz, *J* = 12.7 Hz, 1H), 1.15 ppm (*m*, 1H).
^{13}C-NMR (101 MHz, D_2O): δ = 73.3, 73.1, 70.8, 67.4, 61.1, 52.0, 27.6, 24.3, 23.0 ppm.

Preparation of Aldol Adduct (19l)

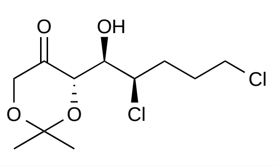

Following General Procedure **A**, a solution of 5-chloropentanal (**48**)[8] (1.00 g, 8.29 mmol), NCS (1.100 g, 8.294 mmol), (*S*)-Proline (763 mg, 6.64 mmol) and dioxanone **8** (990 μL, 8.29 mmol) in CH_2Cl_2 (41 mL) was stirred for 24 hours. Purification of the crude *syn*- and *anti*-chlorohydrins (dr = 4.4:1) by flash chromatography (pentane-EtOAc 9:1) afforded *syn*-chlorohydrin **19l** (1.229 g, 52% yield) as a colourless oil.

Data for **19l**: R_f (pentane-EtOAc 85:15) 0.33; $[\alpha]_D^{20}$ = -130 (*c* 0.590 in $CHCl_3$).
IR (neat): υ = 3514, 2989, 1737, 1376, 1221, 1159, 1086, 863, 553 cm^{-1}.
^{1}H-NMR (400 MHz, $CDCl_3$): δ = 4.41 (*dd*, *J* = 1.5 Hz, *J* = 8.7 Hz, 1H), 4.29 (*dd*, *J* = 1.5 Hz, *J* = 17.6 Hz, 1H), 4.20 (*ddd*, *J* = 1.8 Hz, *J* = 4.4 Hz, *J* = 9.9 Hz, 1H), 4.08 (*d*, *J* = 17.6 Hz, 1H), 3.94 (*dd*, *J* = 1.7 Hz, *J* = 8.7 Hz, 1H), 3.66-3.53 (*m*, 2H), 3.41 (s_{br}, 1H), 2.18-2.04 (*m*, 2H), 2.03-1.86 (*m*, 2H), 1.51 (*s*, 3H), 1.43 ppm (*s*, 3H).
^{13}C-NMR (101 MHz, $CDCl_3$): δ = 212.2, 101.7, 72.7, 71.9, 66.4, 61.3, 44.4, 31.8, 29.9, 23.8, 23.4 ppm.
HRMS (ESI) *m/z* calcd for $C_{11}H_{18}Cl_2NaO_4$ $[M + Na]^+$ 307.0474, found 307.0474.

Determination of enantiomeric excess of chlorohydrin **19l**

Following General Procedure **A**, using a 1:1 mixture of (*S*):(*R*) proline, a racemic sample of the chlorohydrin **19l** was prepared. Following General Procedure **F**, optically enriched and racemic samples of **19l** (20 mg, 0.070 mmol) were converted into the corresponding benzoyl esters. The enantiomeric benzoyl esters were separated by chiral HPLC using a DIACEL CHIRALPAK-AD column; flow rate 1.0 mL/min; eluent: hexanes-*i*PrOH 95:5; detection at 220 nm; retention time = 8.63 min for (-)-**19l-OBz**; 11.10 min for (+)-**19l-OBz** (see chromatograms in Supplementary Table 1). The enantiomeric excess of the optically enriched benzoyl ester was determined using the same method (98% ee).

Preparation of Chloroaminoalcohol (S65)

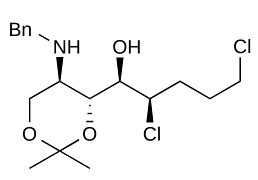

Following General Procedure **B**, a solution of **19l** (200 mg, 0.701 mmol), $BnNH_2$ (241 μL, 1.76 mmol) and glacial AcOH (40 μL, 0.70 mmol) in THF (7.0 mL) was stirred for 2 hours then reacted with $NaCNBH_3$ (110 mg, 1.75 mmol) for 1 additional hour. Purification of the crude product by flash chromatography (pentane-EtOAc 65:35) afforded *syn*-chloroaminoalcohol **S65** (240 mg; R_f (pentane-EtOAc 1:1) 0.75) as a colourless oil. The *syn*-chloroaminoalcohol **S65** was carried forward to the next reaction without further characterization

Preparation of Pyrrolizidine (49)

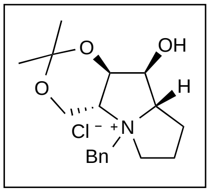

Following General Procedure **C**, a solution of **S65** (240 mg, 0.638 mmol), and $NaHCO_3$ (120 mg) in PhMe (6.4 mL) was stirred for 48 hours. The precipitate was removed by filtration and washed with MeOH (3 x 2 mL). Concentration of the filtrate afforded pyrrolizidine ammonium salt **49** (216 mg) as a white amorphous solid.

Data for **49**: **IR** (neat): υ = 3345, 3179, 2988, 2900, 1624, 1458, 1410, 1380, 1215, 1200, 1126, 1101, 1048, 1029, 993, 850, 705, 668 cm^{-1}.
^{1}H-NMR (400 MHz, CD_3OD): δ = 7.70-7.52 (*m*, 5H), 4.70 (*d*, *J* = 13.0 Hz, 1H), 4.54 (*d*, *J* = 13.0 Hz, 1H), 4.38 (*dd*, *J* = 8.6 Hz, *J* = 10.0 Hz, 1H), 4.29 (*dd*, *J* = 3.4 Hz, *J* = 10.7 Hz, 1H), 4.26 (*m*, 1H), 4.01 (*dd*, *J* = 10.4 Hz, 1H), 3.92 (*ddd*, *J* = 3.9 Hz, *J* = 10.7 Hz, 1H), 3.78 (*m*, 1H), 3.46 (*dd*, *J* = 4.0 Hz, *J* = 10.3 Hz, 1H), 3.44 (*m*, 1H), 2.52 (*m*, 1H), 2.18 (*m*, 1H), 2.10 (*m*, 1H), 1.86 (*m*, 1H), 1.52 (*s*, 3H), 1.42 ppm (*s*, 3H).
^{13}C-NMR (101 MHz, CD_3OD): δ = 133.7, 132.3, 130.7, 130.3, 102.4, 89.1, 73.2, 71.2, 70.3, 63.8, 61.4, 58.4, 30.1, 28.9, 26.1, 20.0 ppm.

Preparation of Pyrrolizidine (*S66*)

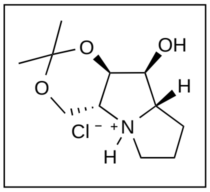

Following General Procedure **G**, a solution of **49** (216 mg, 0.636 mmol) in MeOH (15 mL) was passed through an H-Cube reactor twice. Concentration of the crude reaction mixture afforded pyrrolizidine hydrochloride salt **S66** (159 mg) as an amorphous flaky solid.

Data for **S66**: **^{1}H-NMR** (400 MHz, CD_3OD): δ = 4.28-4.16 (*m*, 4H), 4.06 (*dd*, *J* = 8.3 Hz, *J* = 10.3 Hz, 1H), 3.58 (*ddd*, *J* = 5.8 Hz, *J* = 10.7 Hz, 1H), 3.44 (*ddd*, *J* = 6.0 Hz, *J* = 10.9 Hz, 1H), 3.21 (*ddd*, *J* = 5.5 Hz, *J* = 11.3 Hz, 1H), 2.41 (*m*, 1H), 2.18 (*m*, 1H), 1.87 (*m*, 1H), 1.76 (*m*, 1H), 1.58 (*s*, 3H), 1.45 ppm (*s*, 3H).

Preparation of (50)

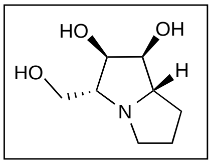

Following General Procedure **H**, a solution of **S66** (159 mg, 0.636 mmol) and PPTS (cat.) in 1:1 H_2O/MeOH (3 mL) was warmed to 100°C in a microwave reactor for 20 minutes. The mixture was cooled to room temperature, DOWEX 1X8-100 (HO^- form) was added, and the resulting mixture was stirred for a further 30 min. The resin was then removed by filtration and the filtrate was concentrated under reduced pressure to afford **(+)-7a-*epi*-hyacinthacine A_1 (50)**[9] (100 mg, 83% yield over 4 steps) as an amorphous solid.

Data for **(+)-7a-*epi*-hyacinthacine A_1** (**50**) matches literature data:[9] $[\alpha]_D^{20}$ = +42 (*c* 0.55, H_2O) Lit. $[\alpha]_D^{27}$ = + 47 (*c* 0.65, H_2O).
^{1}H-NMR (400 MHz, CD_3OD): δ = 3.89 (*dd*, *J* = 5.4 Hz, *J* = 9.0 Hz, 1H), 3.86 (*dd*, *J* = 4.3 Hz, *J* = 12.0 Hz, 1H), 3.81 (*dd*, *J* = 7.9 Hz, *J* = 12.0 Hz, 1H), 3.77 (*dd*, *J* = 2.6 Hz, *J* = 5.4 Hz, 1H), 3.40 (*ddd*, *J* = 2.5 Hz, *J* = 7.9 Hz, 1H), 3.22 (*ddd*, *J* = 4.3 Hz, *J* = 8.0 Hz, *J* = 8.8 Hz, 1H), 2.92 (*ddd*, *J* = 2.3 Hz, *J* = 6.4 Hz, *J* = 9.5 Hz, 1H), 2.79 (*ddd*, *J* = 5.7 Hz, *J* = 10.1 Hz, 1H), 2.15 (*dddd*, *J* = 2.5 Hz, *J* = 7.5 Hz, *J* = 12.5 Hz, 1H), 1.88 (*m*, 1H), 1.70 (*m*, 1H), 1.50 ppm (*dddd*, *J* = 7.5 Hz, *J* = 10.5 Hz, *J* = 12.5 Hz, 1H).
^{13}C-NMR (101 MHz, CD_3OD): δ = 77.2, 72.5, 71.5, 66.9, 61.1, 48.7, 30.9, 27.3 ppm.

Preparation of Aldol Adduct (19m)

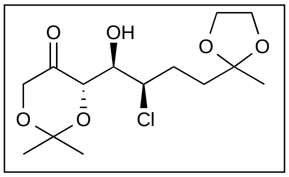

Following General Procedure **A**, a solution of 5,5-(ethylenedioxy)hexanal (**51**)[10] (1.140 g, 7.206 mmol), NCS (962 mg, 7.21 mmol), (*S*)-Proline (633 mg, 5.77 mmol) and dioxanone **8** (860 µL, 7.21 mmol) in CH_2Cl_2 (36 mL) was stirred for 24 hours. Purification of the crude *syn*- and *anti*-chlorohydrins (dr = 4.1:1) by flash chromatography (pentane-EtOAc 7:3) afforded *syn*-chlorohydrin **19m** (1.533 g, 66% yield) as a blocky crystalline solid.

Data for **19m**: mp = 50-52°C (neat); R_f (pentane-EtOAc 7:3) 0.25; $[\alpha]_D^{20}$ = -102 (*c* 1.02 in C_6H_6).
IR (neat): υ = 3494, 2980, 1739, 1375, 1250, 1220, 1098, 1037, 951, 862, 550 cm^{-1}.
^{1}H-NMR (600 MHz, C_6D_6): δ = 4.36 (*dd*, *J* = 1.3 Hz, *J* = 8.7 Hz, 1H), 4.23 (*m*, 1H), 3.78 (*ddd*, *J* = 1.9 Hz, *J* = 2.8 Hz, *J* = 8.7 Hz, 1H), 3.76 (*dd*, *J* = 1.5 Hz, *J* = 17.4 Hz, 1H), 3.58 (*d*, *J* = 17.4 Hz, 1H), 3.50-3.45 (*m*, 4H), 3.37 (*dd*, *J* = 1.2 Hz, *J* = 2.9 Hz, 1H), 2.36 (*m*, 1H), 2.07-1.99 (*m*, 2H), 1.70 (*m*, 1H), 1.30 (*s*, 3H), 1.24 (*s*, 3H), 1.08 ppm (*s*, 3H).
^{13}C-NMR (151 MHz, C_6D_6): δ = 211.8, 109.8, 101.4, 73.2, 72.2, 66.3, 64.7, 64.7, 63.1, 36.8, 29.7, 24.2, 23.8, 23.4 ppm.
HRMS (ESI) *m/z* calcd for $C_{14}H_{23}ClNaO_6$ $[M + Na]^+$ 345.1075, found 345.1082.

Determination of enantiomeric excess of chlorohydrin **19m**

Following General Procedure **A**, using a 1:1 mixture of (*S*):(*R*) proline, a racemic sample of the chlorohydrin **19m** was prepared. Following General Procedure **F**, optically enriched and racemic samples of **19m** (10 mg, 0.031 mmol) were converted into the corresponding benzoyl esters. The enantiomeric benzoyl esters were separated by chiral HPLC using a DIACEL CHIRALPAK-AD column; flow rate 1.7 mL/min; eluent: hexanes-*i*PrOH 98:2; detection at 230 nm; retention time = 10.08 min for (-)-**19m-OBz**; 11.24 min for (+)-**19m-OBz** (see chromatograms in Supplementary Table 1). The enantiomeric excess of the optically enriched benzoyl ester was determined using the same method (>98% ee).

Preparation of Chloroaminoalcohol (S67)

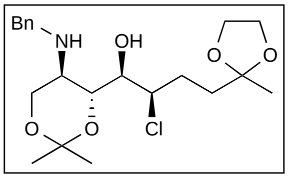

Following General Procedure **B**, a solution of **19m** (441 mg, 1.37 mmol), $BnNH_2$ (450 μL, 3.42 mmol) and glacial AcOH (70 μL, 1.37 mmol) in THF (13.7 mL) was stirred for 2 hours then reacted with $NaCNBH_3$ (200 mg, 3.42 mmol) for 1 additional hour. Purification of the crude product by flash chromatography (pentane-EtOAc 6:4) afforded *syn*-chloroaminoalcohol **S67** (466 mg, 82% yield) as a colourless oil.

Data for **S67**: R_f (pentane-EtOAc 6:4) 0.55.
IR (neat): υ = 3196, 3114, 2988, 2940, 2884, 1455, 1379, 1264, 1202, 1095, 1051, 864, 744, 700 cm^{-1}.
^{1}H-NMR (600 MHz, C_6D_6): δ = 7.12-7.02 (*m*, 5H), 4.48 (*ddd*, *J* = 1.1 Hz, *J* = 4.6 Hz, *J* = 9.0 Hz, 1H), 3.86 (*dd*, *J* = 1.3 Hz, *J* = 8.4 Hz, 1H), 3.73 (*dd*, *J* = 8.5 Hz, *J* = 9.6 Hz, 1H), 3.70 (*dd*, *J* = 5.1 Hz, *J* = 11.3 Hz, 1H), 3.49 (s_{br}, 4H), 3.25 (*d*, *J* = 12.7 Hz, 1H), 3.17 (*d*, *J* = 12.7 Hz, 1H), 3.00 (*dd*, *J* = 9.5 Hz, *J* = 11.3 Hz, 1H), 2.68 (*ddd*, *J* = 5.0 Hz, *J* = 9.5, 1H), 2.50 (*m*, 1H), 2.22-2.12 (*m*, 2H), 1.83 (*m*, 1H), 1.43 (*s*, 3H), 1.41 (*s*, 3H), 1.27 ppm (*s*, 3H).
^{13}C-NMR (151 MHz, C_6D_6): δ = 138.9, 128.9, 128.6, 127.8, 109.9, 99.0, 78.0, 70.4, 64.7, 64.7, 64.2, 63.2, 55.9, 50.7, 37.1, 30.0, 28.9, 24.3, 19.5 ppm.

Preparation of Pyrrolidine (34)

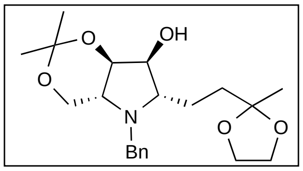

Following General Procedure **C**, a solution of **S67** (466 mg, 1.13 mmol), and $NaHCO_3$ (230 mg) in PhMe (15.0 mL) was stirred for 48 hours. The pyrrolidine **34** (316 mg, 74% yield) was isolated as a colourless oil that required no further purification.

Data for **34**: R_f (pentane-EtOAc 4:6) 0.65; $[\alpha]_D^{20}$ = +26.5 (*c* 1.01 in C_6H_6).
IR (neat): υ = 3460, 2987, 2937, 2877, 1455, 1379, 1209, 1114, 1059, 1026, 978, 850, 751, 701 cm^{-1}.
^{1}H-NMR (600 MHz, C_6D_6): δ = 7.20-7.18 (*m*, 2H), 7.10-7.02 (*m*, 3H), 3.95 (*d*, *J* = 4.6 Hz, 1H), 3.76 (*d*, *J* = 13.2 Hz, 1H), 3.57 (*dd*, *J* = 4.6 Hz, *J* = 9.7 Hz, 1H), 3.54-3.50 (*m*, 4H), 3.42 (*dd*, *J* = 10.3 Hz, 1H), 3.39 (*dd*, *J* = 4.6 Hz, *J* = 10.4 Hz, 1H), 3.27 (*d*, *J* = 13.2 Hz, 1H), 2.98 (*ddd*, *J* = 4.6 Hz, *J* = 9.9 Hz, 1H), 2.73 (*dd*, *J* = 3.4 Hz, *J* = 9.1 Hz, 1H), 2.12 (*s*, 1H), 1.92 (*m*, 1H), 1.80-1.73 (*m*, 2H), 1.62 (*m*, 1H), 1.43 (*s*, 3H), 1.28 (*s*, 3H), 1.17 ppm (*s*, 3H).
^{13}C-NMR (151 MHz, C_6D_6): δ = 140.6, 129.1, 128.4, 127.4, 110.0, 100.1, 74.9, 73.9, 73.4, 67.5, 64.7, 64.7, 61.0, 60.0, 36.3, 29.6, 28.5, 24.2, 19.8 ppm.
HRMS (ESI) *m/z* calcd for $C_{21}H_{32}NO_5$ $[M + H]^+$ 378.2275, found 378.2285.

Preparation of Iminocyclitol (S68)

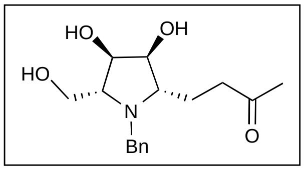

Following General Procedure **H**, a solution of **34** (32 mg, 0.085 mmol) and PPTS (cat.) in 1:1 H_2O/MeOH (1.5 mL) was warmed to 100°C in a microwave reactor for 30 minutes. The mixture was then cooled to room temperature and concentrated under reduced pressure to afford the iminocyclitol **S68** which was carried to the next reaction forward without further purification.

Preparation of (2)

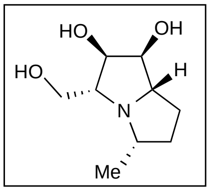

Following General Procedure **G**, a solution of **S68** (25 mg, 0.084 mmol) in MeOH (15 mL) was passed through an H-Cube reactor twice. The resulting mixture was then stirred with DOWEX 1X8-100 (HO^- form) for a further 30 min. The resin removed by filtration and the filtrate was concentrated under reduced pressure to afford pyrrolizidine **2**[11] (14 mg, 88% yield over 2 steps) as an oil.

Data for **2** matches literature data:[11] $[\alpha]_D^{20}$ = + 13 (*c* 0.35, MeOH) Litt. (for enantiomer of **2**) $[\alpha]_D^{29}$ = - 15 (*c* 0.44, MeOH).
^{1}H-NMR (400 MHz, CD_3OD): δ = 4.10 (*dd*, *J* = 4.7 Hz, *J* = 7.0 Hz, 1H), 3.73 (*dd*, *J* = 4.5 Hz, *J* = 11.4 Hz, 1H), 3.70 (*dd*, *J* = 4.6 Hz, *J* = 11.4 Hz, 1H), 3.62 (*dd*, *J* = 7.2 Hz, *J* = 8.7 Hz, 1H), 2.75 (*ddd*, *J* = 5.8 Hz, *J* = 8.7 Hz, *J* = 10.5 Hz, 1H), 2.51 (sex_{br}, *J* = 6.3 Hz, 1H), 2.41 (*q*, *J* = 4.5 Hz, 1H), 2.24 (*dddd*, *J* = 7.9 Hz, *J* = 9.1 Hz, *J* = 12.8 Hz, 1H), 1.79 (*dddd*, *J* = 2.6 Hz, *J* = 5.7 Hz, *J* = 9.1 Hz, *J* = 11.7 Hz, 1H), 1.65 (*dddd*, *J* = 2.6 Hz, *J* = 7.4 Hz, *J* = 10.7 Hz, *J* = 12.8 Hz, 1H), 1.45 (dq_{br}, *J* = 7.5 Hz, *J* = 11.1 Hz, 1H), 1.19 ppm (*d*, *J* = 6.1 Hz, 3H).
^{13}C-NMR (151 MHz, CD_3OD): δ = 78.3, 76.1, 72.3, 71.7, 63.2, 56.8, 37.7, 24.8, 21.2 ppm.

Preparation of Aldol Adduct (19n)

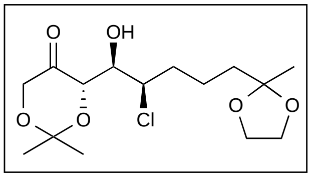

Following General Procedure **A**, a solution of 6,6-(ethylenedioxy)heptanal (**52**)[12] (232 mg, 1.35 mmol), NCS (180 mg, 1.35 mmol), (*S*)-Proline (124 mg, 1.08 mmol) and dioxanone **8** (161 µL, 1.35 mmol) in CH_2Cl_2 (7.0 mL) was stirred for 24 hours. Purification of the crude *syn*- and *anti*-chlorohydrins (dr = 4.1:1) by flash chromatography (pentane-EtOAc 75:25) afforded *syn*-chlorohydrin **19n** (292 mg, 64% yield) as a colourless oil.

Data for **19n**: R_f (pentane-EtOAc 75:25) 0.30; $[\alpha]_D^{20}$ = -95 (*c* 0.54 in C_6H_6).
IR (neat): υ = 3509, 2986, 1737, 1376, 1222, 1083, 1037, 948, 863, 530 cm^{-1}.
^{1}H-NMR (400 MHz, C_6D_6): δ = 4.38 (*dd*, *J* = 1.5 Hz, *J* = 8.7 Hz, 1H), 4.21 (*dddd*, *J* = 1.5 Hz, *J* = 4.5 Hz, *J* = 9.1 Hz, 1H), 3.81 (*ddd*, *J* = 1.7 Hz, *J* = 3.0 Hz, *J* = 8.7 Hz, 1H), 3.76 (*dd*, *J* = 1.5 Hz, *J* = 17.5 Hz, 1H), 3.59 (*d*, *J* = 17.5 Hz, 1H), 3.54-3.46 (*m*, 4H), 3.41 (*dd*, *J* = 1.3 Hz, *J* = 3.0 Hz, 1H), 2.12 (*m*, 1H), 1.85-1.69 (*m*, 2H), 1.68-1.49 (*m*, 3H), 1.31 (*s*, 3H), 1.25 (*s*, 3H), 1.07 ppm (*s*, 3H).
^{13}C-NMR (101 MHz, C_6D_6): δ = 211.9, 110.0, 101.4, 73.2, 72.1, 66.3, 64.7, 64.7, 62.8, 39.0, 35.0, 24.1, 23.8, 23.4, 21.9 ppm.
HRMS (ESI) *m/z* calcd for $C_{15}H_{25}ClNaO_6$ $[M + Na]^+$ 359.1232, found 359.1242.

Determination of enantiomeric excess of chlorohydrin **19n**

Following General Procedure **A**, using a 1:1 mixture of (*S*):(*R*) proline, a racemic sample of the chlorohydrin **19n** was prepared. Following General Procedure **F**, optically enriched and racemic samples of **19n** (14 mg, 0.047 mmol) were converted into the corresponding benzoyl esters. The enantiomeric benzoyl esters were separated by chiral HPLC using a DIACEL CHIRALPAK-AD column; flow rate 1.7 mL/min; eluent: hexanes-*i*PrOH 98:2; detection at 220 nm; retention time = 9.97 min for (-)-**19n-OBz**; 12.73 min for (+)-**19n-OBz** (see chromatograms in Supplementary Table 1). The enantiomeric excess of the optically enriched benzoyl ester was determined using the same method (96% ee).

Preparation of Chloroaminoalcohol (S69)

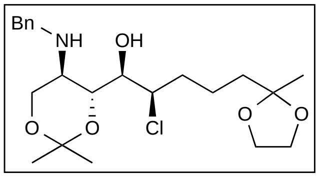

Following General Procedure **B**, a solution of **19n** (200 mg, 0.594 mmol), $BnNH_2$ (203 µL, 1.48 mmol) and glacial AcOH (34 µL, 0.59 mmol) in THF (6.0 mL) was stirred for 2 hours then reacted with $NaCNBH_3$ (93 mg, 1.48 mmol) for 1 additional hour. Purification of the crude product by flash chromatography (pentane-EtOAc 6:4) afforded *syn*-chloroaminoalcohol **S69** (200 mg, 79% yield) as a colourless oil.

Data for **S69**: R_f (pentane-EtOAc 6:4) 0.5.
IR (neat): υ = 3293, 2991, 2927, 2888, 1457, 1381, 1258, 1209, 1103, 1050, 866, 750, 699 cm^{-1}.
^{1}H-NMR (400 MHz, C_6D_6): δ = 7.12-7.01 (*m*, 5H), 4.48 (*ddd*, *J* = 1.3 Hz, *J* = 4.9 Hz, *J* = 9.5 Hz, 1H), 3.89 (*dd*, *J* = 1.4 Hz, *J* = 8.2 Hz, 1H), 3.71 (*dd*, *J* = 8.3 Hz, *J* = 9.9 Hz, 1H), 3.70 (*dd*, *J* = 4.9 Hz, *J* = 11.4 Hz, 1H), 3.53-3.47 (*m*, 4H), 3.22 (*d*, *J* = 12.7 Hz, 1H), 3.14 (*d*, *J* = 12.7 Hz, 1H), 2.95 (*dd*, *J* = 9.7 Hz, *J* = 11.2 Hz, 1H), 2.66 (*ddd*, *J* = 5.1 Hz, *J* = 9.8, 1H), 2.30 (*m*, 1H), 1.99-1.84 (*m*, 2H), 1.74-1.62 (*m*, 3H), 1.43 (*s*, 3H), 1.40 (*s*, 3H), 1.27 ppm (*s*, 3H).
^{13}C-NMR (151 MHz, C_6D_6): δ = 139.1, 128.8, 128.5, 127.7, 110.1, 99.0, 77.8, 70.5, 64.7, 64.7, 64.0, 63.4, 56.0, 50.8, 39.2, 35.4, 28.9, 24.1, 22.2, 19.5 ppm.

Preparation of Pyrrolidine (35)

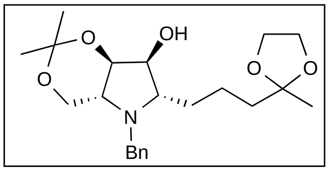

Following General Procedure **C**, a solution of **S69** (197 mg, 0.460 mmol), and $NaHCO_3$ (100 mg) in PhMe (4.6 mL) was stirred for 48 hours at 105°C. The pyrrolidine **35** (162 mg, 90% yield) was isolated as a light yellow oil that required no further purification.

Data for **35**: R_f (pentane-EtOAc 55:45) 0.45; $[\alpha]_D^{20}$ = +29 (*c* 0.26 in C_6H_6).
IR (neat): υ = 3463, 2986, 2940, 2875, 1454, 1374, 1241, 1209, 1194, 1114, 1063, 1025, 978, 852, 744, 701 cm^{-1}.
^{1}H-NMR (400 MHz, C_6D_6): δ = 7.23-7.18 (*m*, 2H), 7.12-7.02 (*m*, 3H), 3.89 (*d*, *J* = 4.5 Hz, 1H), 3.74 (*d*, *J* = 13.1 Hz, 1H), 3.55 (*dd*, *J* = 4.6 Hz, *J* = 9.7 Hz, 1H), 3.54 (*s*, 4H), 3.43 (*dd*, *J* = 10.2 Hz, 1H), 3.37 (*dd*, *J* = 4.6 Hz, *J* = 10.3 Hz, 1H), 3.27 (*d*, *J* = 13.1 Hz, 1H), 2.97 (*ddd*, *J* = 4.6 Hz, *J* = 9.9 Hz, 1H), 2.69 (*dd*, *J* = 4.3 Hz, *J* = 9.1 Hz, 1H), 2.13 (*s*, 1H), 1.78-1.64 (*m*, 3H), 1.58 (*m*, 1H), 1.47 (*m*, 1H), 1.44 (*s*, 3H), 1.34 (*m*, 1H), 1.32 (*s*, 3H), 1.21 ppm (*s*, 3H).
^{13}C-NMR (101 MHz, C_6D_6): δ = 140.7, 129.1, 128.4, 127.4, 110.1, 100.1, 74.9, 74.0, 73.4, 67.5, 64.7, 64.7, 60.9, 60.1, 39.8, 34.7, 29.6, 24.1, 21.5, 19.8 ppm.
HRMS (ESI) *m/z* calcd for $C_{22}H_{34}NO_5$ $[M + H]^+$ 392.2431, found 392.2440.

Preparation of Iminocyclitol (S70)

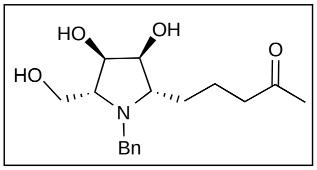

Following General Procedure **H**, a solution of **35** (30 mg, 0.077 mmol) and PPTS (cat.) in 1:1 H_2O/MeOH (1.5 mL) was warmed to 100°C in a microwave reactor for 20 minutes. The mixture was cooled to room temperature and concentrated under reduced pressure to afford pyrrolizidine **S70**, which was carried forward to the next reaction without further purification.

Preparation of Indolizidine ((*ent*)-3)

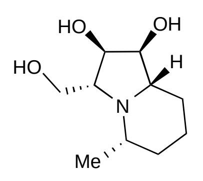

Following General Procedure **G**, a solution of **S70** (24 mg, 0.077 mmol) in MeOH (15 mL) was passed through an H-Cube reactor twice. The resulting mixture was then stirred with DOWEX 1X8-100 (HO^- form) for a further 30 min. The resin was removed by filtration and the filtrate was concentrated under reduced pressure. Purification by recrystallization (EtOH) afforded indolizidine (*ent*)-**3** (14 mg, 93% yield over 2 steps) as colourless needles.

Data for **55**: mp = 112-114°C (EtOH); $[\alpha]_D^{20}$ = -16 (*c* 0.28 in MeOH).
IR (neat): υ = 3350, 2930, 2856, 1457, 1380, 1213, 1134, 1075, 1038, 863, 718 cm^{-1}.
^{1}H-NMR (400 MHz, CD_3OD): δ = 3.92 (*d*, *J* = 4.9 Hz, 1H), 3.59 (*dd*, *J* = 3.0 Hz, *J* = 11.2 Hz, 1H), 3.48 (*dd*, *J* = 4.9 Hz, *J* = 10.0 Hz, 1H), 3.28 (*dd*, *J* = 7.9 Hz, *J* = 11.2 Hz, 1H), 2.72 (*dd*, *J* = 2.9 Hz, *J* = 7.9 Hz, 1H), 2.46 (*ddd*, *J* = 2.0 Hz, *J* = 10.2 Hz, 1H), 2.42 (*m*, 1H), 1.89 (*m*, 1H), 1.79 (*m*, 1H), 1.58 (*m*, 1H), 1.35 (*qt*, *J* = 3.9 Hz, *J* = 12.9 Hz, 1H), 1.24-1.12 (*m*, 2H), 1.09 ppm (*d*, *J* = 6.3 Hz, 3H).
^{13}C-NMR (101 MHz, CD_3OD): δ = 75.7, 75.2, 72.1, 67.8, 65.8, 60.9, 36.6, 29.4, 25.6, 22.0 ppm; **HRMS** (ESI) *m/z* calcd for $C_{10}H_{20}NO_3$ $[M + H]^+$ 202.1438, found 202.1433.

Supplementary References

1 G. M. Sheldrick, *SHELXT v2013*, Bruker AXS Inc., Madison, WI, 53711, USA.
2 C. B. Huebschle, G. M. Sheldrick, B. Dittrich, *J. Appl. Crystallogr.* **2011**, *44*, 1281-1284.
3 M. Bergeron-Brlek, T. Teoh, R. Britton, *Org. Lett.* **2013**, *15*(14), 3554-3557.
4. J. Wang, R. P. Hsung and S. K. Ghosh, *Org. Lett.* **2004**, *6*(12), 1939-1942.
5. 4-nitrophenylacetaldehyde, 4-methoxyphenylacetaldehyde, 4-bromophenylacetaldehyde and 2-thienylacetaldehyde were prepared according to: G. Relevant, S. Dunand, S. Hesse, G. Kirsch, *Synthesis* **2004**, *18*, 2935-2940.
6. Commercially available 6-chlorohexanal was prepared according to: F. Abels, C. Schneider, Synthesis **2011**, *24*, 4050-4058.
7 L. Gómez, X. Garrabou, J. Joglar, J. Bujons, T. Parella, C. Vilaplana, P. J. Cardona, P. Clapés, *Org. Biomol. Chem.* **2012**, *10*, 6309-6321.
8. Commercially available 5-chloropentanal was prepared according to: Y. Kimura, K. Yamatsugu, M. Kanai, N. Echigo, T. Kuzuhara, M. Shibasaki, *Bull. Korean Chem. Soc.* **2010**, *31*(3), 588-594.
9 I. Izquierdo, M. T. Plaza, J. A. Tamayo, F. Franco, F. Sánchez-Cantalejo, *Tetrahedron* **2010**, *66*(21), 3788-3794.
10. Commercially available 5,5-(ethylenedioxy)hexanal was prepared according to: F. A. Davis, H. Zhang, S. H. Lee, *Org. Lett.* **2001**, *3*(5), 759-762.
11 I. Izquierdo, M. T. Plaza, J. A. Tamayo, M. RodrÍguez, A. Martos, *Tetrahedron* **2006**, *62*(25), 6006-6011.
12. Commercially available 6,6-(ethylenedioxy)heptanal was prepared according to: F. A. Davis, S. H. Lee, H. Xu, *J. Org. Chem.* **2004**, *69*(11), 3774-3781.
